# Mesenchymal–epithelial transition and AXL inhibitor TP-0903 sensitise triple-negative breast cancer cells to the antimalarial compound, artesunate

**DOI:** 10.1038/s41598-023-50710-3

**Published:** 2024-01-03

**Authors:** Mirko Terragno, Anastassiya Vetrova, Oleg Semenov, A. Emre Sayan, Marina Kriajevska, Eugene Tulchinsky

**Affiliations:** 1 Department of Biomedical Sciences, School of Medicine, Nazarbayev University, Astana, Kazakhstan; 2 Regulation of Gene Expression Laboratory, Institute of Cytology RAS, Saint Petersburg, Russia; 3 Cancer Sciences Division, University of Southampton, Southampton, UK; 4 Department of Genetics and Genome Biology, University of Leicester, Leicester, UK

**Keywords:** Cancer, Molecular medicine

## Abstract

Triple-negative breast cancer (TNBC) is a difficult-to-treat, aggressive cancer type. TNBC is often associated with the cellular program of epithelial-mesenchymal transition (EMT) that confers drug resistance and metastasis. EMT and reverse mesenchymal-epithelial transition (MET) programs are regulated by several signaling pathways which converge on a group of transcription factors, EMT- TFs. Therapy approaches could rely on the EMT reversal to sensitise mesenchymal tumours to compounds effective against epithelial cancers. Here, we show that the antimalarial ROS-generating compound artesunate (ART) exhibits higher cytotoxicity in epithelial than mesenchymal breast cancer cell lines. Ectopic expression of EMT-TF ZEB1 in epithelial or ZEB1 depletion in mesenchymal cells, respectively, reduced or increased ART-generated ROS levels, DNA damage and apoptotic cell death. In epithelial cells, ZEB1 enhanced expression of superoxide dismutase 2 (SOD2) and glutathione peroxidase 8 (GPX8) implicated in ROS scavenging. Although SOD2 or GPX8 levels were unaffected in mesenchymal cells in response to ZEB1 depletion, stable ZEB1 knockdown enhanced total ROS. Receptor tyrosine kinase AXL maintains a mesenchymal phenotype and is overexpressed in TNBC. The clinically-relevant AXL inhibitor TP-0903 induced MET and synergised with ART to generate ROS, DNA damage and apoptosis in TNBC cells. TP-0903 reduced the expression of GPX8 and SOD2. Thus, TP-0903 and ZEB1 knockdown sensitised TNBC cells to ART, likely via different pathways. Synergistic interactions between TP-0903 and ART indicate that combination approaches involving these compounds can have therapeutic prospects for TNBC treatment.

## Introduction

Triple-negative breast cancer (TNBC), by definition, lacks the expression of estrogen, progesterone, and HER2 receptors. Therefore, therapies targeting these receptors, which are effective in receptor- positive breast cancer subtypes, cannot be applied to TNBC. Currently, no targeted therapy approaches are designed specifically for TNBC. Surgery, chemotherapy, and radiation are the only methods for treating these patients^1^. Because of the drug resistance, rapid recurrence, and high metastatic propensity, women with TNBC have much lower overall and relapse-free survival than other breast cancer patients^2^. There is an urgent need to develop new approaches to treat this cancer type. On the molecular level, TNBC is highly heterogeneous and includes at least six molecular subtypes^3^. Most TNBC fit in basal-like, BL1 (Basal-Like 1) and BL2 (Basal-Like 2) or mesenchymal ML (Mesenchymal-Like) and MSL (Mesenchymal/Stem-Like) categories^4,5^. Two mesenchymal-like groups, ML and MSL, constitute a most dedifferentiated claudin-low subtype. This subtype is closely related to metaplastic breast cancer which is characterised by chemoresistance, worse survival and features of epithelial-mesenchymal transition (EMT)^6^.

EMT and a process of mesenchymal-epithelial transition (MET) are reversible differentiation programs operating at different stages of embryonic development. Elements of these programs are hijacked by metastatic cancer cells during different stages of the invasion-metastasis cascade, such as intravasation (EMT) or colonisation of distant organs (MET). EMT is governed by transcription factors collectively termed EMT-TFs. EMT-TFs belonging to the Zn-finger (SNAIL and ZEB) and basic helix-loop-helix (TWIST1 and TWIST2) protein families are best studied in the context of cancer metastasis^7,8^. Enhanced invasiveness and motility are not the only features through which EMT contributes to cancer metastases. Equally important is the role of EMT-TFs in suppressing apoptosis via interplay with the p53 pathway or repression of proapoptotic genes, such as BCL2L11/BIM^9,10^. Moreover, one of the EMT hallmarks is the attenuated G1-S cell cycle transition that makes cancer cells less sensitive to genotoxic stresses and anti-proliferative therapies^11^. Several strategies for tackling EMT-prone breast cancer subtypes are being considered, including the stimulation of MET programs. This approach moves cancer cells toward the epithelial end of the EMT/MET spectrum, sensitising tumours to the chemo-, radio- or immune therapies which are effective against epithelial tumours. In this context, the receptor tyrosine kinase (RTK) AXL is an attractive target molecule whose expression is essential for maintaining mesenchymal phenotype in different cancers, including TNBC^12–14^. AXL is a member of the TAM family (also includes TYRO3 and MER) and is activated after binding the ligand, vitamin K-dependent protein GAS6 (Growth Arrest-Specific gene 6)^12^. In normal physiological conditions, AXL is expressed on professional phagocytes, such as dendritic cells and some macrophage populations. AXL interaction with GAS6 ligand localised on the surface of apoptotic cells activates the process of efferocytosis, the engulfment and the consumption of dead cells. The engagement of AXL receptors results in the activation of downstream pro-survival pathways, PI3K, MAPK, and NF-KB, which is required for the survival of professional phagocytes operating in toxic environments^12,15^. Correspondingly, pathological activation of the AXL/GAS6 pathway in cancer represents the acquired resistance mechanism to chemo-, radio- or immune therapies. Several small molecule inhibitors (AXLi) with various degrees of selectivity have been developed to combat the AXL/GAS6-mediated resistance. In vitro, AXL inhibitors, such as TP-0903 or R428 (BGB324), reversed EMT and restored drug sensitivity in several cancer types, including TNBC^16–20^.

EMT and MET programs are integrated into intracellular signaling networks, which affect most basic physiological cellular functions, including metabolism and signal transduction. In particular, and relevant to cancer therapy, EMT-TFs modulate reduction–oxidation (redox) signaling and cellular response to the production of reactive oxygen species (ROS). Redox signaling is mediated by electron transfer and contributes to various biological processes in normal and pathological.

conditions. The central players in redox pathways are ROS, predominantly produced in mitochondria as a side product during oxidative phosphorylation^21^. Excessive ROS levels induce oxidative stress that damages macromolecules, including DNA, proteins, and lipids, which may inflict programmed cell death. In apoptosis-resistant cancer cells, oxidative stress is a factor contributing to genomic instability and tumour heterogeneity.

The intracellular redox state depends on the equilibrium between ROS production and the activity of antioxidant defence, a complex system of enzymes and non-enzymatic molecules. Individual enzymatic antioxidants scavenge particular ROS species^21,22^. The superoxide dismutases (SODs) are localised in mitochondria or cytosol and convert superoxide anion to oxygen and hydrogen peroxide. Catalases decompose hydrogen peroxide into water and oxygen and are mainly located in peroxisomes. Other enzymes, such as glutathione reductase, glutathione peroxidases (GPXs), and glutathione S-transferases (GST), are implicated in antioxidant defence by glutathione (GSH). GSH, a Cysteine-containing tripeptide, neutralises ROS in the reduced state to form oxidised GSH which is then converted to a reduced form by glutathione reductase^22^.

In breast cancer, elevated components of antioxidant machinery correlate with higher stage and poor response to the treatment^23,24^. There are reports demonstrating that the level of antioxidant activity is higher in cells undergoing an EMT^25–27^. Therefore, we reasoned that EMT reversal might sensitise TNBC cells to ROS-inducing therapeutics. Here, we tested whether breast cancer cells' differentiation status impacts their sensitivity to a ROS-inducing antimalarial agent, artesunate (ART). ART is a water-soluble derivative of artemisinin, a compound extracted from the plant Artemisia annua. Intravenous administration of ART is approved by the World Health Organization to treat severe malaria. After intravenous administration, ART has a half-life of approximately 15 min with clearance and volume values of 2–3 l/kg/h and 0.1–0.3 l/kg, respectively^28^. Recently, ART has been repurposed for cancer therapy because of the encouraging results obtained in vitro. ART inhibited cell proliferation, cell invasion and induced apoptosis in cell lines derived from different cancer types^29–33^. In addition, ART demonstrated anticancer activity in breast and myeloid leukemia xenograft models^34,35^. Mechanistically, ART exerts cytotoxicity mainly by activating the endoperoxide bridge through ferrous iron (FeII) or heme, leading to ROS generation^32,36^. We found that reversing EMT cells via depletion of EMT-TF ZEB1 or using AXL inhibitor TP-0903 resulted in the sensitisation of TNBC cells to ART, although not via identical mechanisms.

## Results

### Cellular sensitivity to ART is reduced in mesenchymal breast cancer cell lines

**Figure 1:**
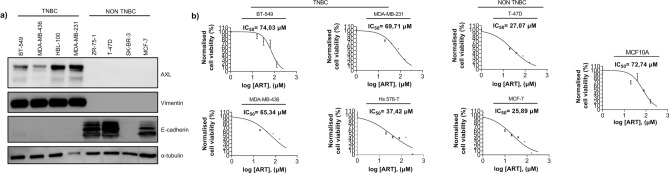
TNBC cell lines were more resistant to the treatment with ART. (**a**) Characterisation of breast cancer cell lines. Immunoblots show expression of mesenchymal proteins vimentin and AXL and epithelial marker E-cadherin in breast cancer cell lines. While TNBC-derived cell lines expressed AXL and vimentin, non-TNBC-derived cells expressed epithelial marker E-cadherin. In HER + cell line SK-BR3, E-cadherin-encoding CDH1 gene carries a large homozygous deletion^39^. Anti- Tubulin antibody was used to confirm equal loading; (**b**) MTS assay (BT-549) and MTT assay (all remaining cell lines) demonstrated that IC_50_ values for ART were higher in mesenchymal cells (between 37.42 μM and 74.03 μM) than in MCF-7 or T-47D cell lines (25.89 μM and 27.07 μM respectively). Note that MCF-10A cells displayed higher resistance to ART than most breast cancer cell lines. ART IC_50_ for MCF-7 and MDA-MB-231 are extracted from the MTT data on MCF- 7/ZEB1 (-) Dox and MDA-MB-231 siRNA cntrl presented in Fig. 2a. MTT results are expressed as mean ± SEM of six technical replicates (except for MCF-10A analysed in triplicate) while MTS results are expressed as mean ± SEM of three technical replicates. IC_50_ values were determined using MTT assay as described in Materials and Methods. In the immunoblots shown in figure a, the membranes were cut into fragments containing proteins of known molecular weights before incubating with the antibodies. This also applies to the blots presented in Figs. 2, 3d, 4a,b,e, 5b,c.

EMT programs drive resistance to most clinically-approved drugs via various mechanisms^11,37^. We tested whether the sensitivity of breast cancer cell lines to ART correlated with the differentiation status of the cells. For these experiments, we selected four mesenchymal (BT-549, MDA-MB-436, HBL-100, and MDA-MB-231) expressing RTK AXL and two epithelial AXL-negative cell lines (T-47D and MCF-7) (Fig. 1a). While all mesenchymal cell lines represented TNBC, MCF-7 and T- 47D belonged to the luminal A subtype^38^. We applied ART to the cell cultures at 0 to 256 μM (BT-549) and 0 to 320 μM concentrations (all other cell lines). Cells were treated for 72 h and IC_50_ values were determined. Two epithelial cell lines, T-47D and MCF-7, appeared more sensitive to ART than any TNBC-derived cell lines (Fig. 1b). Of note, non-tumourigenic human mammary epithelial cells MCF-10A exhibited resistance to the treatment with ART (IC_50_ value equal to 72.74 μM) (Fig. 1b).

### EMT protected breast cancer cells from ART-induced apoptosis

**Figure 2:**
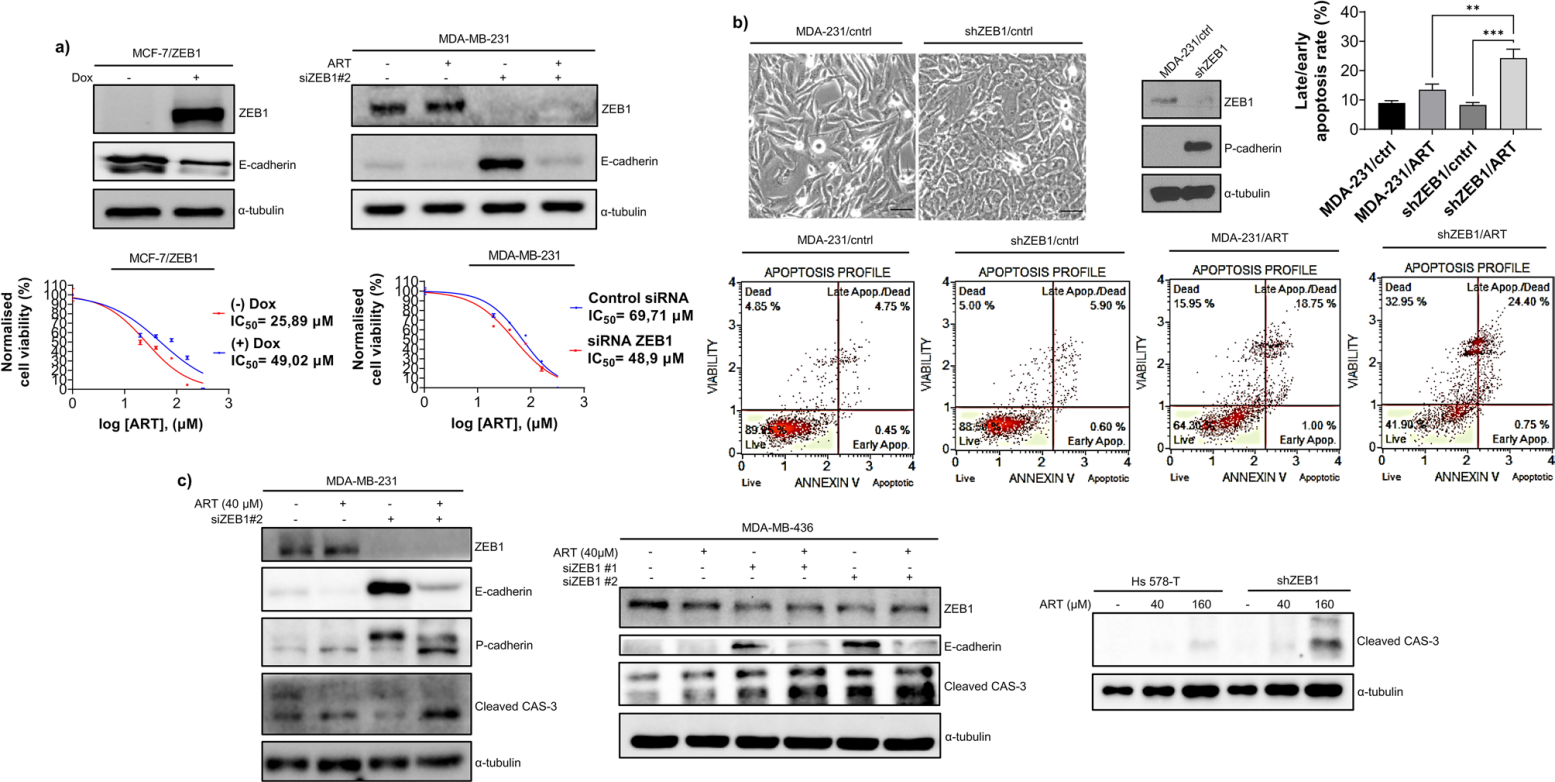
Differentiation status of breast cancer cells determined their response to ART treatment. (**a**) EMT was induced by ectopic expression of ZEB1 in MCF-7 cells (left panel) or reverted by ZEB1 depletion in MDA-231 cells (right panel). Western blots demonstrate that modulation ZEB1 expression affected E-cadherin levels. Whereas inducing EMT in MCF-7 cells increased viability of ART-treated cells, activating partial MET in MDA-231 cells by ZEB1 KD sensitized cells to ART. IC_50_ values were determined using MTT assay as described in Materials and Methods. MTT results are expressed as mean ± SEM of six technical replicates; (**b**) Reduced viability of epithelioid cells in response to ART associated with increased rate of apoptosis. shRNA-mediated depletion of ZEB1 in MDA-MB-231 cells led to a partial MET as evidenced by the analysis of cell morphology and reactivation of P-cadherin. ZEB1 depletion increases the proportion of Annexin V- positive cells treated with 160 μM ART for 48 h. Graph shows results of four independent measurements (mean ± SEM). A one-way Anova test followed by a post hoc Tukey’s multiple comparisons test were used to check significance among groups; *p < 0.05; ** p < 0.01; *** p < 0.001; (**c**) ZEB1 depletion in mesenchymal breast cancer cell lines resulted in the cleavage of caspase-3. ZEB1 KD was carried out by siRNA (in MDA-MB-231 or MDA-MB-436 cells) or using shRNA (in Hs 587-T cells) and expression of ZEB1 cleaved caspase-3 and cadherins was analysed by immunoblotting as indicated. Tubulin was used as a loading control.

As sensitivity to ART correlated with the differentiation state of the cells (Fig. 1), we aimed to test whether EMT protects cancer cells from ART-induced cytotoxicity. To this end, we employed two cell models, MCF-7 cells with Doxycycline-inducible expression of ZEB1^40^ and MDA-MB-231 cells in which ZEB1 KD induced partial MET. As expected, modulating ZEB1 expression levels affected the EMT status of the cells (Fig. 2–c). MTT assay demonstrated that ZEB1-induced EMT strongly reduced cytotoxic effects of ART in MCF-7 cells. Accordingly, ZEB1 depletion significantly sensitised MDA-MB-231 cells to ART treatment (Fig. 2b). ART was capable of activating apoptosis in cancer cells by inducing mitochondrial membrane permeabilization and cytochrome c release leading to the activation of caspases^41^. To test if the reversal of EMT potentiated ART-induced apoptosis, we employed MDA-MB-231 cells with shRNA-mediated ZEB1 depletion (MDA-231/shZEB1). ZEB1 KD in these cells resulted in the transition towards epithelial morphology and reactivation of P-cadherin expression (Fig. 2b). Notably, depletion of ZEB1 significantly increased rates of Annexin V-positive cells in response to the treatment with ART (Fig. 2b). Consistent with these observations, we found that ZEB1 KD potentiated activation of caspase-3 by ART in different mesenchymal breast cancer cell lines. Indeed, reduction of the levels of ZEB1 by siRNA enhanced ART-induced cleavage of caspase 3 in MDA-MB-231 and MDA-MB-436 cells (Fig. 2c). Likewise, shRNA-mediated ZEB1 KD in Hs 578-T cells potentiated ART-induced caspase- 3 cleavage (Fig. 2c).

### ZEB1 influenced the extent of ART-generated ROS and DNA damage

**Figure 3:**
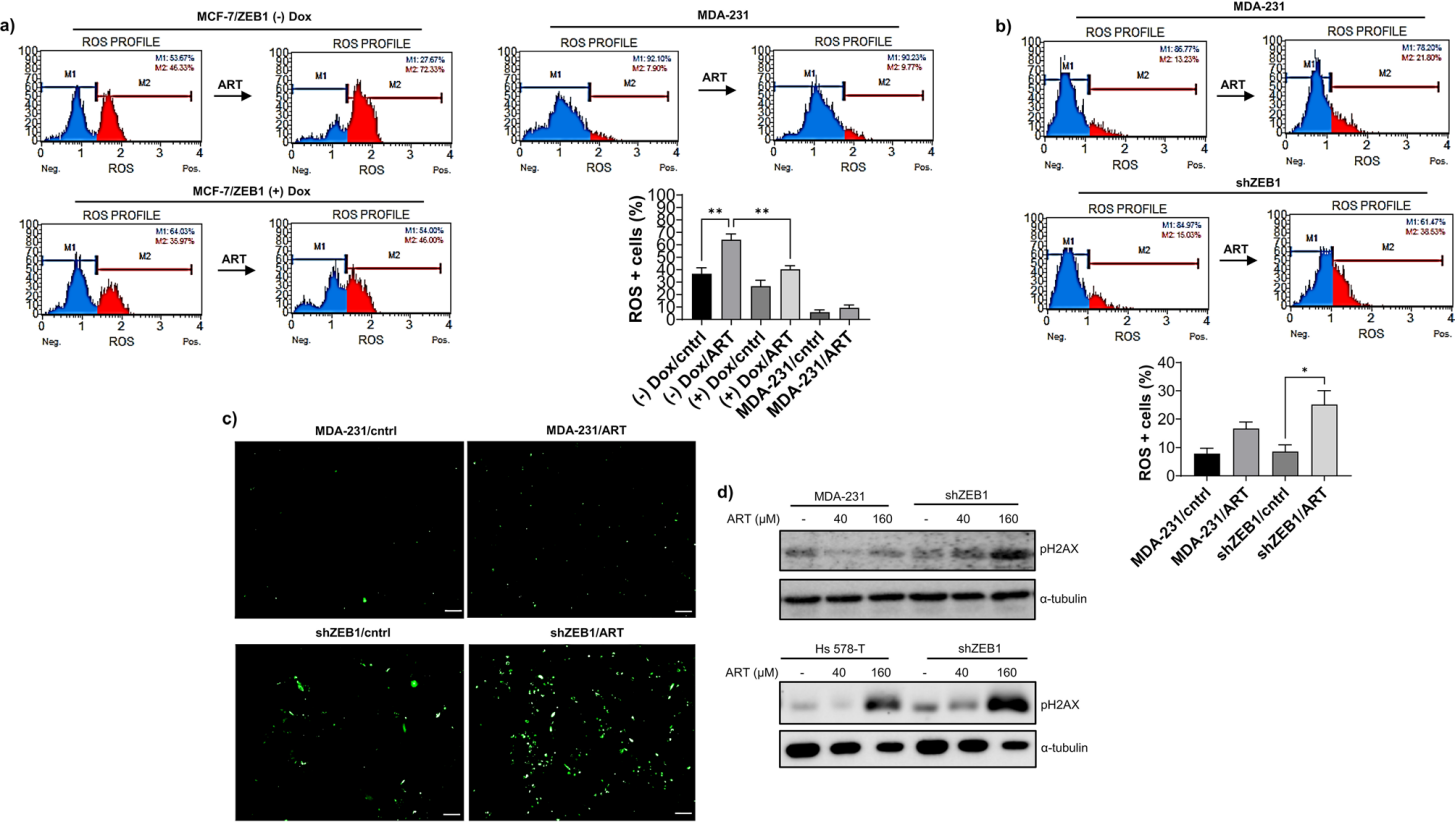
ZEB1 expression impact on ART-generated ROS levels and DNA damage. (**a**) ZEB1-induced EMT reduced the proportion of ROS-positive cells induced by ART treatment. Treatment of MDA-MB-231 cells with ART in these experiments illustrated low levels of ROS generation in ART- resistant cells; (**b**) ZEB1 depletion in MDA-MB-231 cells increased the ROS production in ART-treated or untreated cells. (**a, b**) Proportion of ROS-positive cells was determined using Muse Oxidative assay which detects superoxide anion O_2_-. Blue and red peaks reflect proportions of ROS- negative and ROS-containing cells, respectively. The experiments demonstrated that ZEB1 expression reduced proportion of superoxide positive cells in response to ART treatment. Graphs show the results expressed as mean ± SEM of three (**a**) and four (**b**) independent experiments. A one- way Anova test followed by a post hoc Tukey’s multiple comparisons test were used to check significance among groups; * p < 0.05; ** p < 0.01; *** p < 0.001; (**c**) MDA-MB-231 control or ZEB1 KD cells were treated with ART or left untreated. The amount of cells with high general oxidant levels was assessed using the fluorescent reporter CM-H2DCFDA; scale bar 100 μm; (**d**) ZEB1 protected DNA from ART-induced DNA damage. Control or ZEB1-depleted MDA-MB-231 or Hs 578-T cells were treated with the indicated concentrations of ART and the level of DNA damage evaluated using an anti-gH2AX antibody.

Cytotoxic effects of ART are associated with the generation of ROS in malaria parasites and cancer cells^36^. Therefore, we reasoned that EMT status affects sensitivity to ART by modulating ROS generation. We tested this hypothesis by employing Muse Oxidative assay to measure the effect of EMT on the generation of superoxide-positive cells in response to ART. In agreement with our hypothesis, mesenchymal MDA-MB-231 cells untreated or treated with 80 μM ART displayed much less superoxide positivity than epithelial MCF-7 cells. Induction of ZEB1 expression in MCF-7 cells significantly reduced superoxide generation (Fig. 3a). To test the impact of ZEB1 on ROS generation in mesenchymal cells, MDA-MB-231/control and MDA-MB-231/shZEB1 cells were incubated with 160 μM ART for 48 h. We observed a significant increase in the levels of superoxide in MDA- MB- 231/shZEB1 cells in response to ART treatment. In contrast, the difference in superoxide levels was not significant in MDA-MB-231 control cells mock-treated or treated with ART (Fig. 3b). Similarly, total ROS level detected with the CM-H2DCFDA reagent was higher in ART-treated cells with the depleted ZEB1 expression than in control cells (Fig. 3c). Consistent with this observation, we detected increased histone H2AX phosphorylation in ART-treated MDA-MB-231/shZEB1. Likewise, ZEB1 KD stimulated phosphorylation of H2AX in response to ART treatment in Hs 587- T cells (Fig. 3d). Together, these data indicated that the presence of ZEB1 reduced the generation of ROS and the subsequent formation of phosphorylated H2AX that is an early marker of cellular response to DNA damage.

### AXL inhibitor TP-0903 affected EMT status and potentiated ART- induced apoptosis

**Figure 4:**
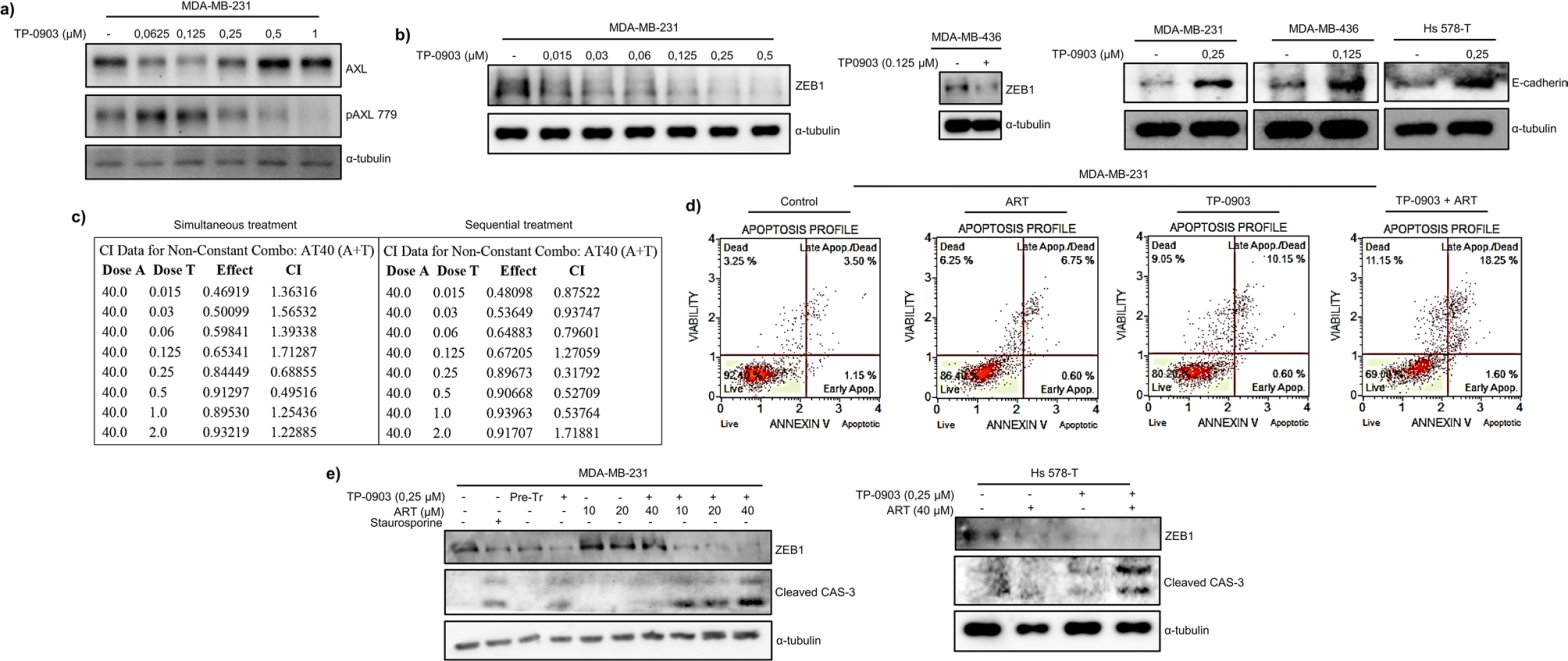
AXL inhibitor TP-0903 promoted MET and potentiated pro-apoptotic effects of ART. (**a**) TP-0903 effectively inhibited AXL phosphorylation. Total and phosphorylated AXL protein was detected by immunoblotting in cells treated with the indicated concentration of TP-0903; (**b**) Prolonged treatment with TP-0903 led to the partial restoration of epithelial phenotypes in TNBC cells. MDA-MB-231, Hs 578-T or MDA-MB-436 cells were maintained with indicated concentrations of the inhibitor for 48 h and expression of ZEB1 or E-cadherin analyzed in Western blotting; (**c**) A representative CompuSyn report after simultaneous/sequential treatment of MDA-MB-231 cells with TP-0903 at different concentrations plus ART 40 μM for 96 h. The number of CIs values showing synergism was higher in the sequential treatment. Dose A = ART; Dose T = TP-0903; Effect = growth inhibition; CI = Combination Index. CI < 1 indicates synergism. Effect and CI were calculated after performing the MTT assay on four technical replicates; (**d**) MDA-MB-231 cells were cultured with or without TP-0903 0.25 μM and ART 40 μM, and apoptosis was analysed using fluorescent annexin V apoptosis assay; (**e**) Caspase 3 cleavage was tested in ART and TP-0903-treated cells by Western blotting with a cleaved caspase-3 specific antibody.

Our data indicated that induction of MET via ZEB1 KD sensitised TNBC cells to ART-induced apoptosis via a mechanism likely involving ROS. As ZEB1 is pharmacologically undruggable, we thought to employ a compound that is a) effective in reverting EMT and b) is approved for use in the clinic. RTK AXL was expressed in TNBC cell lines (Fig. 1a), it is required for the maintenance of mesenchymal phenotype in vitro and in vivo^13,42^ and several small molecule AXL inhibitors are currently being investigated in clinical settings. In particular, AXL inhibitor Dubermatinib (TP-0903) has shown promise in a clinical trial investigating AML patients’ response to combination treatment with decitabine^43^. Thus, we tested whether TP-0903 cooperated with ART in inducing cytotoxicity. 24-h treatment of MDA-MB-231 cells with 1 μM of the inhibitor reduced AXL phosphorylation on tyrosine-779 (Fig. 4a). Consistent with data in the literature^13,42^, prolonged treatment with AXL inhibitors led to the partial restoration of epithelial morphology in mesenchymal breast cancer cells (not shown), repression of ZEB1 and reactivation of E-cadherin expression (Fig. 4b). Next, we aimed to optimise the conditions of the combined treatment of breast cancer cells with TP-0903 and ART (i.e., conditions when adding both compounds result in maximal toxicity). MDA-MB-231 cells were exposed either simultaneously or sequentially to TP-0903 and ART. In a sequential treatment scheme, cells were pre-incubated with TP-0903 for 24 h followed by the treatment with TP-0903 plus ART for a further 72 h. The combination Index (CI) was determined after MTT assay as growth inhibition rate and reflected compound interactions (Fig. 4c). A sequential combination had a higher number of CIs lower than 1 (synergism) than in the course of simultaneous treatment. In addition, in a sequential treatment, TP-0903 synergised with lower ART concentrations (2.5 μM–20 μM) compared to a simultaneous treatment, in which TP-0903 synergised with higher ART concentrations (Compusyn Report—Supporting information file 2). As TP-0903 at 0.25 μM concentration synergised with all ART concentrations (Compusyn Report—Supporting information file 2) and significantly suppressed ZEB1 (Fig. 4b), this condition was used for further experiments. In line with the reduced cell viability (Fig. 4c), sequential treatment of MDA-MB-231 cells with TP-0903 and ART increased the proportion of apoptotic cells compared to the single-agent treatment (Fig. 4d) and stimulated cleavage of caspase-3 (Fig. 4e). Similar effect of TP-0903 on caspase-3 activation was observed also in Hs 578-T cells (Fig. 4e).

### TP-0903 and ART synergistically increased total ROS and DNA damage and reduced expression of key antioxidants

**Figure 5:**
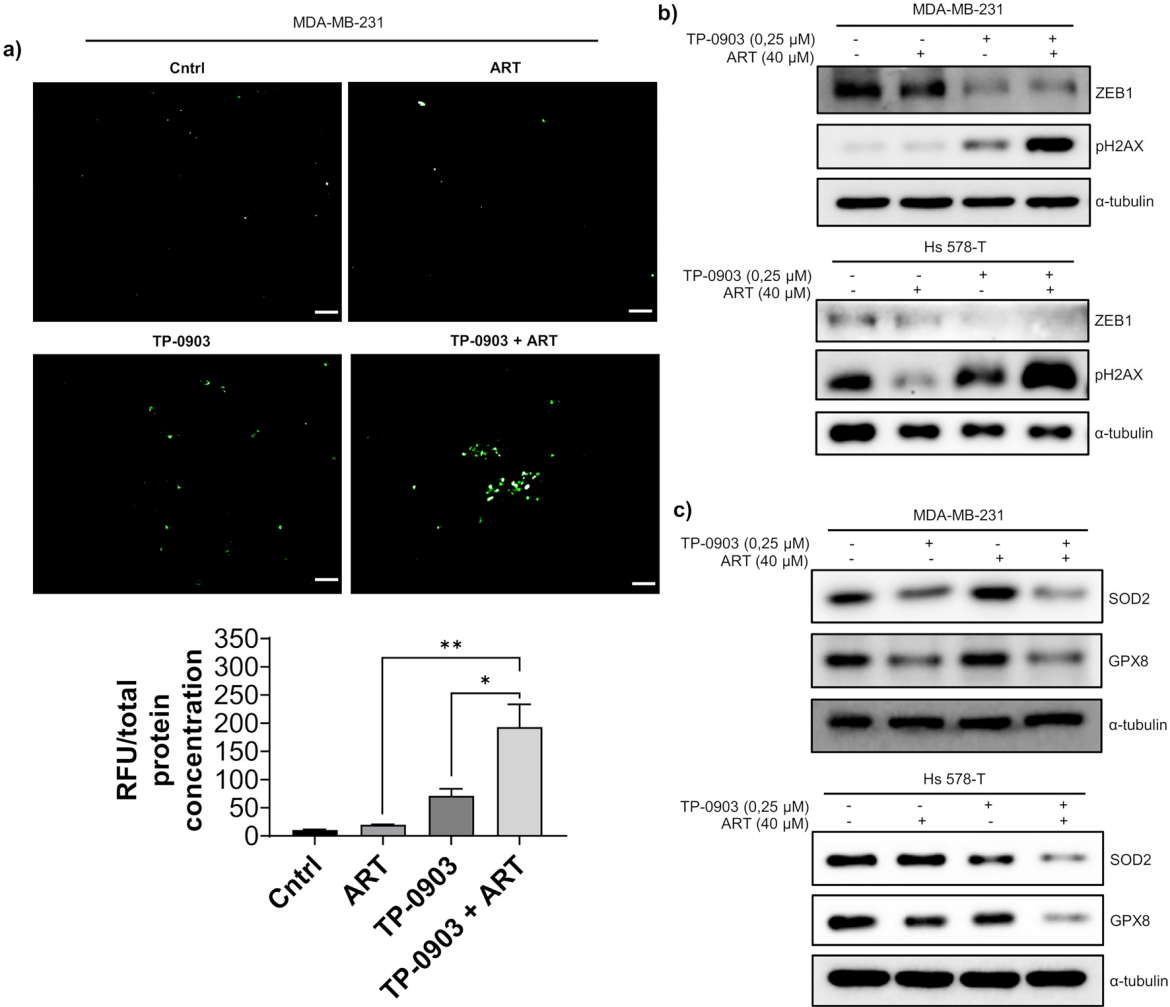
Combined treatment with ART and TP-0903 stimulated total ROS production, gH2AX expression and repressed SOD2 and GPX8. (**a**) MDA-MB-231 cells were treated with ART and TP-0903 as indicated or left untreated. Fluorescence intensity was calculated by the ImageJ software. Results are expressed as mean ± SEM of three different microscopic fields. One-way Anova test followed by a post hoc Tukey’s multiple comparisons test were used to analyse significance between control and treatment groups. * p < 0.05; ** p < 0.01; *** p < 0.001; scale bar 100 μm; (**b, c**) Expression of indicated proteins was analysed by Western blotting as shown.

ZEB1 KD potentiated the effect of ART on ROS generation (Fig. 3), and TP-0903 treatment reduced ZEB1 expression (Fig. 4b). These results suggested that ART-induced production of ROS can be enhanced by TP-0903. To test this, MDA-MB-231 cells were pre-incubated with 0.25 μM TP-0903 for 24 h. Then combination treatment with TP-0903 and 160 μM ART was applied for further 24 h. According to the CM-DCFH DA test, after normalization to total protein concentration, ART treatment in combination with TP-0903 induced higher total ROS levels as compared to the control and single-agent treatments (Fig. 5a). Consistent with these data, we observed strong cumulative effects of ART and TP-0903 on histone H2AX phosphorylation (Fig. 5b). We proposed that EMT- TFs, and in particular ZEB1 may decrease ROS levels by increasing expression of antioxidant molecules. Indeed, the inspection of RNAseq data analysing the expression of ZEB1-regulated genes in MCF7/ZEB1 cells revealed a number of genes central to ROS scavenging (not shown). As confirmed by qPCR, two of these genes, *GPX8* and *SOD2*, were significantly upregulated by ZEB1 (Fig. S1). In accordance with these observations, treatment with TP-0903 reduced GPX8 and SOD2 protein levels (Fig. 5c and Fig. S2) suggesting that TP-0903-mediated reduction in the levels of these antioxidants could contribute to the cytotoxic effect of ART.

## Discussion

TNBC is an aggressive form of breast cancer associated with poor survival. Currently, there are no targeted treatment schemes for patients with this cancer type. Two out of five TNBC molecular subtypes, ML (Mesenchymal-Like) and MSL (Mesenchymal/Stem-Like), exhibit EMT, a cellular program that confers drug resistance and metastatic disease. Drug repurposing is considered a strategic approach to developing better treatment schemes to improve TNBC patients’ survival^44^. In this context, the antimalarial agent artemisinin derivative ART was considered a prospective anti- TNBC candidate compound. ART in vivo is converted to the active metabolite dihydroartemisinin (DHA) suggesting that ART acts primarily via DHA^45^. However, rather than DHA, ART is the most clinically relevant artemisinin-derived compound. Indeed, ART has higher water solubility making it suitable for intravenous administration in patients with severe malaria^28^ and results from clinical trials have shown that ART is more favourable than other artemisinins in malaria treatment^46^. Subsequently, the Food and Drug Administration has approved ART as the first-line agent for the management of severe malaria at a fixed recommended dose. Moreover, good tolerability to intravenous administration of ART has been shown in clinical trials involving patients with high-stage malignancies, including metastatic breast cancer^47–49^. The reported anti-cancer activity of ART is context- dependent and caused by the generation of ROS, subsequent genotoxic stress, damage of organelles and apoptotic or ferroptotic cell death. In TNBC cells, ART activated mitochondrial apoptosis^50^ but prolonged treatment with ART of MDA-MB-231 cells caused resistance^51^.

In this study, we report that the differentiation status is a determinant of the sensitivity of breast cancer cell lines to ART with mesenchymal TNBC-derived cell lines displaying enhanced resistance (Fig. 1b). Consistent with the previous report^50^, non-tumourigenic mammary epithelial MCF-10A cells were resistant to ART treatment (Fig. 1b). Inducing EMT by overexpression of EMT-TF ZEB1 in MCF-7 cells (Fig. 2a) or reverting EMT by ZEB1 depletion in mesenchymal carcinoma cells (Fig. 2b,c), respectively reduced or enhanced ART-induced apoptotic response. As targeting ZEB1 by pharmacological means is not possible, we analysed cooperation between ART and clinically approved inhibitors of RTK AXL, which are known to induce epithelisation. We found that a small molecule AXL inhibitor TP-0903 initiated MET (Fig. 4b) and synergistically enhanced the cytotoxic effect of ART in TNBC-derived cell lines (Fig. 4c–e). Apoptosis induced by the combined treatment with ART and TP-0903 was accompanied by increased levels of ROS (Fig. 5a) and DNA damage (Fig. 5b), as evidenced by the enhanced phosphorylation of histone H2AX on serine 139. Consistent with these data, these treatment conditions reduced the expression of key antioxidant enzymes, SOD2 and GPX8 (Fig. 5c and Supplementary Figure S2), which could explain the increase in ROS levels and elevated cytotoxicity. In line with these observations, ectopic expression of ZEB1 strongly induced transcription of both genes in MCF-7 cells (Supplementary Figure S1). According to the hierarchical clustering analysis of 52 breast cancer cell lines, *SOD2* and *GPX8* genes were part of the mesenchymal gene signature and co-clustered with *ZEB1*, *VIM*, and *SNAI2* (Supplementary Figure S3). Taken together, these observations may indicate that *SOD2* and *GPX8* genes are targets of ZEB1 in TNBC cells.

However, contrary to our expectations, stable depletion of ZEB1 in MDA-MB-231 or Hs 578-T cells did not decrease SOD2 or GPX8 protein levels (not shown) indicating that TP-0903 regulates SOD2 and GPX8 in these cell lines via a different, ZEB1-independent mechanism. In addition to ZEB1, expression of other EMT-TFs, such as SNAIL and SLUG, was reduced in TNBC cells treated with TP-0903 (Supplementary Figure S4). It has been well established that the functions of EMT-TFs in both normal physiological conditions and cancer are not redundant^7^. Therefore, one might speculate that SNAIL, SLUG, or probably other EMT-TFs have a specific role in maintaining elevated expression of antioxidant genes in mesenchymal TNBC cells.

MET induction by ZEB1 KD or using TP-0903 may increase ROS levels via mechanisms that are independent of the prevention of ROS scavenging but through stimulation of their production.

Metabolic reprogramming is one of the hallmarks of EMT^52,53^. A reversible switching from ROS-generating oxidative phosphorylation (OXPHOS) to anaerobic glycolysis occurs during EMT, and critical components of the glycolytic pathway are directly regulated by EMT-TFs^54,55^. Reversing this switching via MET programs may reactivate OXPHOS in TNBC cells and subsequently enhance ROS levels. Whichever mechanisms leading to ROS elevation are, TP-0903-mediated increase in ROS sensitises cells to drugs, such as ART, which can further stimulate their production. Thus, the combined effects of TP-0903 and ART increased ROS levels over the cytotoxic threshold leading to apoptosis.

Insufficient DNA repair may represent another factor contributing to the sensitisation of TNBC cells to ART. Various DNA repair pathways are stimulated by EMT-TFs, including ZEB proteins^56,57^, and, therefore, epithelisation induced by ZEB1 depletion or by TP-0903 may compromise the repair of DNA lesions, subsequently inducing cell death.

TP-0903, initially developed as an AXL inhibitor, was later found to be active against several other protein kinases, including ABL1 and JAKs^58,59^. We found that in TNBC cells, this inhibitor was much more capable of inducing MET than AXL KD or other small molecule AXL inhibitors, namely R428^60^ or LDC1267^61^ (not shown).

Approaches proposing the use of anti-EMT therapy as an adjunct to the treatments effective against epithelial tumours have been comprehensively discussed in the literature^11,62^. Our study investigates the use of TP-0903 (EMT reversal) and ART (ROS inducer) and lays within the framework of this strategy. The selection of drug concentrations for our experiments was directed by the information on the dosage used in clinical settings. According to the World Health Organization recommendations, the dosage for the IV injection of ART is 2.4 mg/Kg/day for patients with severe malaria^63^, a dose corresponding to the 33,6 mg/ml or 87,4 μM concentration in the blood. The 200 mg/d oral dose for ART was recommended for treating breast cancer patients (clinical trial ARCTIC M33/2,^64^), corresponding to 40 mg/L or 104 μM. As a part of clinical trial NCT03013998, acute myeloid leukemia (AML) patients were treated with 25 mg/day TP-0903 or 9.6 μM^65^. Thus, in most of our experiments, we used ART and TP-0903 concentrations lower than those applied in clinical trials. Either agent, ART (clinical trial ARCTIC M33/2) and TP-0903 (clinical trial NCT03013998) display satisfactory tolerability when administrated as single-agent therapy. However, as we show here, TP-0903 significantly increases ART-induced ROS levels. Therefore, one can anticipate that the toxicity will be enhanced when both compounds are combined. As proliferating cells are more sensitive to oxidative stress, combination treatment with ART and TP-0903 may affect highly proliferative tissues, such as the gut epithelium or the skin. It is, therefore, essential to use mouse models to determine the safe and effective schedule and dose levels of TP-0903/ART to provide conclusive pre-clinical data on TP-0903/ART toxicity and efficacy for TNBC patients.

## Conclusion

Our results demonstrate a synergism between ART and induction of MET in TNBC cells. These observations justify further preclinical research on the evaluation of combining ART with MET-inducing compound TP-0903 to treat women with TNBC.

## Materials and methods

### Cell culture, ZEB1 overexpression and ZEB1 silencing

Commercial breast cancer cell lines MCF-7, T-47D, ZR-75–1, SK-BR3, MDA-MB-231, MDA-MB- 436, BT-549, HBL-100 and the non-tumorigenic breast cell line MCF-10A were cultured in complete Dulbecco’s modified Eagle’s medium (DMEM) supplemented with 10% foetal bovine serum and 1% penicillin– streptomycin (Capricorn Scientific GmbH, Ebsdorfergrund, Germany) at 37 °C and 5% CO2. Cells with the doxycycline (DOX)-inducible ZEB1 expression, MCF-7/ZEB1^40^, were maintained in the presence of absence of 1 μg/ml DOX for 72 h prior the experiments were carried out. To generate MDA-231 and Hs 578-T cells with the stable ZEB1 knockdown, cells were infected with the pLKO.1- PURO lentiviral vectors (Sigma-Aldrich, St. Louis, MO, USA) expressing ZEB1-targeting short hairpin RNA (shRNA) or control shRNA. Selection of cells expressing shRNAs was performed in 0.5 μg/ml puromycin-containing DMEM for 7–10 days. For transient ZEB1 depletion, short interfering RNA (siRNA) was purchased from Thermo Fisher Scientific (Waltham, MA, USA) (siRNA#1, and #2, Catalogue numbers 229970 and 229,971). siRNA transfection was performed using LTX Lipofectamine with Plus reagent (Thermo Fisher Scientific).

### Western blotting

Cells grown at 70%-80% of confluence were harvested in Laemmli buffer, heated at 95 °C and sonicated. Pierce BCA protein assay (Thermo Fisher Scientific) was utilised to measure protein concentrations. Samples were separated on 8% or 15% SDS polyacrylamide gels and transferred onto a polyvinylidene fluoride membrane (Sigma-Aldrich). Then, 5% non fat dry milk was used to block membranes for 60 min before incubation with primary and secondary antibodies at 25 °C for 1 h. Before incubating with the antibodies, the membranes were cut into fragments containing proteins of known molecular weights and protein expression was detected by Super Signal HRP chemiluminescent substrates (Thermo Fischer Scientific). The images were taken by the ChemiDoc Imaging System (Bio-Rad Laboratories). Where the edges of membranes are not visible, this is a result of shorter exposure times. Shorter exposure times allowed to avoid signal saturation at longer exposures. Original images are presented in Supporting Information file 1. The following primary antibodies were used in the study: anti-ZEB1 (Santa Cruz Biotechnology, Dallas, TX); anti- vimentin, anti-E-cadherin and anti-P-cadherin (BD Biosciences, San Diego, CA); anti-SLUG, anti- SNAIL, anti-cleaved caspases (Cell signalling Technology, Danvers, MA, USA); anti-AXL (total), anti-phospho-AXL (Y779) (R&D Systems, Minneapolis, MN, USA); anti-gH2aX and anti-Tubulin (Sigma-Aldrich).

### Quantitative PCR

Total RNA was purified from subconfluent cell cultures by the RNeasy Mini RNA isolation kit (Qiagen, Germantown, MD, USA). 1 μg of total RNA was used for cDNA synthesis with iScript Select first strand cDNA Synthesis Kit using oligo(dT)20 primers (Bio-Rad Laboratories, Watford, UK). For quantitative PCR (qPCR), triplicate experiments were carried out using SYBR Green Master Mix (BioRad) in 40 cycles with the annealing/extension temperature 60/72 °C. For data normalisation, amplification of a housekeeping gene, GAPDH, was used. To control for non-specific amplification, dissociation curves were examine, and PCR products analysed in agarose gels. Data were analysed by conventional ΔΔCT method; PCR primer sequences are shown in Table 1.

**Table 1:** Nucleotide sequences of primers for amplification of *SOD2, GPX8,* and *GAPDH* genes.

Target gene	Forward	Reverse
*SOD2*	AGCACCAGCACTAGCAAGCATG	CCGTAGTCGTAGGGCAGGTCG
*GPX8*	GCCTCTTGCAGCTTACCCGC	GTTGGCAGTCACTGGCCAGC
*GAPDH*	GTCTCCTCTGACTTCAACAGCG	ACCACCCTGTTGCTGTAGCCAA

### Cell viability assays

Cell viability was analysed according to the manufacturer’s protocols for MTT (3-(4,5- dimethylthiazol-2-yl)-2,5-diphenyltetrazolium bromide) (Sigma Aldrich) or MTS (3-(4,5- dimethylthiazol-2-yl)-5-(3-carboxymethoxyphenyl)-2-(4-sulfophenyl)-2H-tetrazolium) (Abcam, Waltham, MA, USA) assays. In brief, cells were seeded in 96-well plates at 70%-80% confluence and treated or mock-treated with various concentrations of ART (Sigma-Aldrich), TP-0903 (MedChemExpress, Monmouth, NJ, USA), or their different combinations as described in the Results section. After three days of incubation, the medium was removed. MTT or MTS reagents were added and left at 37 °C to form crystals (MTT assay) or coloured formazan dye (MTS assay). For the MTT assay, cells were solubilized in DMSO, and absorbance values were determined at 570 nm. MTS assay did not require solubilisation, and absorbance values were measured at 490 nm. After subtracting blank absorbance, cell viability was defined as a ratio of absorbance in drug-treated wells to that in mock-treated controls. CompuSyn software^66^ was used to calculate the combination index (CI), determining the type of drug interaction.

### Analysis of apoptosis

In order to determine the number of apoptotic cells after drug treatment, Muse Annexin V & Dead Cell Assay (Sigma-Aldrich) was performed following the manufacturer’s protocol. 200,000 cells/well were seeded in 6-well plates. After cell cultures reached 70%–80% of confluence, they were treated with the drugs as described in the Results section. Adherent cells and cells in the suspension were harvested and diluted in PBS. 100 μl of cell suspension was mixed with 100 μl Muse™ Annexin V & Dead Cell Reagent, incubated for 20 min, and their apoptotic profile was analysed through Muse™ Cell Analyzer (Merck Millipore, Darmstadt, Germany).

### Detection of reactive oxygen species (ROS) by flow cytometry

We employed Muse Oxidative stress kit for the detection of superoxide anion radicals in cells treated or mock-treated with ART or TP-0903. In brief, cells were seeded in six-well plates (2.5 * 105/well), treated accordingly and harvested. Cells were diluted, mixed with the assay reagent, incubated for 30 min at 37 °C, and flow cytometric analysis of ROS accumulation in cells at 12 h was performed using the Muse™ Cell Analyzer.

### Detection of hydrogen peroxide using CM-H2DCFDA assay

CM-H2DCFDA (6-chloromethyl-2',7'-dichlorodihydrofluorescein diacetate, acetyl ester) reagent (Thermo Fisher Scientific) was employed for hydrogen peroxide detection. Cells were seeded in six-well plates (2.5 * 105/well) containing coverslips, treated as described in the Results section, washed with the medium and then with PBS (twice). Next, coverslips were incubated with 8 μM CM- H2DCFHDA probe for 30 min at 37 °C, washed with PBS and mounted. Slides were photographed using the EVOS M5000 Imaging System.

### Hierarchical cluster analysis

The datasets were downloaded from the Expression Atlas – EMBL- EBI database and analysed by R v.4.2.3 programme in the RStudio v2023.03.0 Build 386 environment. Packages used were pheatmap and corrplot.

### Statistical analysis

Results were analysed by unpaired t-test and one way ANOVA test. p-values less than 0.05 implied that the differences among groups were significant. Statistical analysis was conducted by GraphPad Prism 9.0.0.

### Supplementary Information

Supplementary Figures.

Supplementary Information 1.

Supplementary Information 2.

## Data Availability

The data produced are available from the corresponding authors on request.
