# Supplementary material for: Mesenchymal–epithelial transition and AXL inhibitor TP-0903 sensitise triple-negative breast cancer cells to the antimalarial compound, artesunate
Source: Sci Rep. 2024 Jan 3;14:425. doi: 10.1038/s41598-023-50710-3 (PMC10764797; doi:10.1038/s41598-023-50710-3)
Supplement: Supplementary file 1 — Supplementary Figures. [file 41598_2023_50710_MOESM1_ESM.pdf]

**Title: Mesenchymal-epithelial transition and AXL inhibitor TP-0903 sensitise triple-negative breast cancer cells to the antimalarial compound, artesunate.**

**Mirko Terragno, Anastassiya Vetrova, Oleg Semenov, A. Emre Sayan, Marina Kriajevska, Eugene Tulchinsky**

**Supplementary Figures**

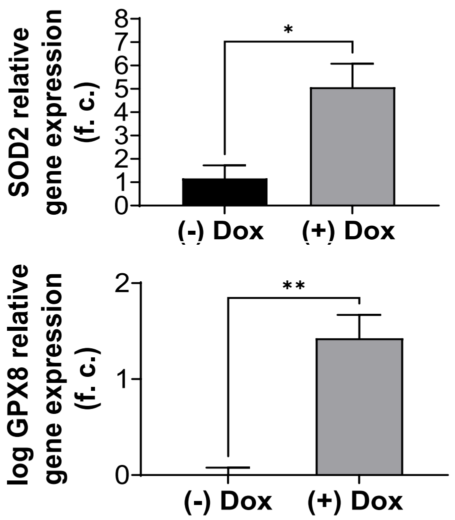


**Figure S1.** qPCR analysis of SOD2 and GPX8 gene expression in MCF7/ZEB1 cells maintained with or without DOX for 72 hours. Results are expressed as mean ± SEM of a technical triplicate. The unpaired t-test was performed; *p < 0.05; **p < 0.01; ***p < 0.001.

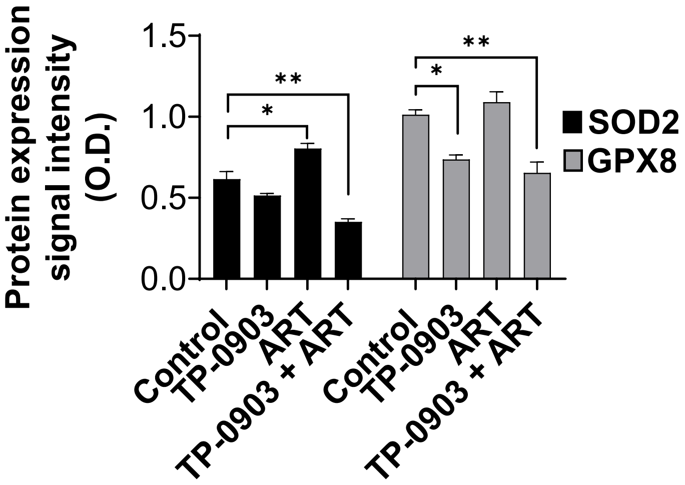


**Figure S2.** Analysis of SOD2 and GPX8 protein levels in MDA-MB-231 cells treated with TP-0903 and ART as single agents or in combination. Protein expression was analysed by Western blotting and band intensity measured using ImageJ software. Results are expressed as mean ± SEM of three independent experiments. A one-way Anova test followed by a post hoc Tukey's test were used to test significance between control and treatment groups; *p < 0.05; **p < 0.01; ***p < 0.001.

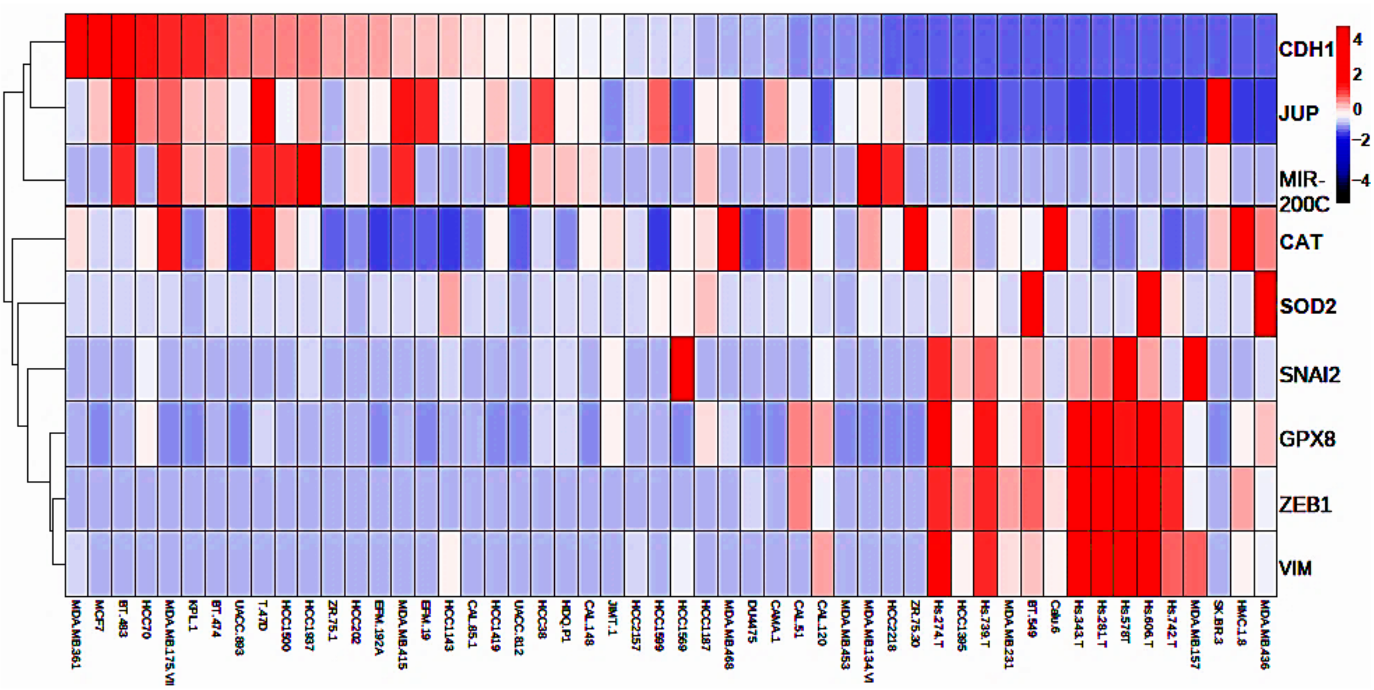


**Figure S3.** The heatmap of EMT markers and genes coding for antioxidant proteins expressed in breast cancer cell lines. Expression data were obtained from EMBL-EBI database and hierarchical cluster analysis was performed.

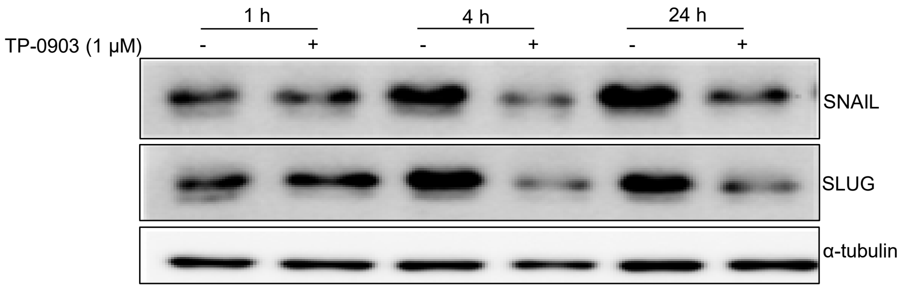


**Figure S4.** Western blot analysis of SNAIL family members, SNAIL and SLUG expressed in MDA-MB-231 treated or mock-treated with TP-0903 for the indicated time periods.
