# Supplementary material for: Mesenchymal–epithelial transition and AXL inhibitor TP-0903 sensitise triple-negative breast cancer cells to the antimalarial compound, artesunate
Source: Sci Rep. 2024 Jan 3;14:425. doi: 10.1038/s41598-023-50710-3 (PMC10764797; doi:10.1038/s41598-023-50710-3)
Supplement: Supplementary file 2 — Supplementary Information 1. [file 41598_2023_50710_MOESM2_ESM.pdf]

Title: Mesenchymal-epithelial transition and AXL inhibitor TP-0903 sensitise triple-negative breast cancer cells to the antimalarial compound, artesunate.

Mirko Terragno, Anastassiya Vetrova, Oleg Semenov, A. Emre Sayan, Marina Kriajevska, Eugene Tulchinsky

Supporting information 1 file – Original Western Blot files

Membranes marked by a black rectangle are shown in the manuscript

Original Western Blot files for Figure 1A

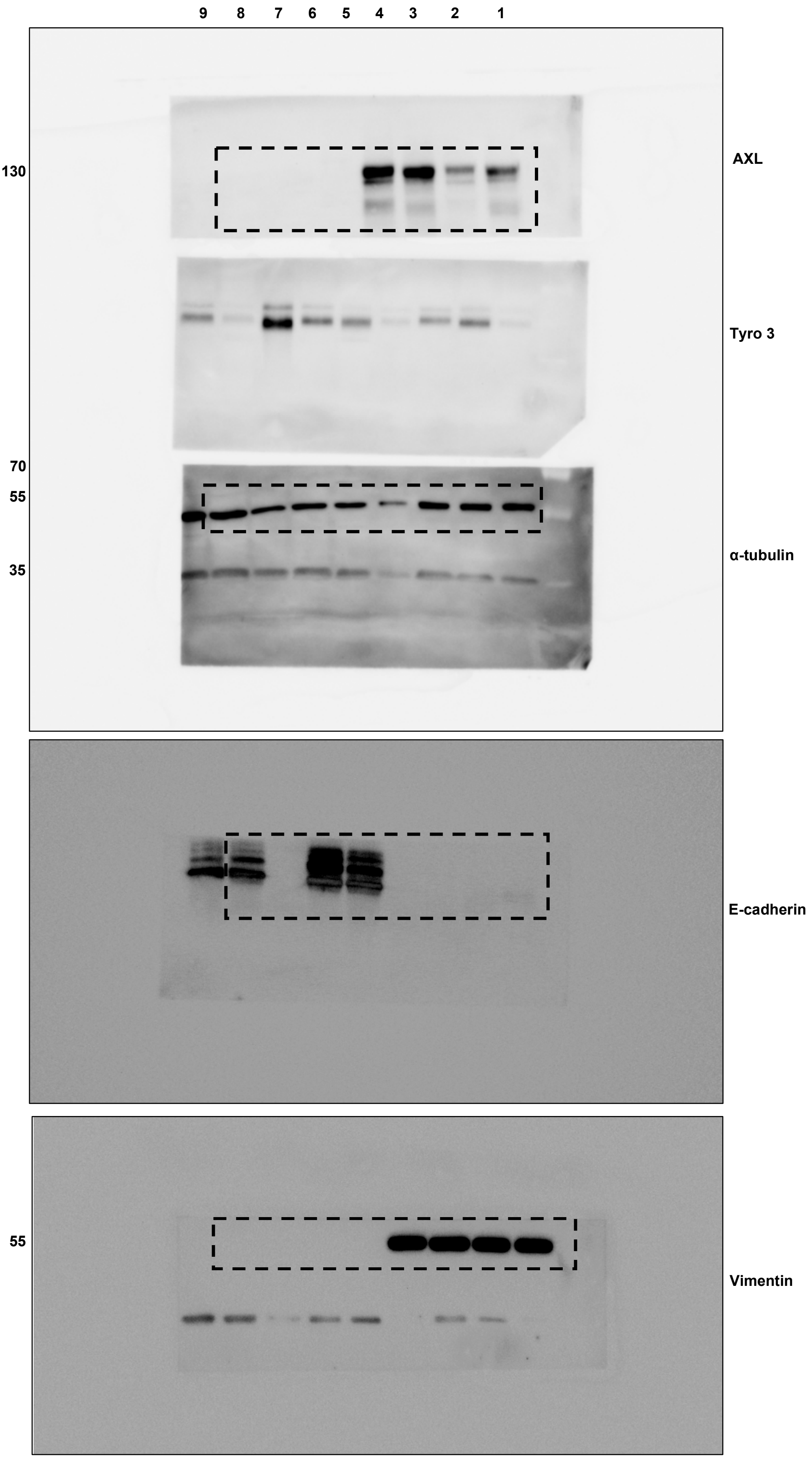

1. BT-549
2. MDA-MB-436
3. HBL-100
4. MDA-MB-231
5. ZR-75-1
6. T-47D
7. SK-BR-3
8. MCF-7
9. MDA-MB-468

Original Western Blot files for Figure 2A

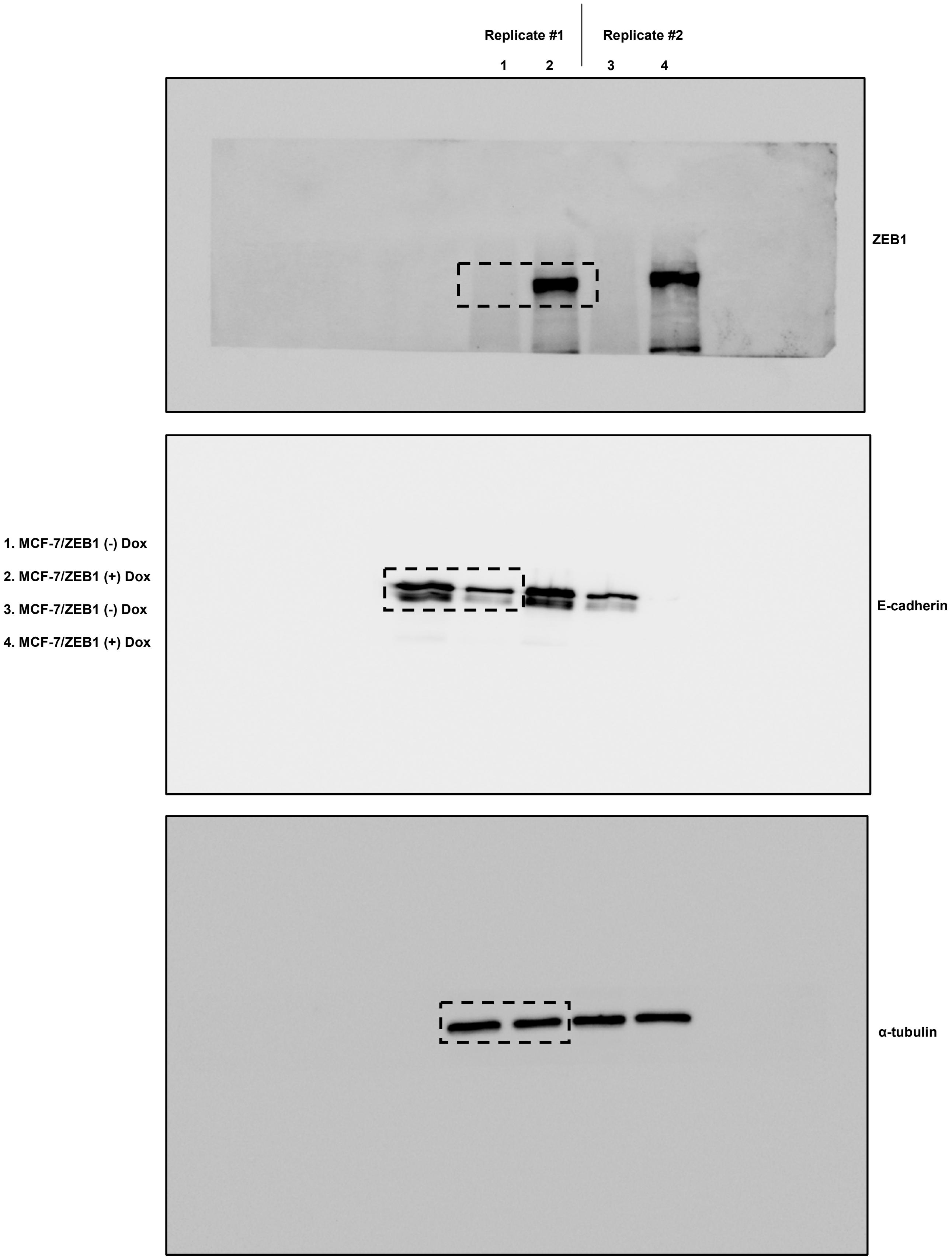

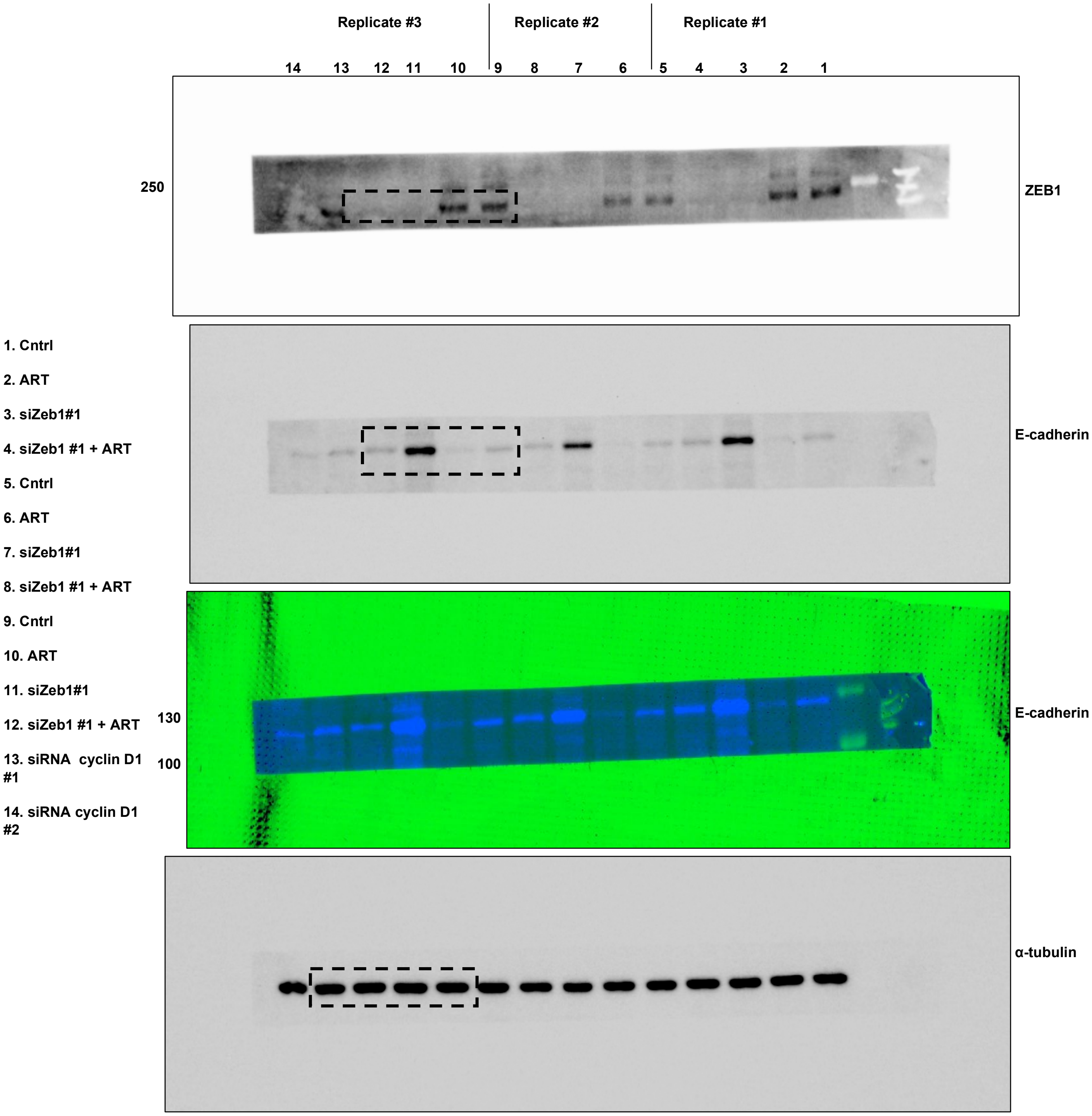

Replicate #3
Replicate #2
Replicate #1
14 13 12 11 10 9 8 7 6 5 4 3 2 1
250
ZEB1
1. Cntrl
2. ART
3. siZeb1#1
4. siZeb1 #1 + ART
5. Cntrl
6. ART
7. siZeb1#1
8. siZeb1 #1 + ART
9. Cntrl
10. ART
11. siZeb1#1
12. siZeb1 #1 + ART
13. siRNA cyclin D1 #1
14. siRNA cyclin D1 #2
E-cadherin
130
100
E-cadherin
α-tubulin

Original Western Blot files for Figure 2B

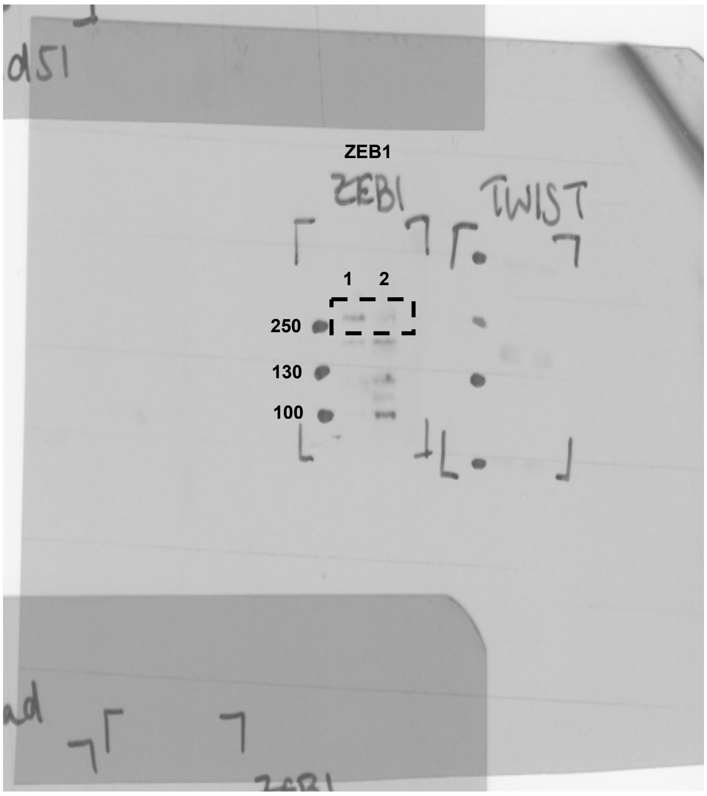

1. MDA-MB-231 sh cntrl

2. MDA-MB-231 shZEB1

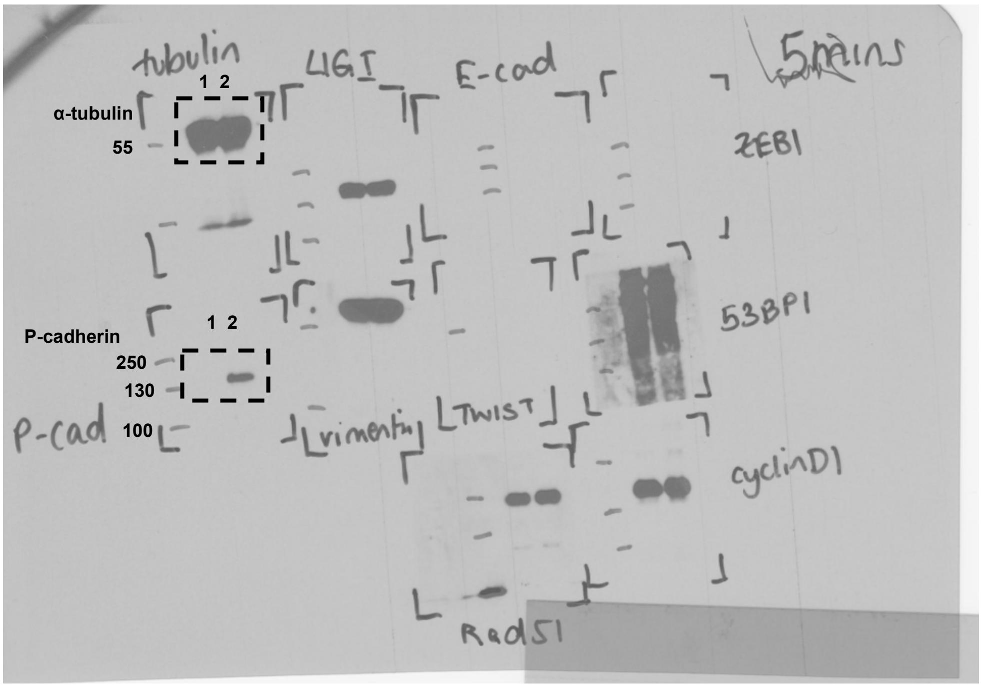

Additional experiment for Fig. 2B

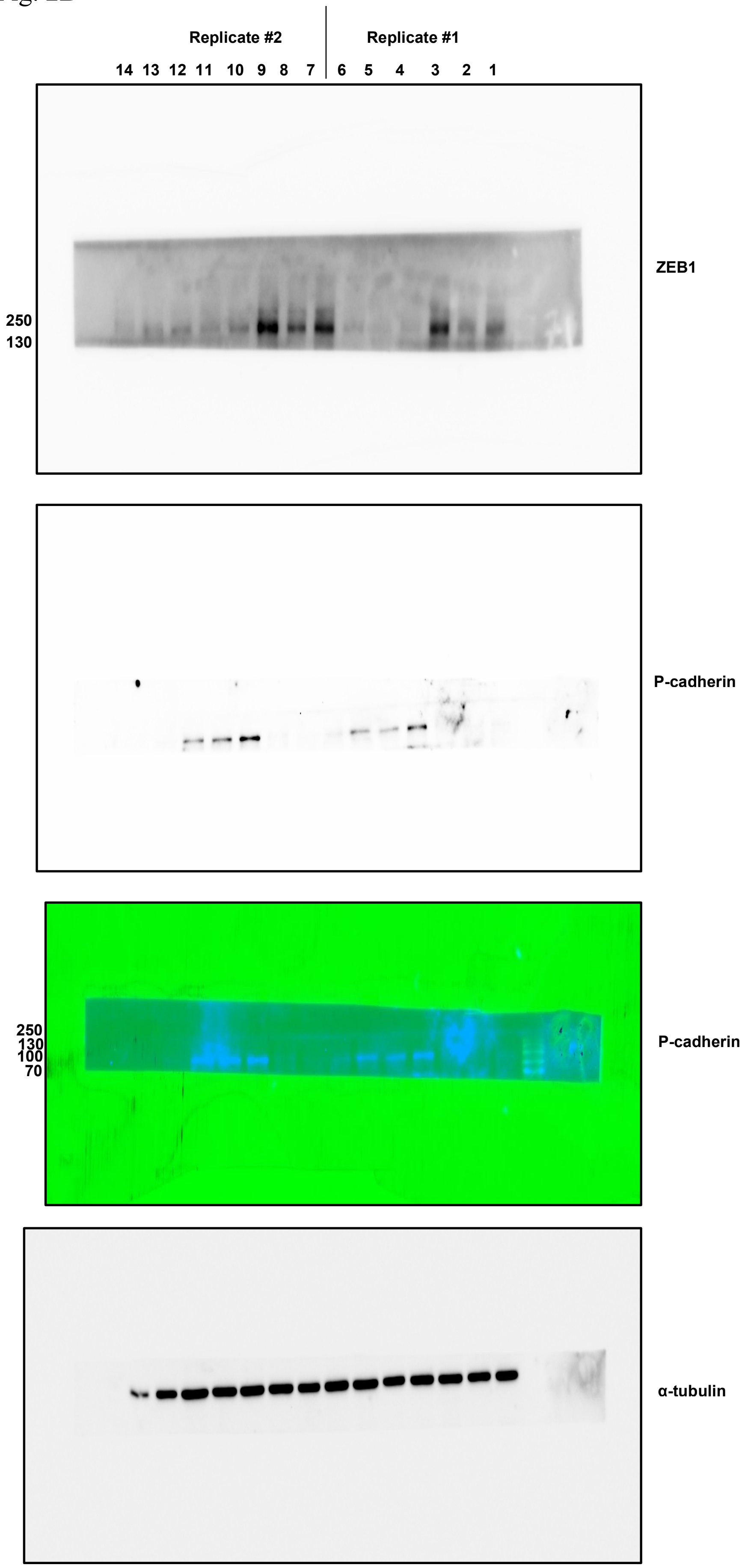

1. MDA-MB-231 cntrl
2. ART 40 µM
3. ART 160 µM
4. sh ZEB1 cntrl
5. ART 40 µM
6. ART 160 µM
7. MDA-MB-231 cntrl
8. ART 40 µM
9. ART 160 µM
10. sh ZEB1 cntrl
11. ART 40 µM
12. ART 160 µM
13. Staurosporine
14. Etoposide

Original Western Blot files for Figure 2C

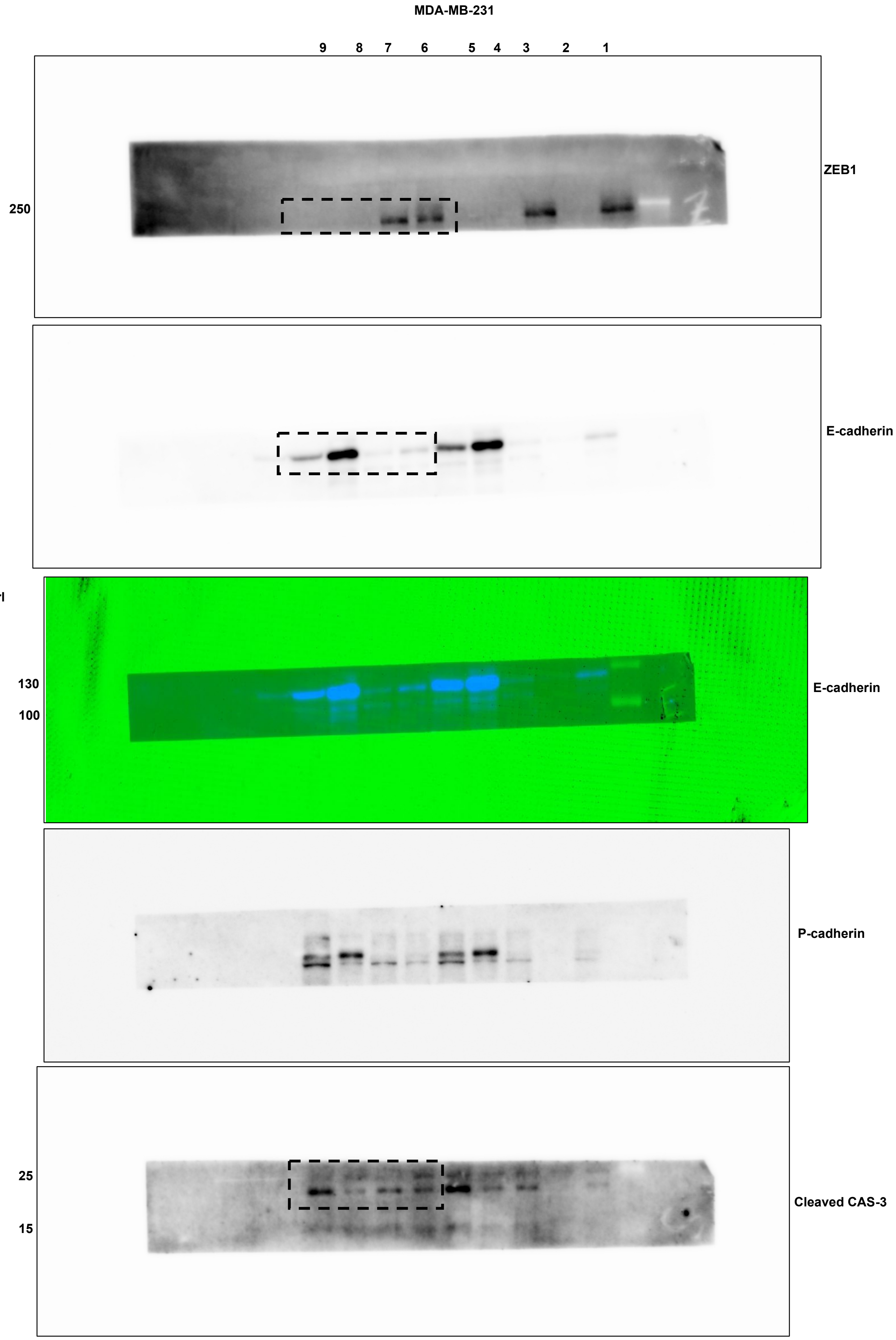

1. MDA-MB-231 Cntrl
2. Staurosporine
3. ART 40 µM
4. siRNAZeb1#1
5. siRNAZeb1 #1 + ART 40 µM
6. Cntrl
7. ART 40 µM
8. siRNAZeb1 #2
9. siRNA Zeb1 #2 + ART 40 µM

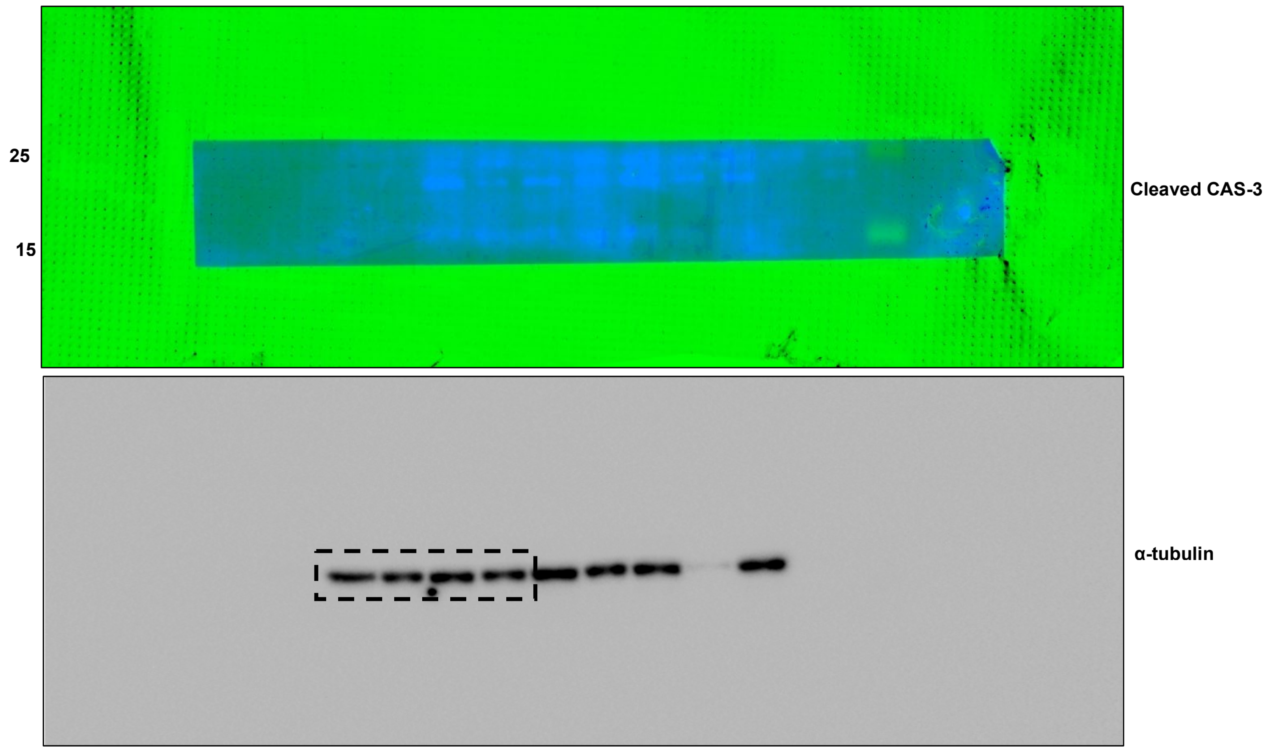
25
15
Cleaved CAS-3
α-tubulin

1. MDA-MB-436 Cntrl
2. ART 40 µM
3. siRNAZeb1#1
4. siRNAZeb1 #1 + ART 40 µM
5. siRNAZeb1 #2
6. siRNA Zeb1 #2 + ART 40 µM

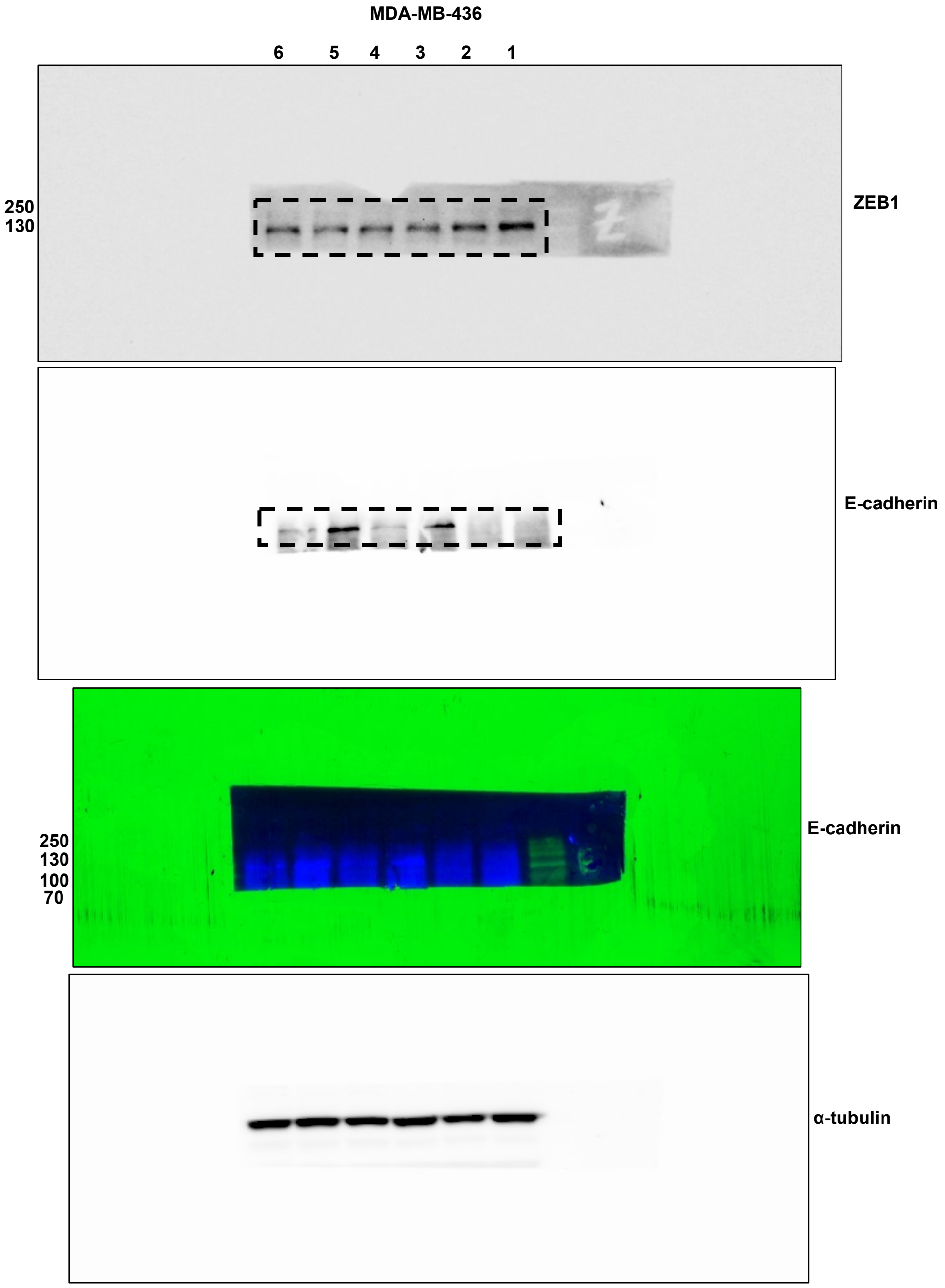

1. MDA-MB-436 Cntrl
2. ART 40 µM
3. siRNAZeb1#1
4. siRNAZeb1 #1 + ART 40 µM
5. siRNAZeb1 #2
6. siRNA Zeb1 #2 + ART 40 µM
7. Staurosporine

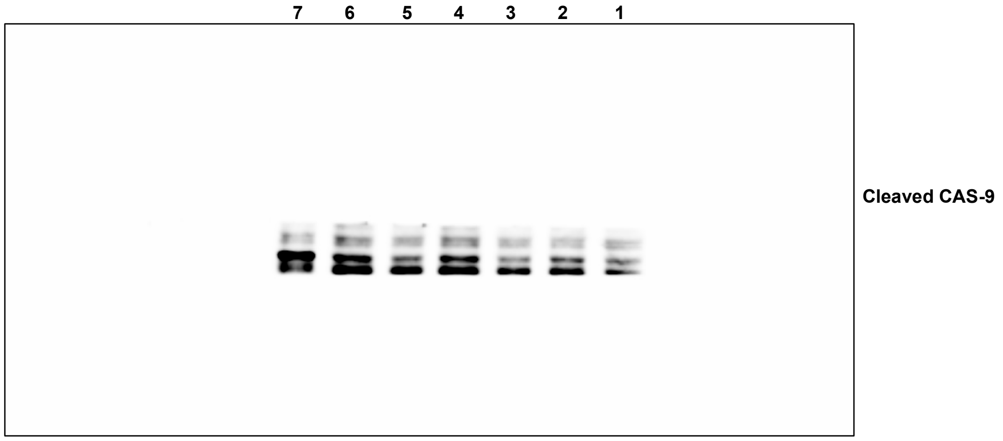

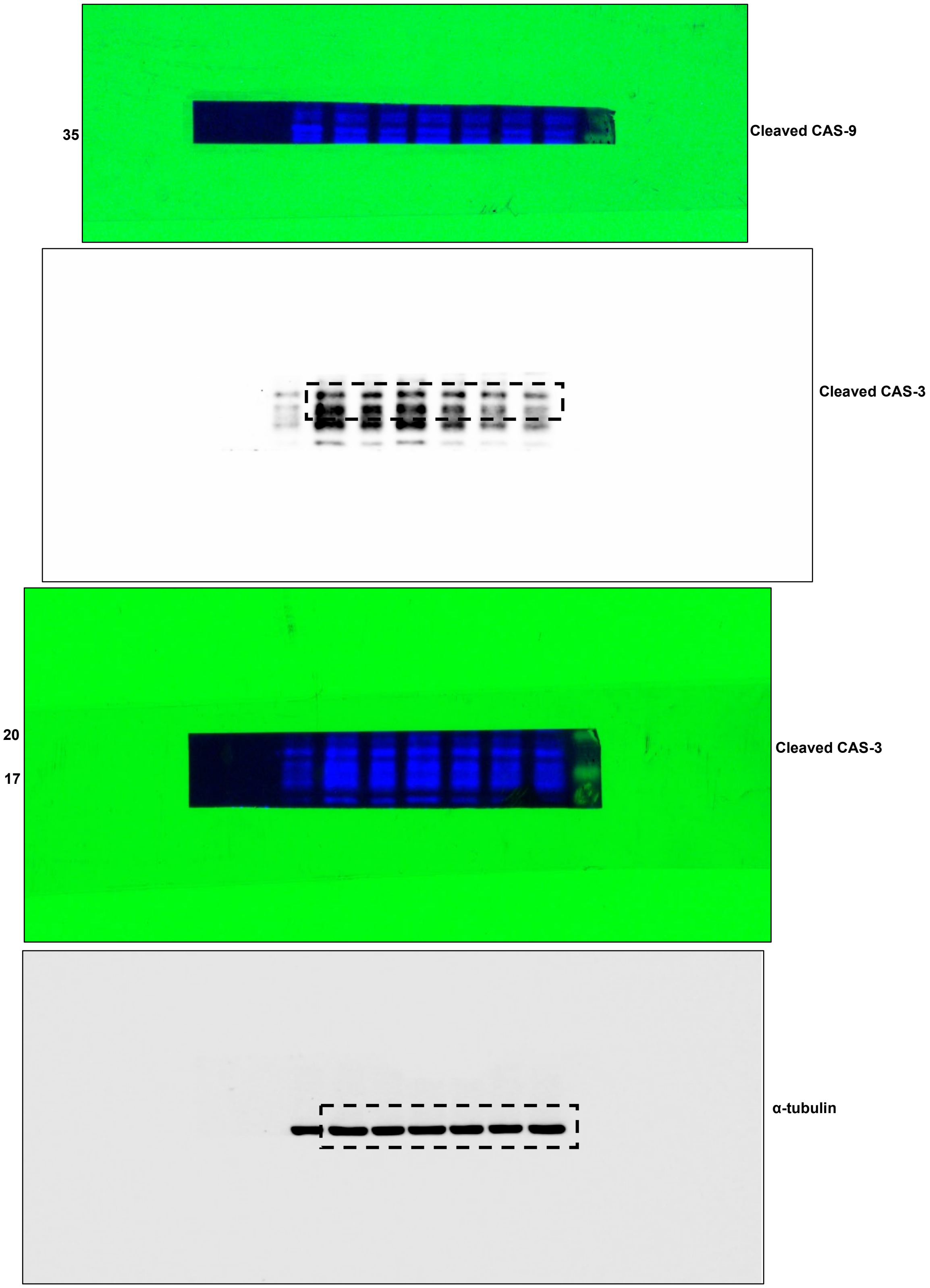

1. MDA-MB-436 Cntrl
2. ART 40 μM
3. siRNAZeb1#1
4. siRNAZeb1 #1 +
   ART 40 μM
5. siRNAZeb1 #2
6. siRNA Zeb1 #2 +
   ART 40 μM
7. Staurosporine

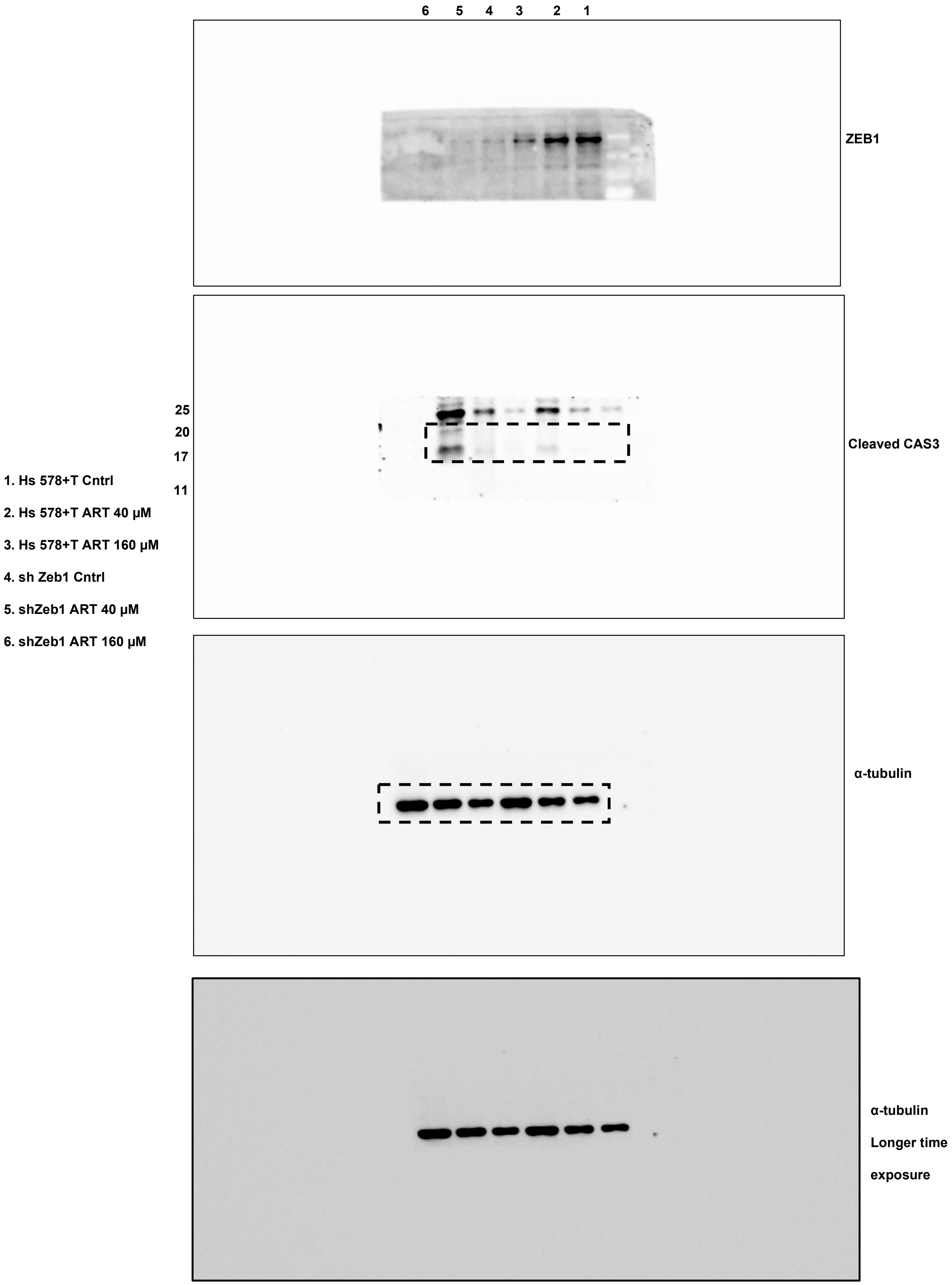
6 5 4 3 2 1
ZEB1
25
20
17
11
Cleaved CAS3
1. Hs 578+T Cntrl
2. Hs 578+T ART 40 μM
3. Hs 578+T ART 160 μM
4. sh Zeb1 Cntrl
5. shZeb1 ART 40 μM
6. shZeb1 ART 160 μM
α-tubulin
α-tubulin
Longer time
exposure

Second replicate for Hs 578-T cells

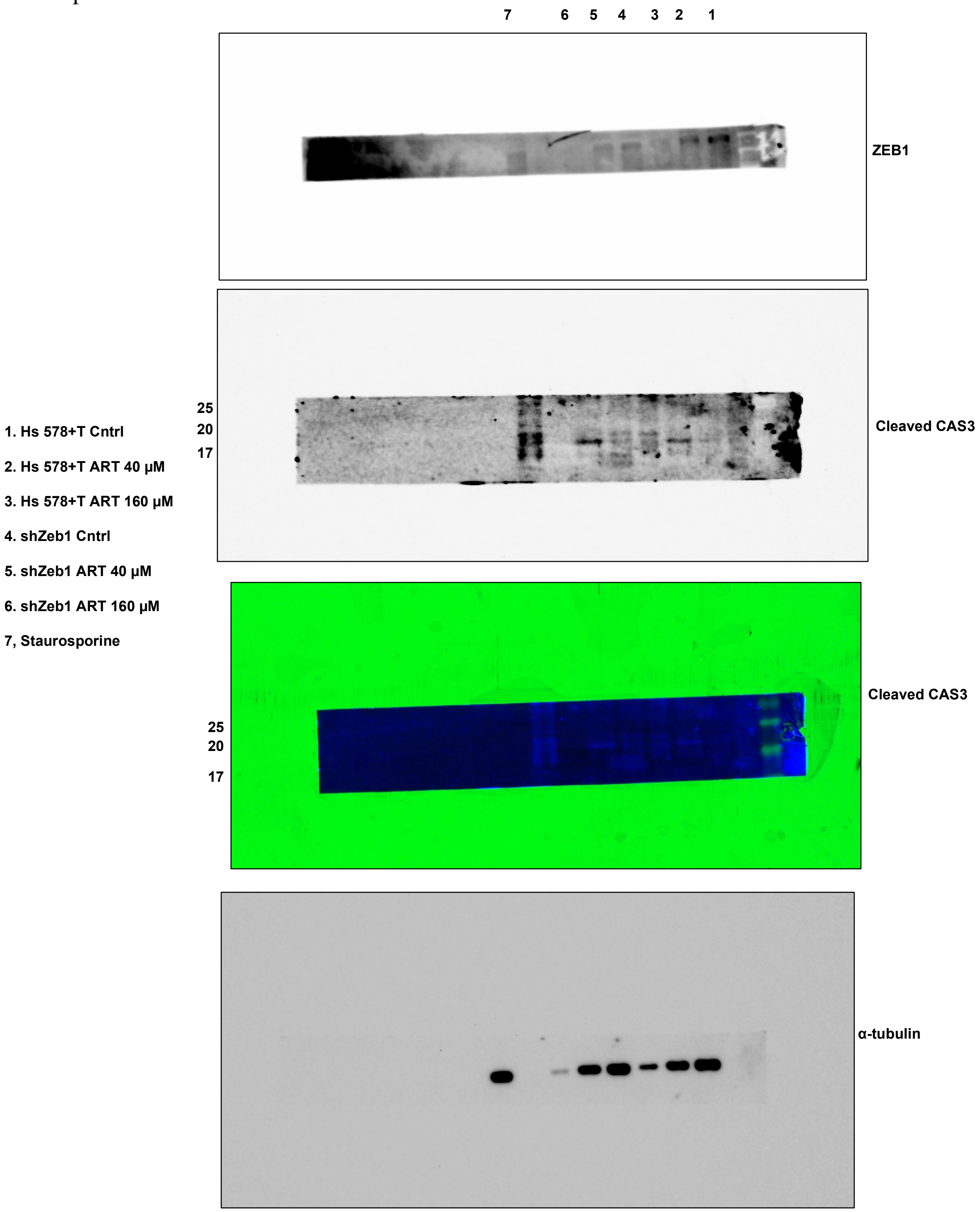

Original Western Blot files for Figure 3D

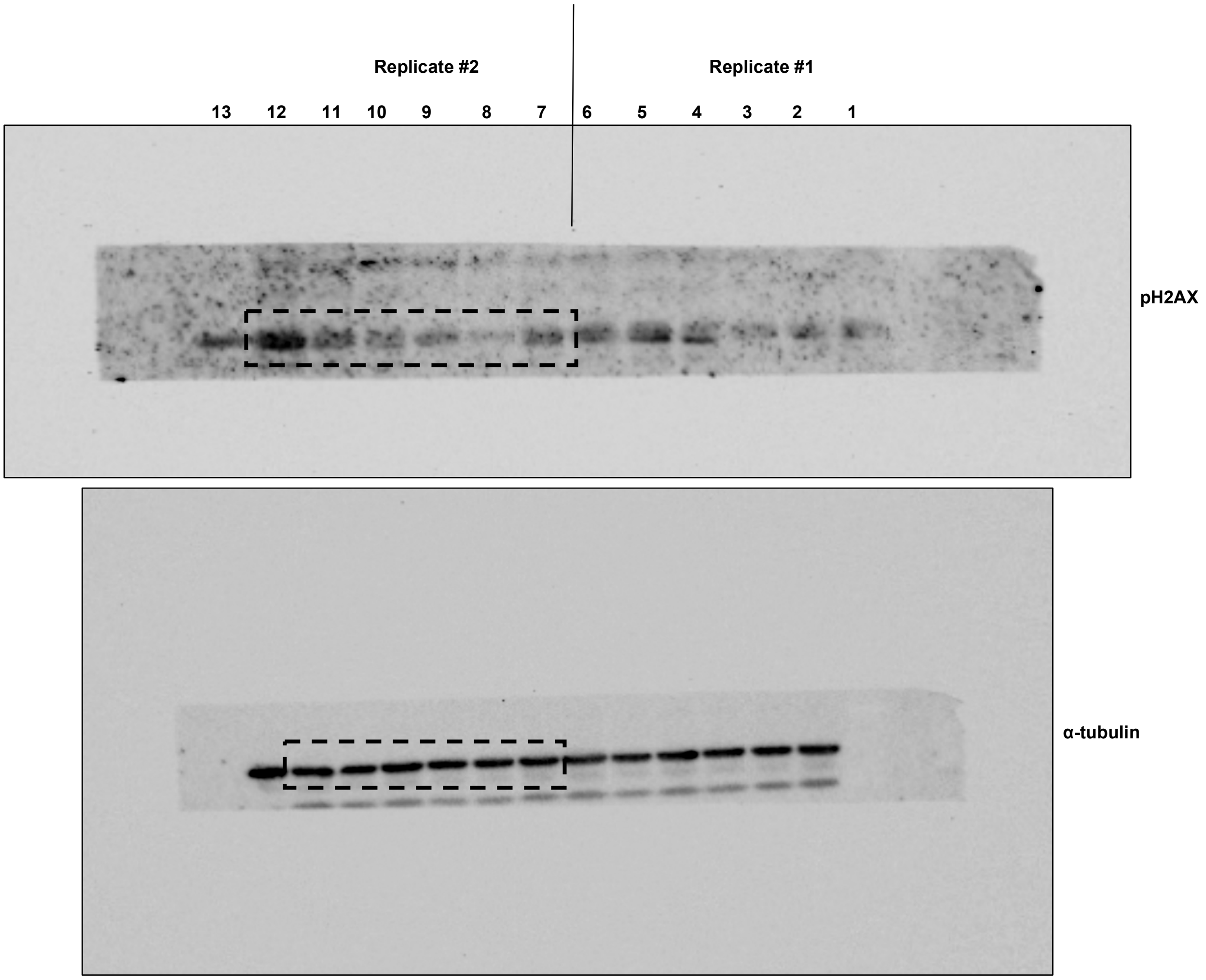

1. MDA-MB-231 cntrl
2. ART 40 μM
3. ART 160 μM
4. sh ZEB1 cntrl
5. ART 40 μM
6. ART 160 μM
7. MDA-MB-231 cntrl
8. ART 40 μM
9. ART 160 μM
10. sh ZEB1 cntrl
11. ART 40 μM
12. ART 160 μM
13. Staurosporine

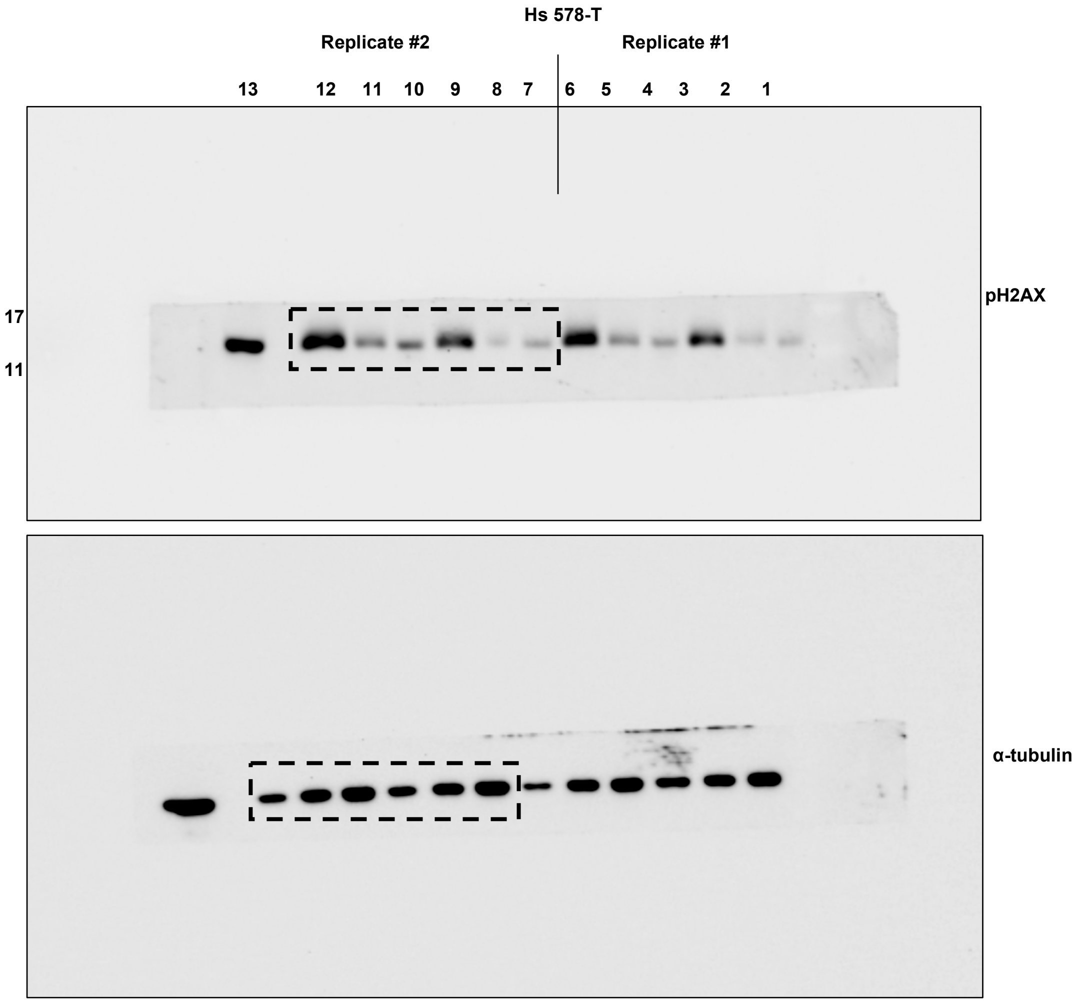

1. Hs 578+T Cntrl
2. Hs 578+T ART 40 μM
3. Hs 578+T ART 160 μM
4. sh Zeb1 Cntrl
5. shZeb1 ART 40 μM
6. shZeb1 ART 160 μM

. Hs 578+T Cntrl

8. Hs 578+T ART 40 μM
9. Hs 578+T ART 160 μM
10. sh Zeb1 Cntrl
11. shZeb1 ART 40 μM
12. shZeb1 ART 160 μM
13. Staurosporine

Additional experiment for MDA-MB-231 cells treated with siRNA ZEB1 pus ART to check effect on pH2AX

1. Staurosporine

2. MDA-MB-231 cntrl

3. ART 40 μM

4. siRNAZeb1#1

5. siRNAZeb1 #1 +
ART 40 μM

6. Cntrl

7. ART 40 μM

8. siRNAZeb1 #2

9. siRNA Zeb1 #2 +
ART 40 μM

10. Etoposide 25 μM

11. Etoposide 50 μM

12. Etoposide 100 μM

13. Etoposide 200 μM

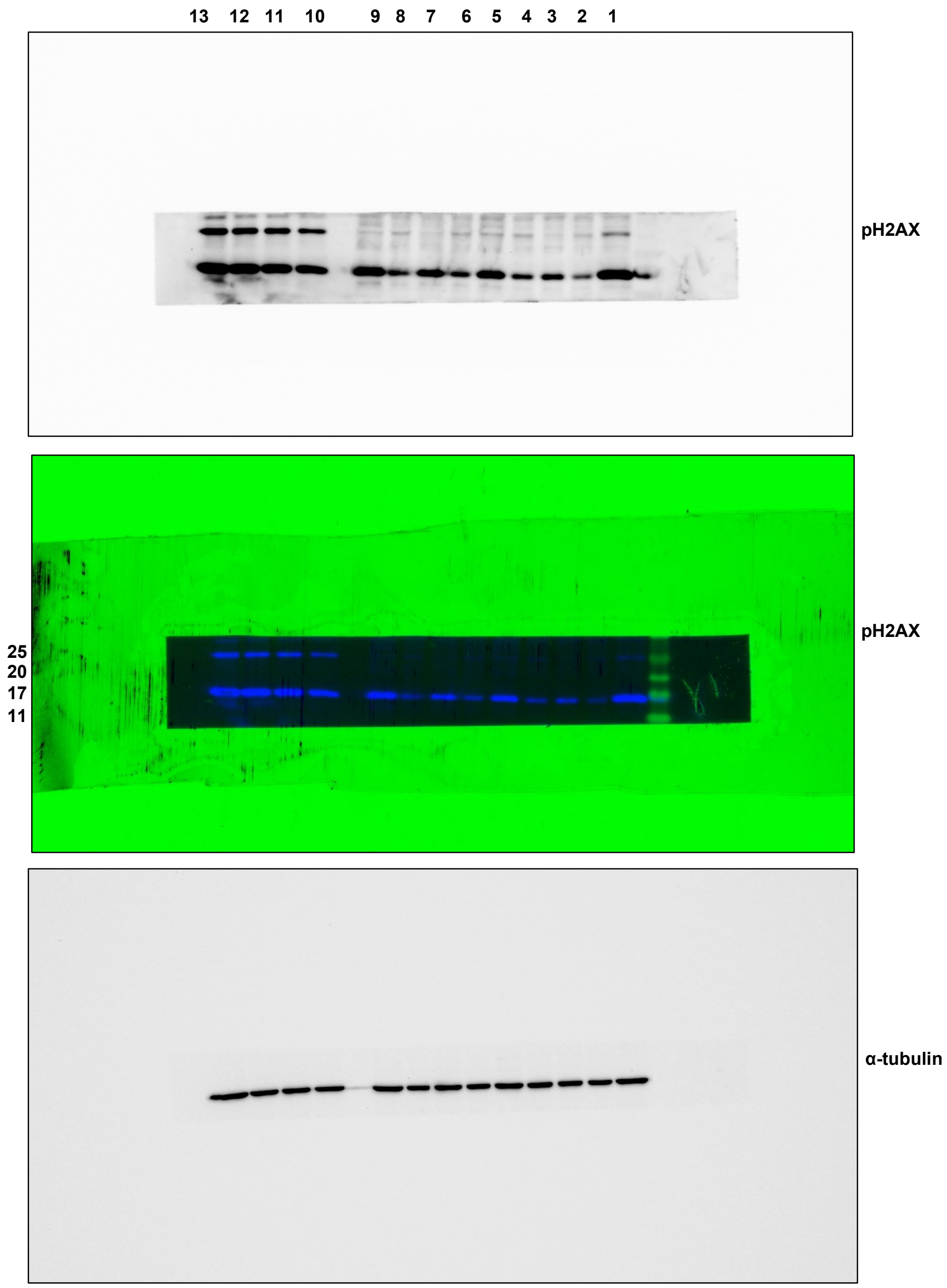

Original Western Blot files for Figure 4A

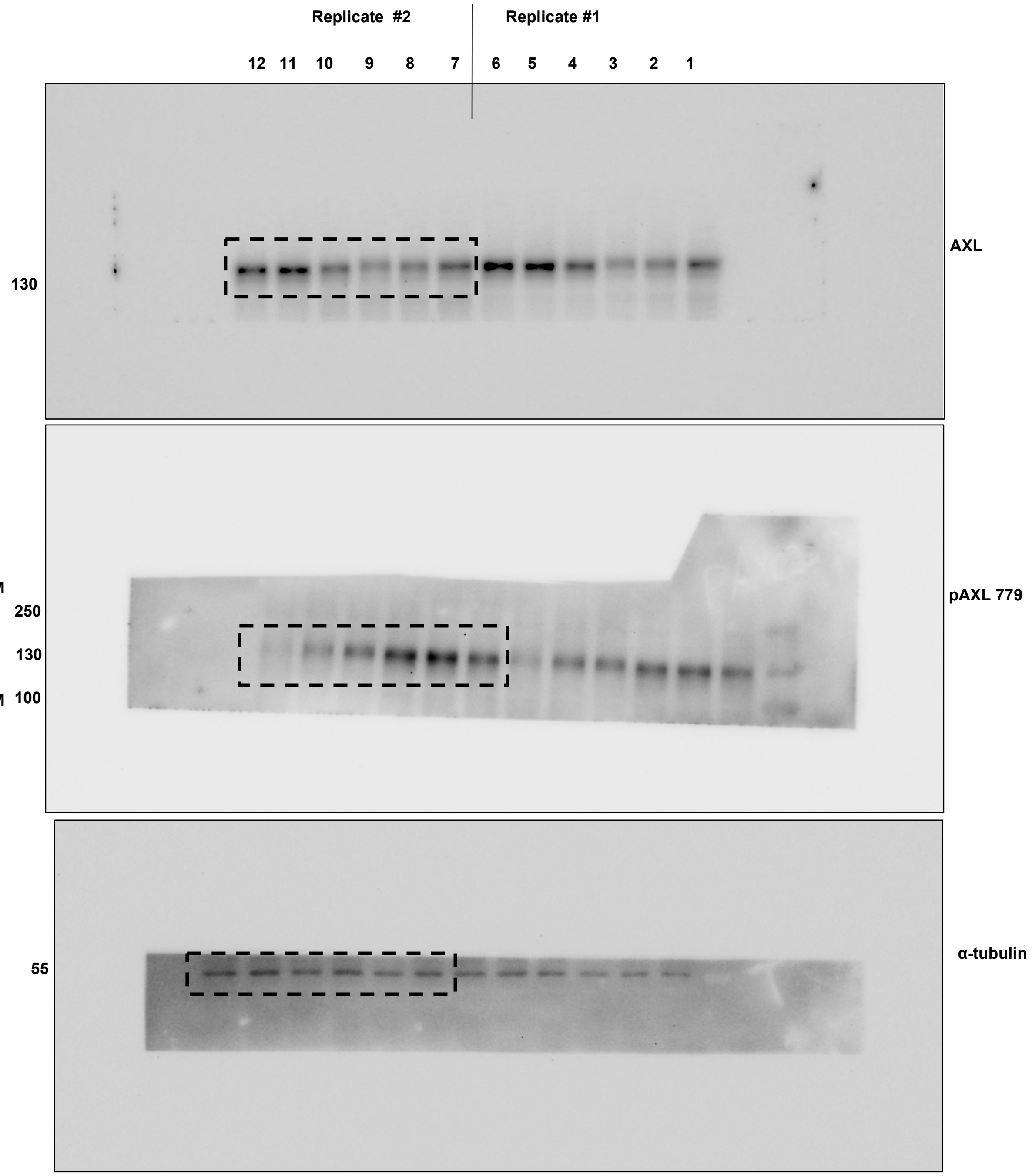

1. MDA-MB-231 Control
2. MDA-MB-231 TP 0,06 µM
3. MDA-MB-231 TP 0,125 µM
4. MDA-MB-231 TP 0,25 µM
5. MDA-MB-231 TP 0,5 µM
6. MDA-MB-231 TP 1 µM
7. MDA-MB-231 Control
8. MDA-MB-231 TP 0,06 µM
9. MDA-MB-231 TP 0,125 µM
10. MDA-MB-231 TP 0,25 µM
11. MDA-MB-231 TP 0,5 µM
12. MDA-MB-231 TP 1 µM

Third replicate for MDA-MB-231 cells to check TP-0903 effect on AXL and pAXL 779

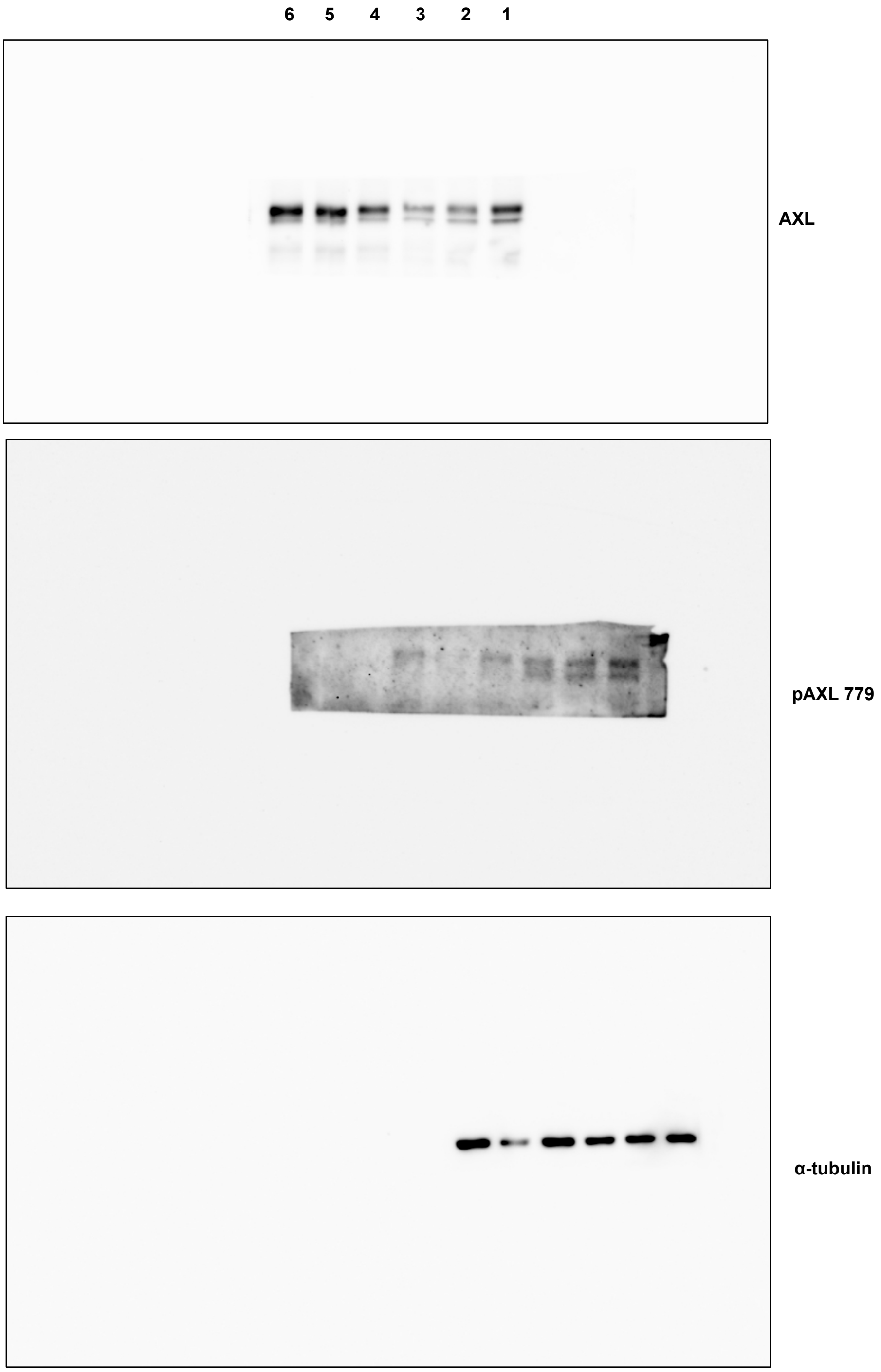

1. **MDA-MB-231 Control**
2. **MDA-MB-231 TP 0,06 µM**
3. **MDA-MB-231 TP 0,125 µM**
4. **MDA-MB-231 TP 0,25 µM**
5. **MDA-MB-231 TP 0,5 µM**
6. **MDA-MB-231 TP 1 µM**

Original Western Blot files for Figure 4B

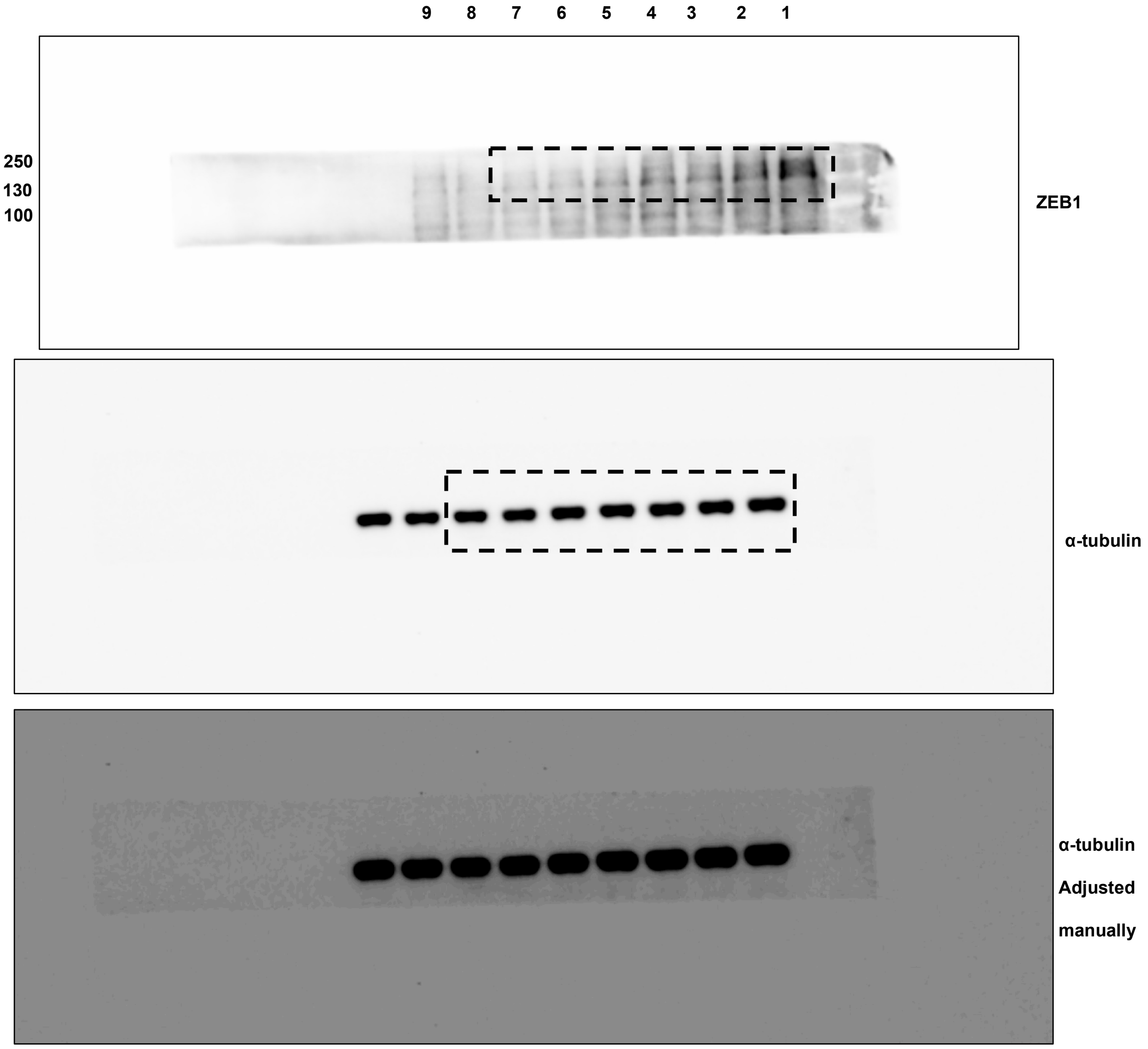

1. **MDA-MB-231 Control**
2. **MDA-MB-231 TP 0,015 μM**
3. **MDA-MB-231 TP 0,03 μM**
4. **MDA-MB-231 TP 0,06 μM**
5. **MDA-MB-231 TP 0,125 μM**
6. **MDA-MB-231 TP 0,25 μM**
7. **MDA-MB-231 TP 0,5 μM**
8. **MDA-MB-231 TP 1 μM**
9. **MDA-MB-231 TP 2 μM**

Second replicate for MDA-MB-231 cells to check TP-0903 effect on ZEB1

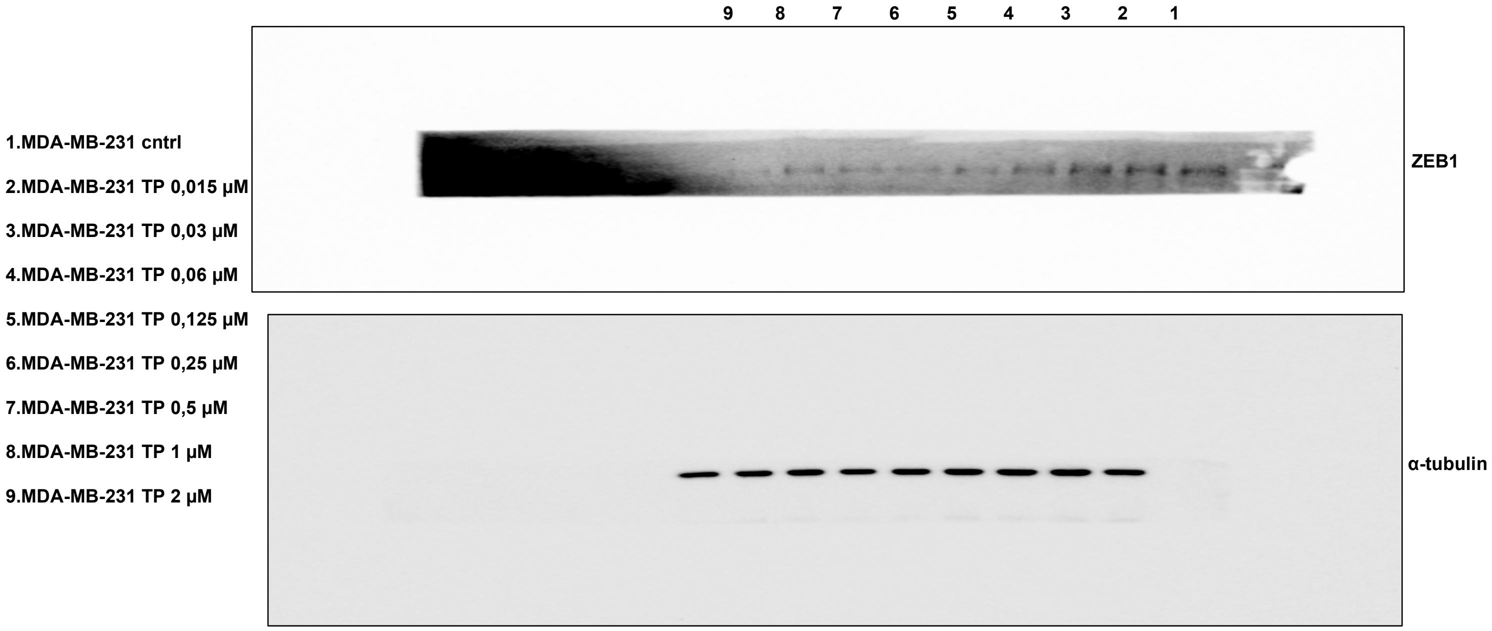

Third replicate for MDA-MB-231 cells to check TP-0903 effect on ZEB1

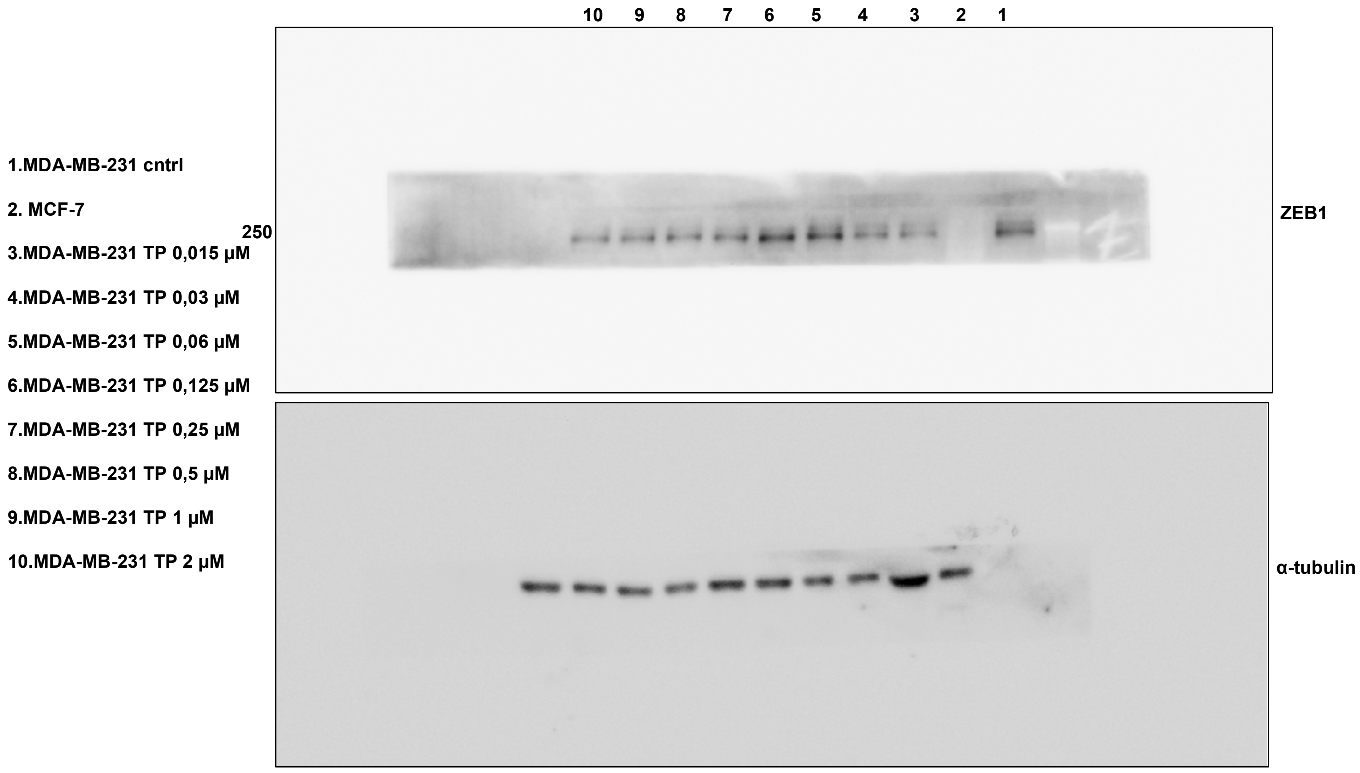

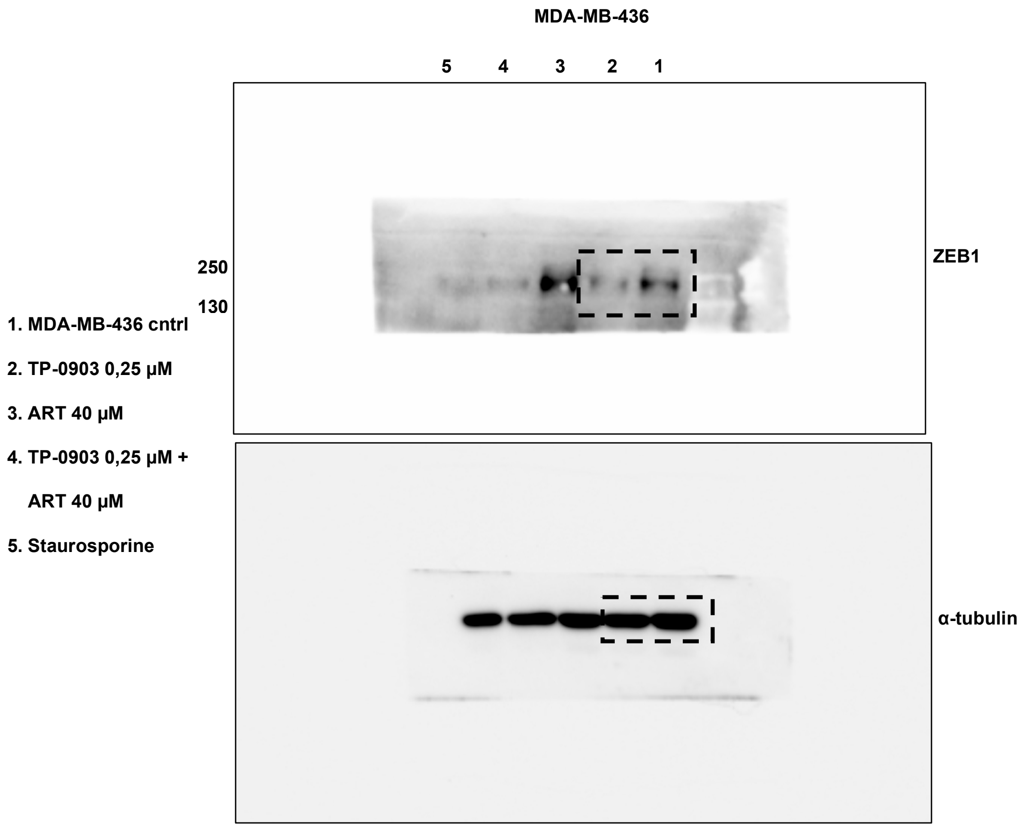
MDA-MB-436
5
4
3
2
1
250
130
ZEB1
α-tubulin
1. MDA-MB-436 cntrl
2. TP-0903 0,25 µM
3. ART 40 µM
4. TP-0903 0,25 µM + ART 40 µM
5. Staurosporine

3 2 1

130

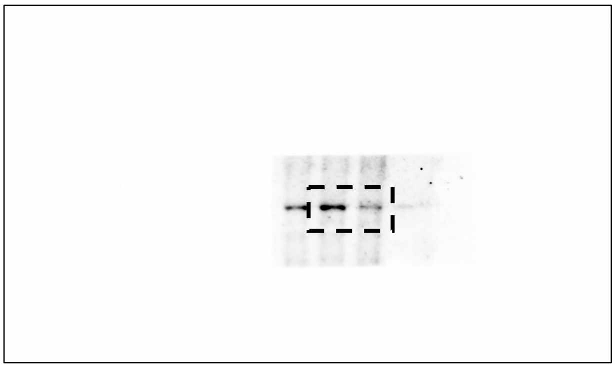

E-cad

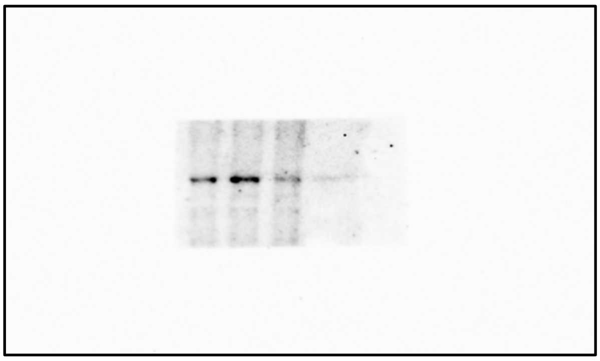

E-cad
Longer time
exposure

1. MDA-MB-231 Control
2. MDA-MB-231 TP-0903 0,25 µM
3. MDA-MB-231 TP-0903 0,5 µM

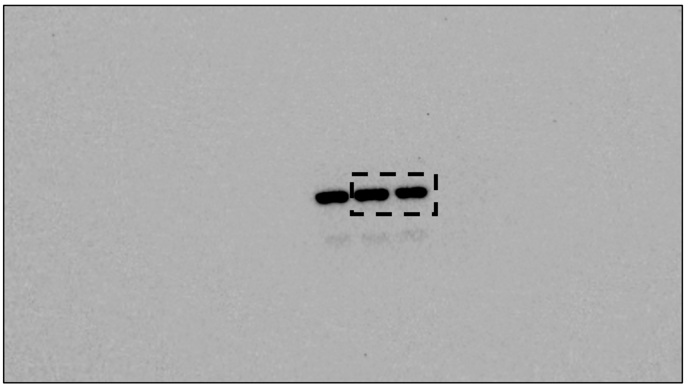

α-tubulin

2 1

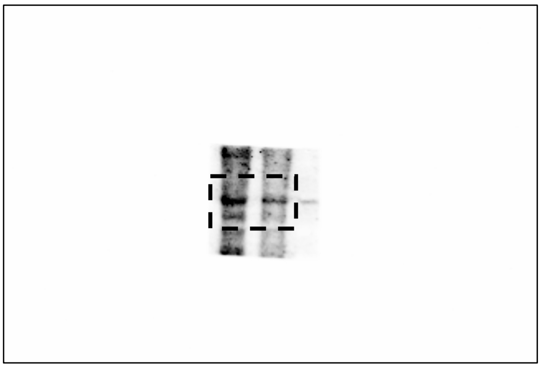

E-cad

1. MDA-MB-436 Control
2. MDA-MB-436 TP-0903 0,125 µM

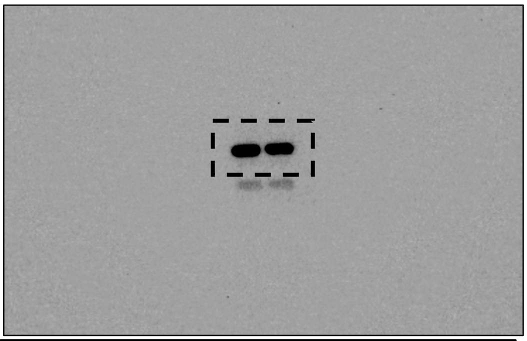

α-tubulin

130

100

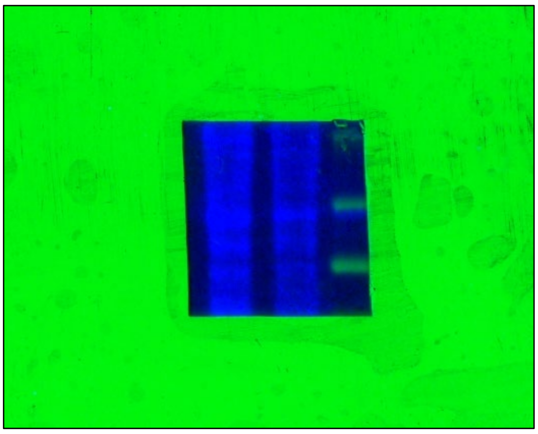

E-cad

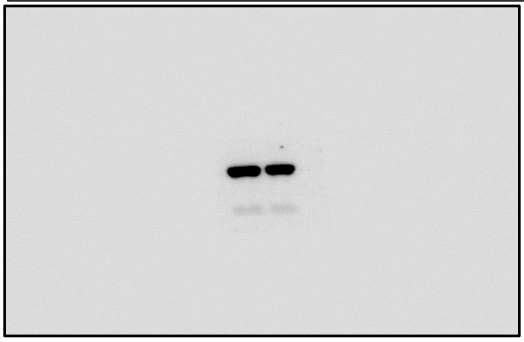

α-tubulin
Longer time
exposure

2 1

130

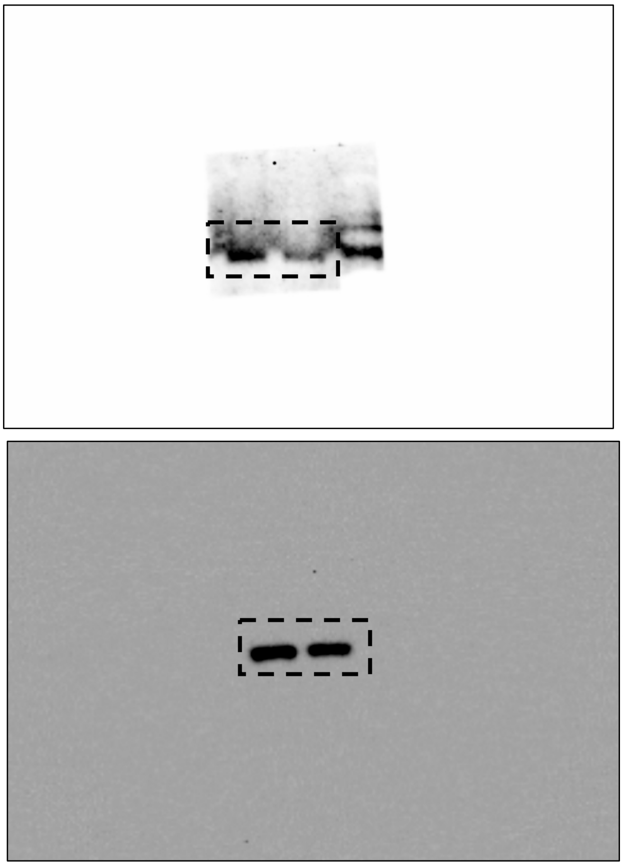

E-cad

α-tubulin

1. Hs 578-T Control
2. Hs 578-T TP-0903 0,25 µM

Original Western Blot files for Figure 4E

1. MDA-MB-231 cntrl
2. Staurosporine
3. TP-0903 0,25 µM Pre-treat
4. TP-0903 0,25 µM 72 h
5. ART 10 µM
6. ART 20 µM
7. ART 40 µM
8. TP0903 0,25 µM + ART 10 µM
9. TP0903 0,25 µM + ART 20 µM
10. TP0903 0,25 µM + ART 40 µM

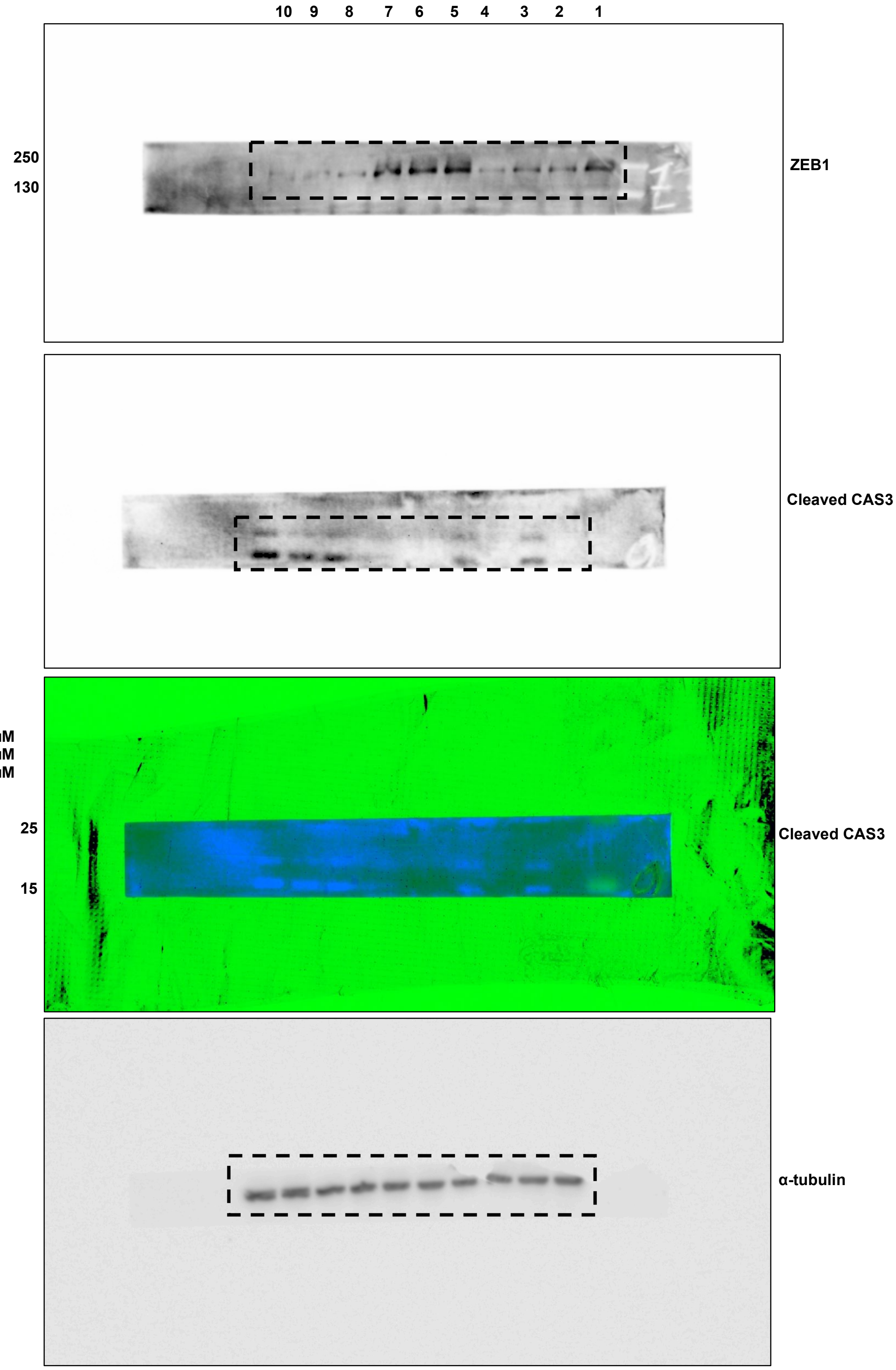

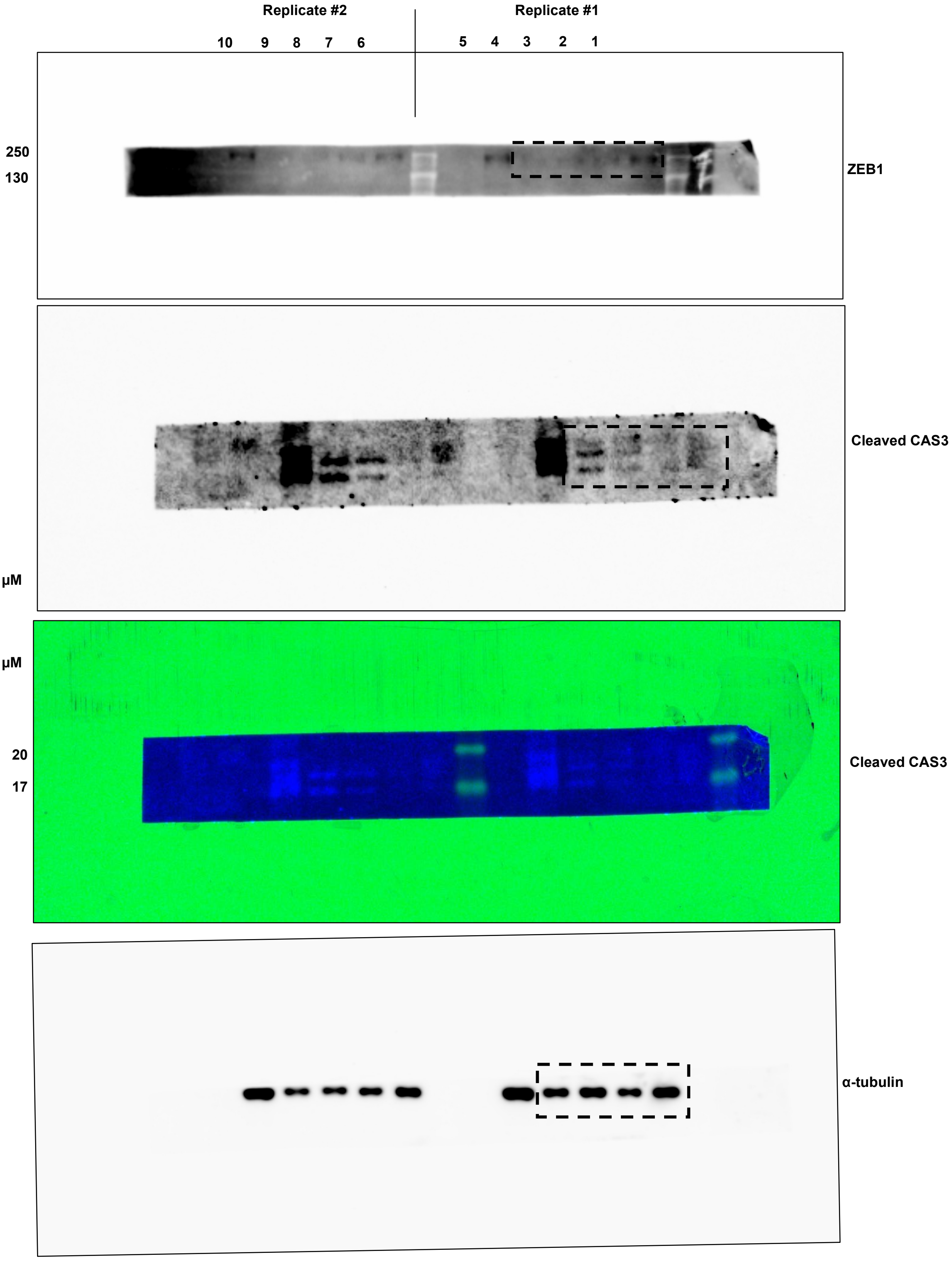

1. Hs 578-T cntrl
2. ART 40 µM
3. TP-0903 0,25 µM
4. TP0903 0,25 µM + ART 40 µM
5. Staurosporine
6. Hs 578-T cntrl
7. ART 40 µM
8. TP-0903 0,25 µM
9. TP0903 0,25 µM + ART 40 µM
10. Staurosporine

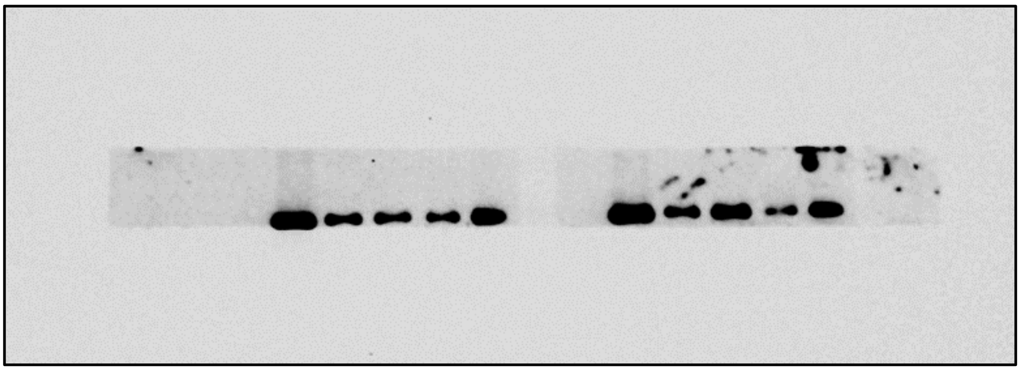

α-tubulin

Longer time exposure

Original Western Blot files for Figure 5B

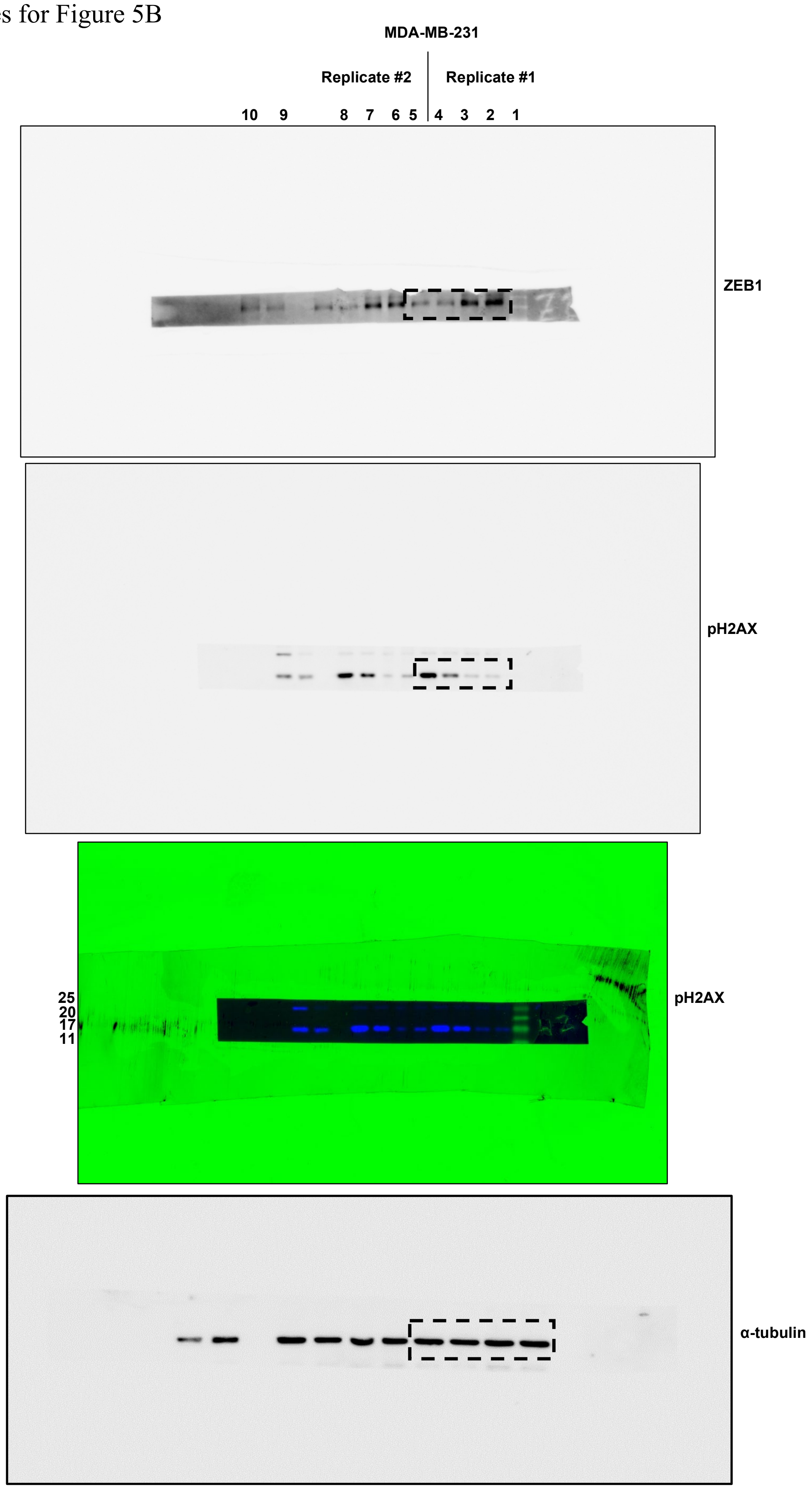

1. MDA-MB-231 cntrl

3. ART 40 μM

3. TP-0903 0,25 μM

4. TP-0903 0,25 μM + ART 40 μM

5. MDA-MB-231 cntrl

6. ART 40 μM

7. TP-0903 0,25 μM

8. TP-0903 0,25 μM + ART 40 μM

9. Staurosporine

10. Etoposide

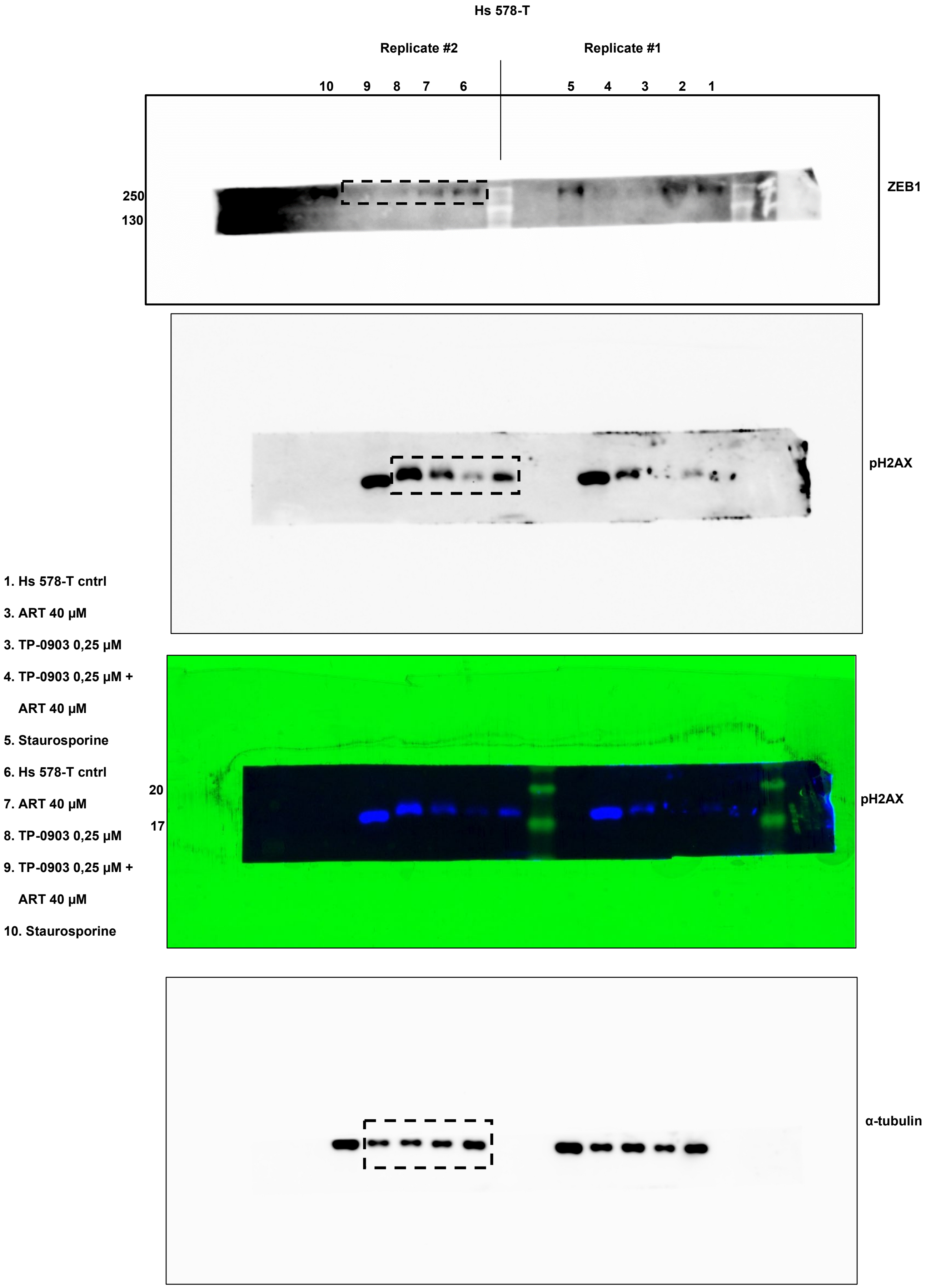
Hs 578-T
Replicate #2
Replicate #1
10 9 8 7 6
5 4 3 2 1
250
130
ZEB1
pH2AX
20
17
pH2AX
α-tubulin
1. Hs 578-T cntrl
3. ART 40 µM
3. TP-0903 0,25 µM
4. TP-0903 0,25 µM + ART 40 µM
5. Staurosporine
6. Hs 578-T cntrl
7. ART 40 µM
8. TP-0903 0,25 µM
9. TP-0903 0,25 µM + ART 40 µM
10. Staurosporine

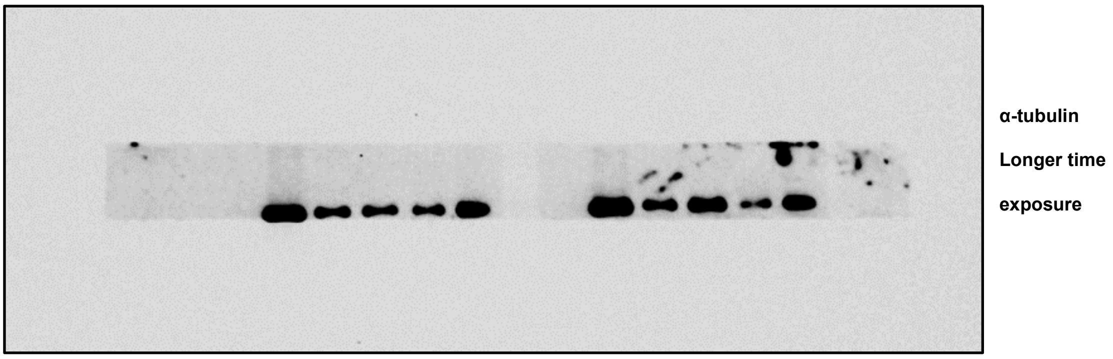

Additional experiment for TP-0903, ART, TP-0903 plus ART effect on ZEB1 in Hs 578-T cells

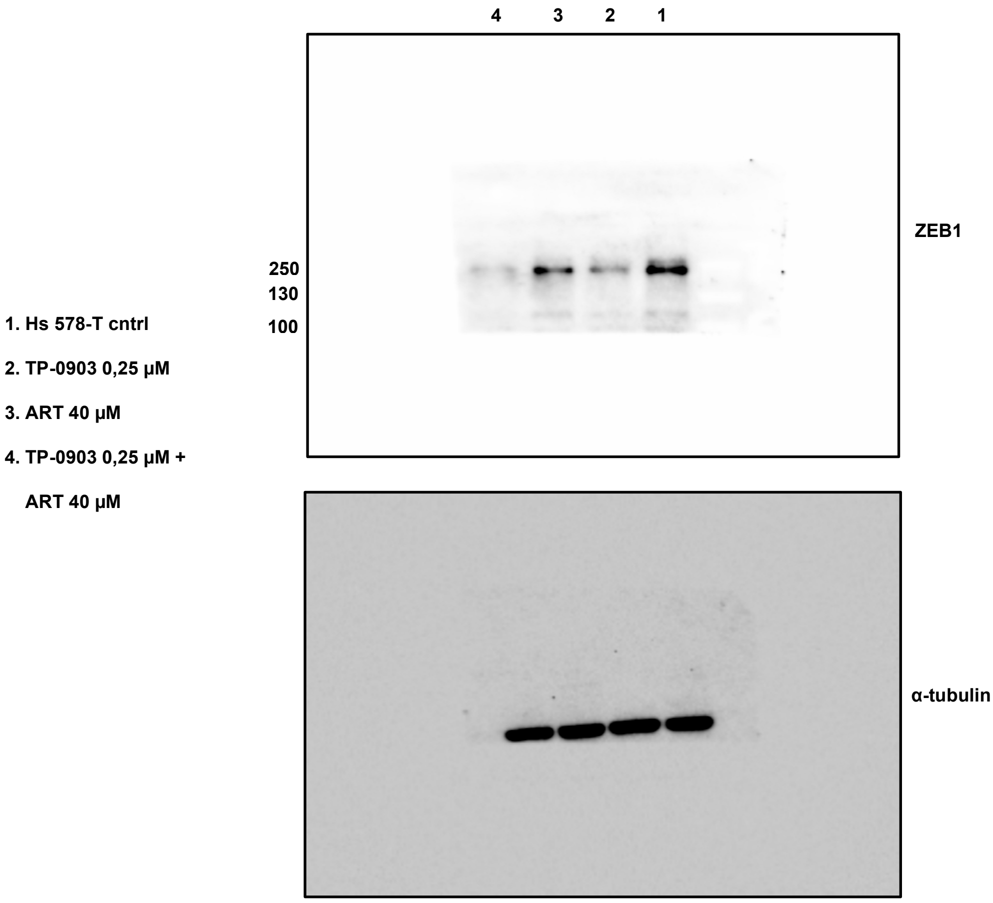

Original Western Blot files for Figure 5C

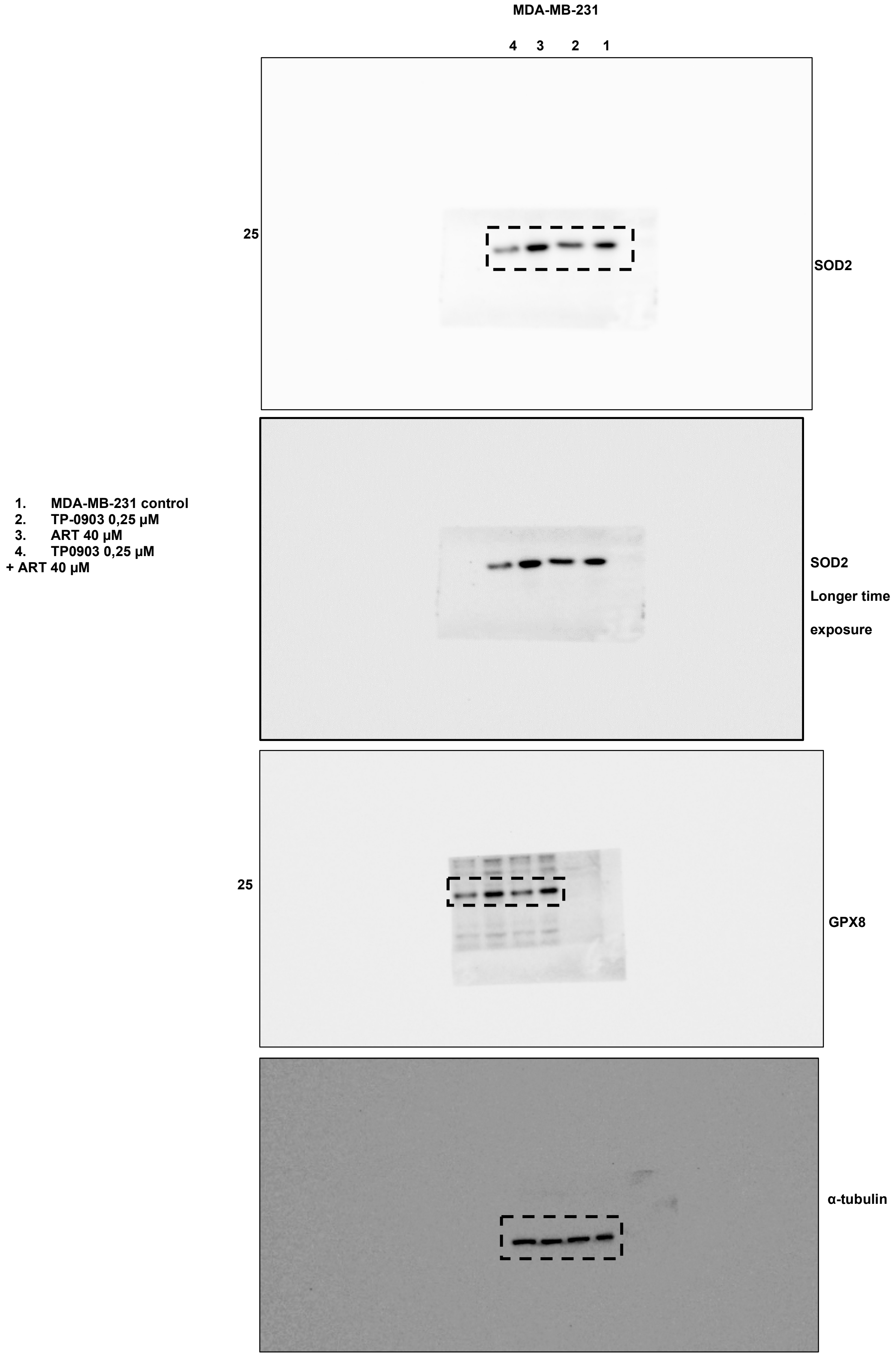

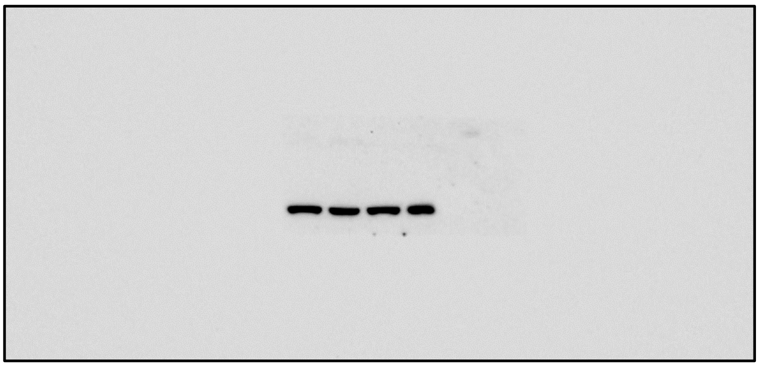

α-tubulin

Longer time

exposure

4 3 2 1

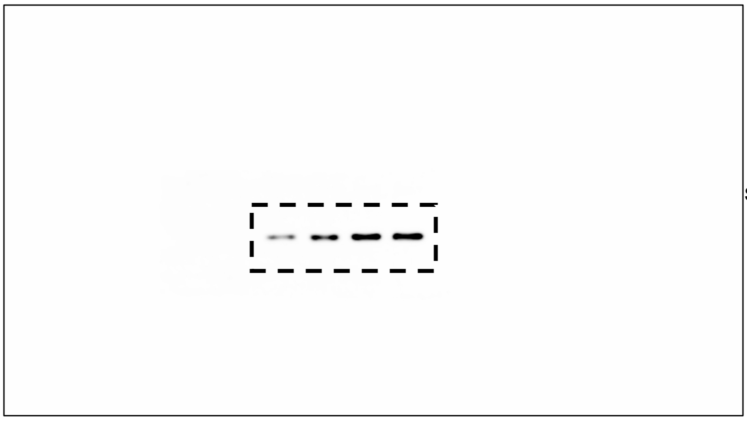

SOD2

1. Hs 578-T ontrol
2. TP-0903 0,25 µM
3. ART 40 µM
4. TP0903 0,25 µM
+ ART 40 µMvv

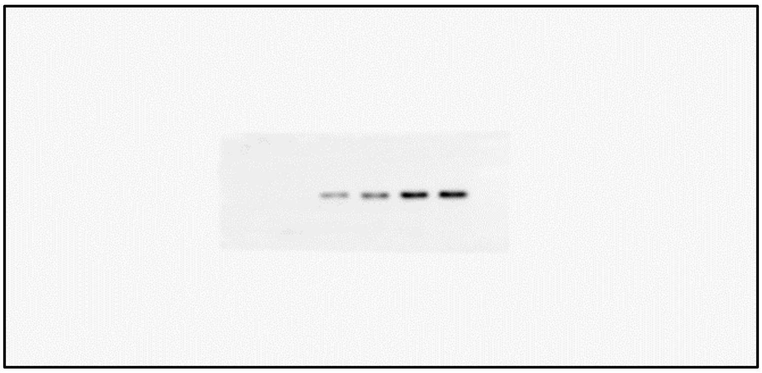

SOD2

Longer time

exposure

35

25

15

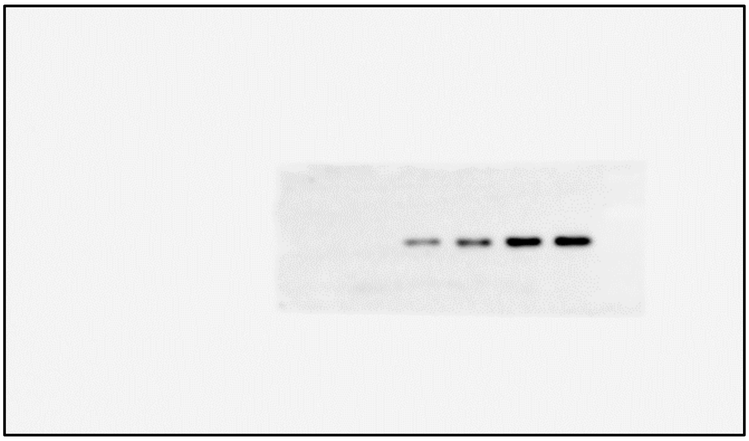

SOD2

Longer time

exposure

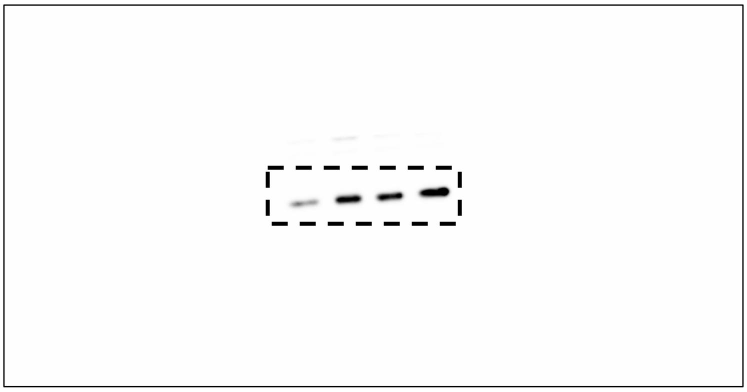

GPX8

35

25

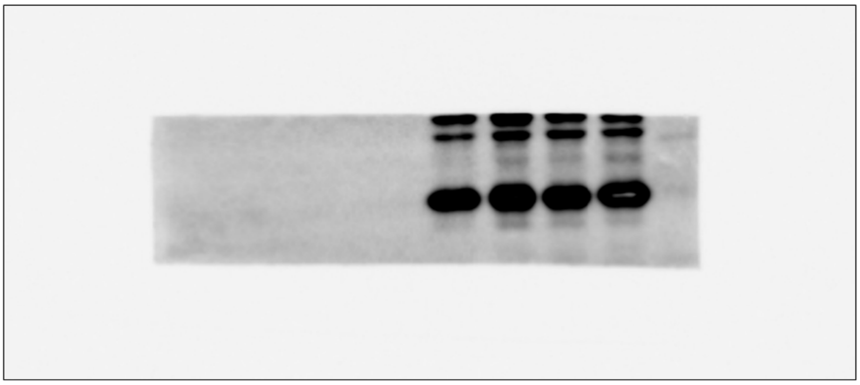

GPX8

Longer time

exposure

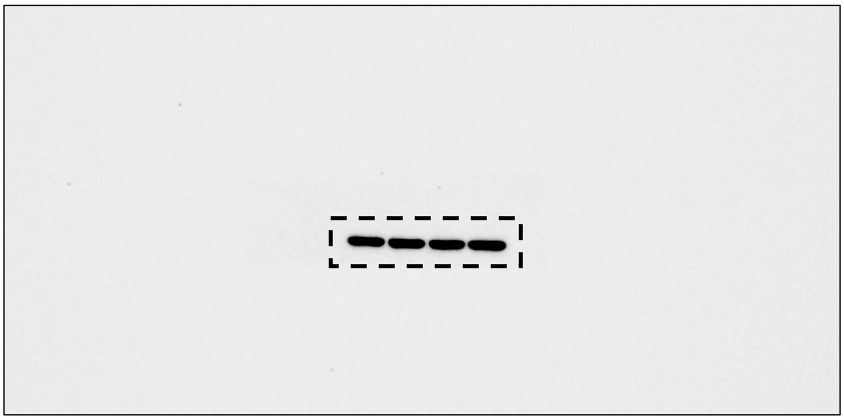

α-tubulin

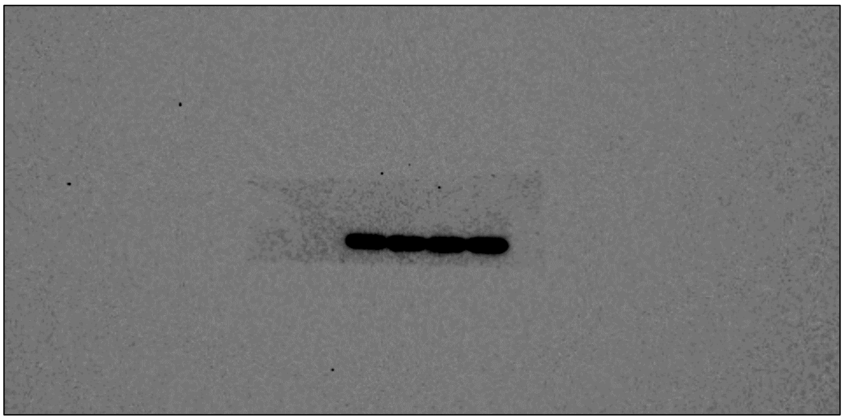

α-tubulin

Adjusted manually

Second replicate for MDA-MB-231 cells to check TP-0903 plus ART effect on SOD2 and GPX8

4 3 2 1

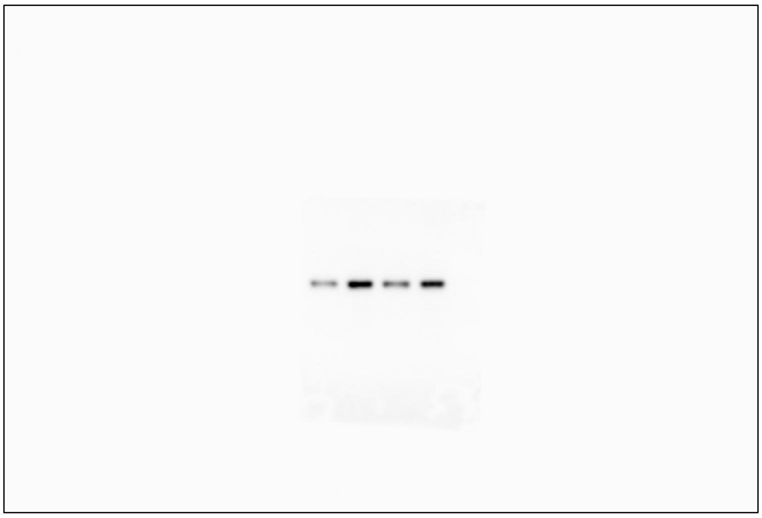

SOD2

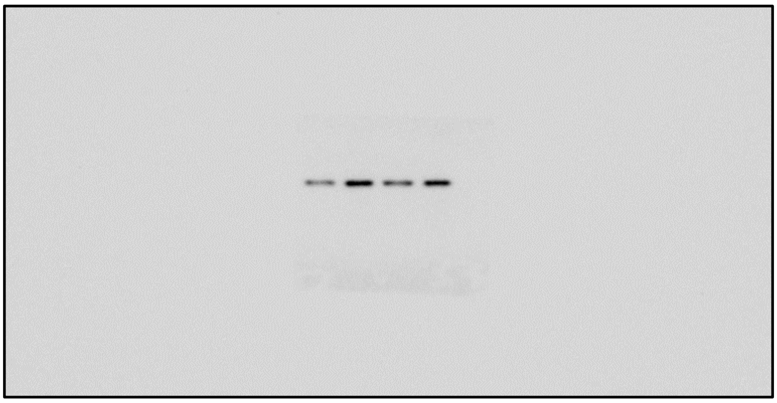

SOD2

Longer time

exposure

1.MDA-MB-231 control

2.TP-0903 0,25 µM

3.ART 40 µM

4.TP0903 0,25 µM

+ ART 40 µM

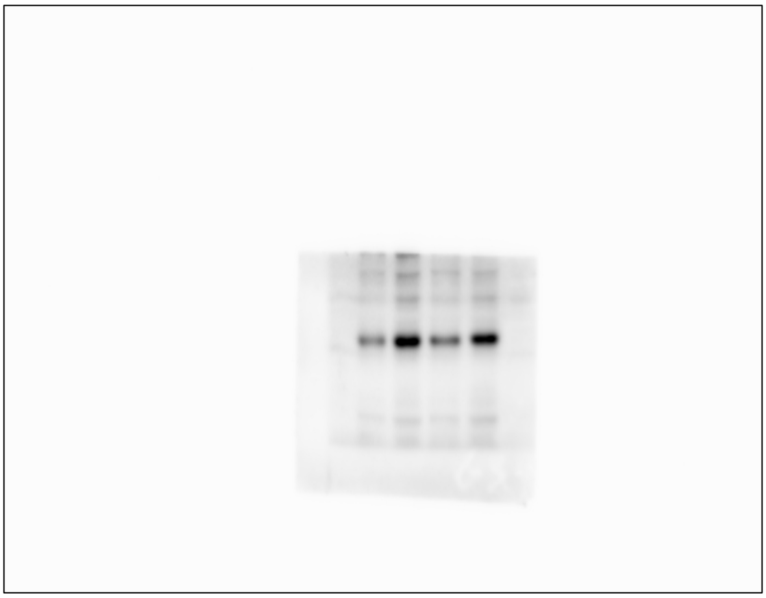

GPX8

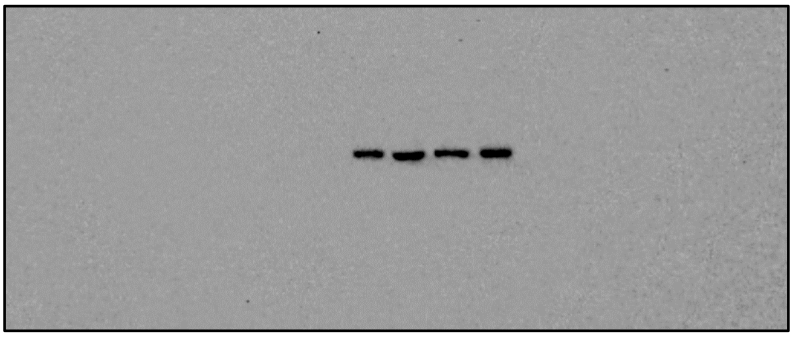
α-tubulin

Third replicate for MDA-MB-231 cells to check TP-0903 plus ART effect on SOD2 and GPX8

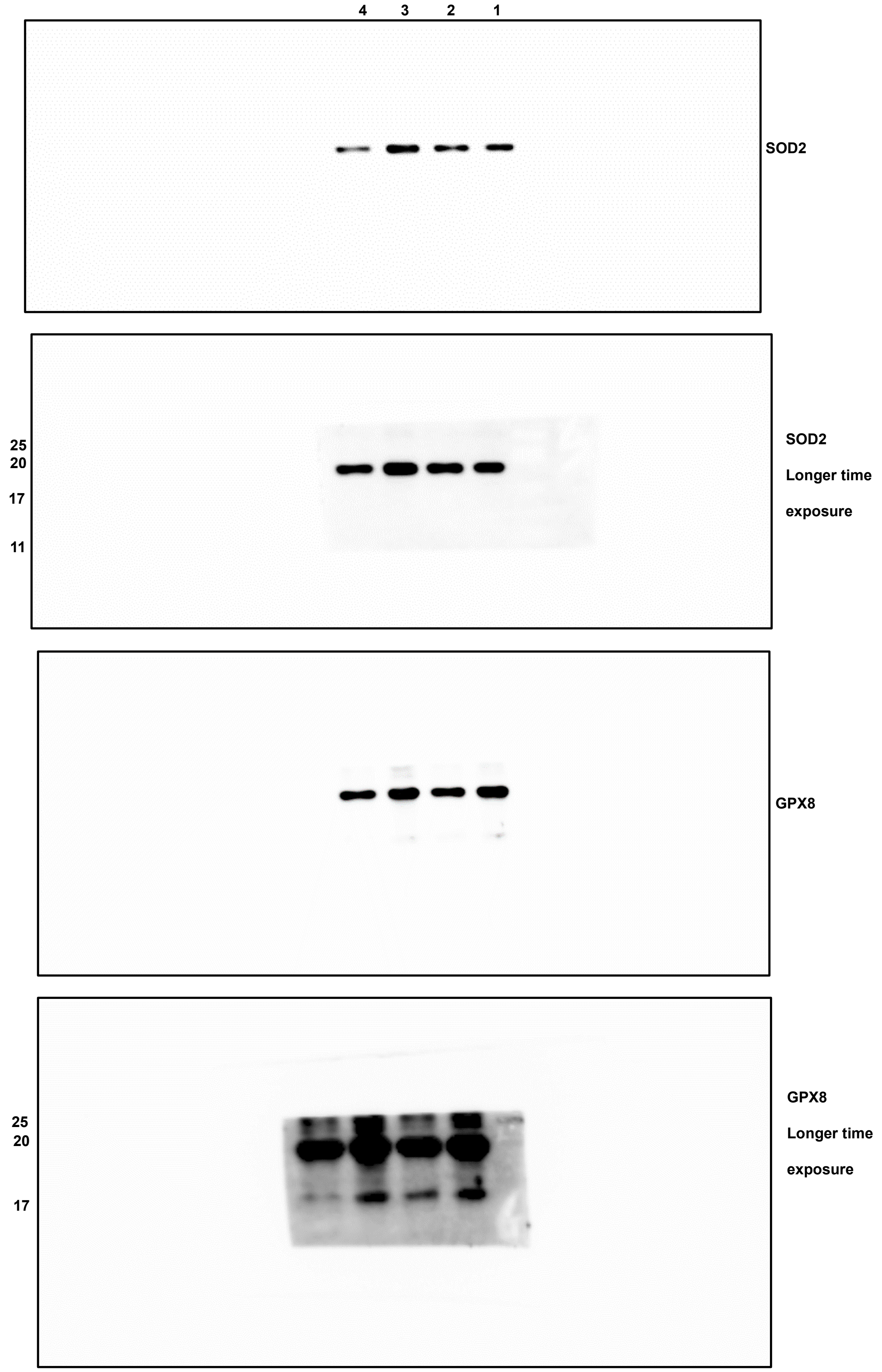

1.MDA-MB-231 control

2.TP-0903 0,25 μM

3.ART 40 μM

4.TP0903 0,25 μM

+ ART 40 μM

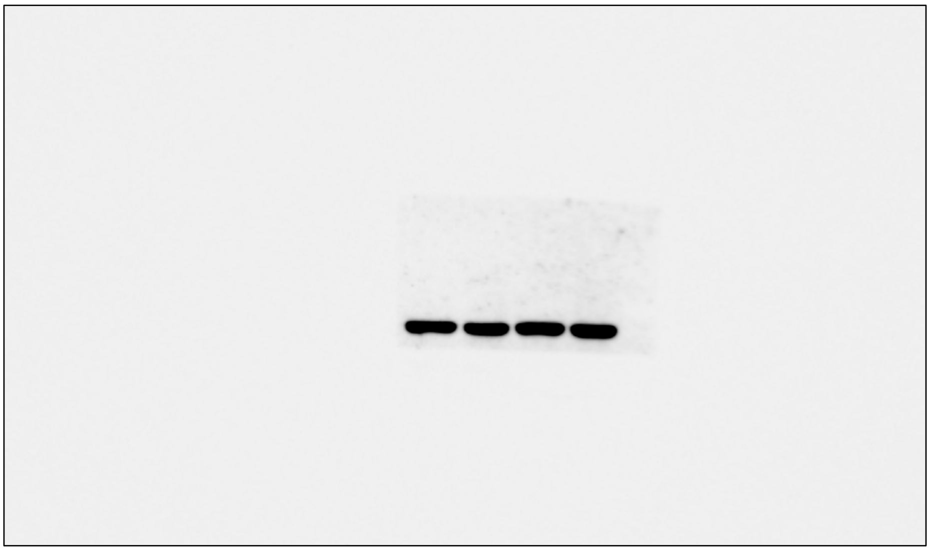

α-tubulin

Original Western Blot files for Figure S4

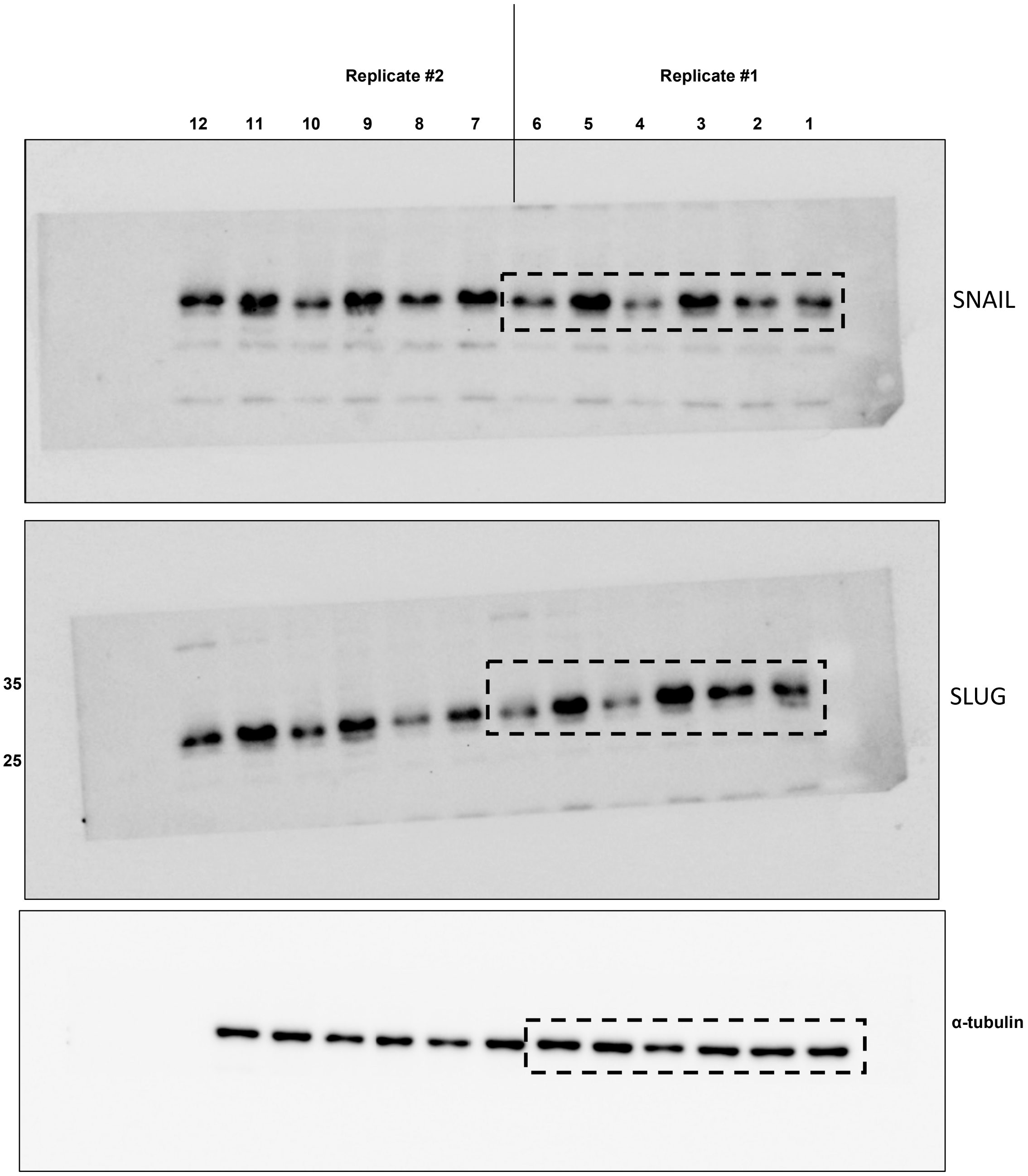

1. MDA-MB-231 cntrl 1 h
2. TP-0903 1 µM 1 h
3. MDA-MB-231 cntrl 4 h
4. TP-0903 1 µM 4 h
5. MDA-MB-231 cntrl 24 h
8. TP-0903 1 µM 24 h
7. MDA-MB-231 cntrl 1 h
8. TP-0903 1 µM 1 h
9. MDA-MB-231 cntrl 4 h
10. TP-0903 1 µM 4 h
11. MDA-MB-231 cntrl 24 h
12. TP-0903 1 µM 24 h
