# Supplementary material for: Mesenchymal–epithelial transition and AXL inhibitor TP-0903 sensitise triple-negative breast cancer cells to the antimalarial compound, artesunate
Source: Sci Rep. 2024 Jan 3;14:425. doi: 10.1038/s41598-023-50710-3 (PMC10764797; doi:10.1038/s41598-023-50710-3)
Supplement: Supplementary file 3 — Supplementary Information 2. [file 41598_2023_50710_MOESM3_ESM.pdf]

Title: Mesenchymal-epithelial transition and AXL inhibitor TP-0903 sensitise triple-negative breast cancer cells to the antimalarial compound, artesunate.

Mirko Terragno, Anastassiya Vetrova, Oleg Semenov, A. Emre Sayan, Marina Kriajevska, Eugene Tulchinsky

Supporting information 2 file - TP-0903/ART synergism in Compusyn

TP-0903 and ART synergism. Compusyn report of simultaneous (pages 2-23) and sequential (pages 24-45) treatment of MDA-MB-231 cells with TP-0903, ART and a combination of the two at different concentrations. The effects on cell viability were calculated by MTT assay before checking synergism in the Compusyn program. TP-0903 mainly synergized with the highest ART concentrations in a simultaneous treatment while in a sequential treatment, TP-0903 also synergised with the lowest ART concentrations. TP-0903 at 0,25 µM synergised with all ART concentrations as all CI values were lower than 1. However, CI values related to TP-0903 at 0,25 µM plus ART were lower in the sequential than the simultaneous treatment. Dose A= ART; Dose T= TP-0903; Effect= growth inhibition; CI= Combination Index. CI < 1 indicates synergism. MTT results are expressed as mean ± SEM of four technical replicates.

CompuSyn Report

Experiment Name: Artesunate and TP-0903 simultaneous combination
Date:
File Name: C:\Users\MIRKO TERRAGNO\Desktop\MTT\Combin\Low-report\Low.cse
Description Artesunate and TP-0903 simultaneous combination

Drug: Artesunate (A) [uM]
Drug: TP-0903 (T) [uM]
Drug Combo: A 2.5 - T (AT2.5) (A+T)
Drug Combo: A 5 - T (AT5) (A+T)
Drug Combo: A 10 - T (AT 10) (A+T)
Drug Combo: A 20 - T (AT20) (A+T)
Drug Combo: A 40 - T (AT40) (A+T)
Drug Combo: A 80 - T (AT80) (A+T)
Drug Combo: A 160 - T (AT160) (A+T)
Drug Combo: A 320 - T (AT320) (A+T)

Data for Drug: A [uM]

Dose	Effect
2.5	0.05851
5.0	0.15823
10.0	0.22803
20.0	0.25306
40.0	0.34826
80.0	0.48580
160.0	0.65513
320.0	0.91872

8 data points entered.
X-int: 1.71742
Y-int: -1.5318 +/- 0.17411
m: 0.89193 +/- 0.10834
Dm: 52.1694
r: 0.95848

Data for Drug: T [uM]

Dose	Effect
0.015	0.24139
0.03	0.51025
0.06	0.62126
0.125	0.63217
0.25	0.88448
0.5	0.90555
1.0	0.88053
2.0	0.90574

8 data points entered.
X-int: -1.4296

**Y-int:**	0.98273 +/- 0.11233
**m:**	0.68744 +/- 0.10899
**Dm:**	0.03719
**r:**	0.93217

Data for Non-Constant Combo: AT2.5 (A+T)

Dose A	Dose T	Effect
2.5	0.015	0.26631
2.5	0.03	0.43044
2.5	0.06	0.49427
2.5	0.125	0.57120
2.5	0.25	0.87795
2.5	0.5	0.88941
2.5	1.0	0.87234
2.5	2.0	0.87141

8 data points entered.

Data for Non-Constant Combo: AT5 (A+T)

Dose A	Dose T	Effect
5.0	0.015	0.22703
5.0	0.03	0.44985
5.0	0.06	0.55156
5.0	0.125	0.57821
5.0	0.25	0.85784
5.0	0.5	0.89876
5.0	1.0	0.91373
5.0	2.0	0.92167

8 data points entered.

Data for Non-Constant Combo: AT 10 (A+T)

Dose A	Dose T	Effect
10.0	0.015	0.31449
10.0	0.03	0.44755
10.0	0.06	0.52150
10.0	0.125	0.59243
10.0	0.25	0.82739
10.0	0.5	0.83684
9.0	1.0	0.88973
10.0	2.0	0.90821

8 data points entered.

Data for Non-Constant Combo: AT20 (A+T)

Dose A	Dose T	Effect
20.0	0.015	0.26956
20.0	0.03	0.45636
20.0	0.06	0.56298
20.0	0.125	0.60727
20.0	0.25	0.84351

20.0	0.5	0.88543
20.0	1.0	0.87059
20.0	2.0	0.91359

8 data points entered.

Data for Non-Constant Combo: AT40 (A+T)

**Dose A**	**Dose T**	**Effect**
40.0	0.015	0.46919
40.0	0.03	0.50099
40.0	0.06	0.59841
40.0	0.125	0.65341
40.0	0.25	0.84449
40.0	0.5	0.91297
40.0	1.0	0.89530
40.0	2.0	0.93219

8 data points entered.

Data for Non-Constant Combo: AT80 (A+T)

**Dose A**	**Dose T**	**Effect**
80.0	0.015	0.51977
80.0	0.03	0.59708
80.0	0.06	0.63022
80.0	0.125	0.72344
80.0	0.25	0.88293
80.0	0.5	0.93992
80.0	1.0	0.96620
80.0	2.0	0.96841

8 data points entered.

Data for Non-Constant Combo: AT160 (A+T)

**Dose A**	**Dose T**	**Effect**
160.0	0.015	0.67847
160.0	0.03	0.69628
160.0	0.06	0.72655
160.0	0.125	0.96498
160.0	0.25	0.96122
160.0	0.5	0.95983
148.0	1.0	0.90507
160.0	2.0	0.97428

8 data points entered.

Data for Non-Constant Combo: AT320 (A+T)

**Dose A**	**Dose T**	**Effect**
320.0	0.015	0.90186
320.0	0.03	0.87099
320.0	0.06	0.92066
320.0	0.125	0.96498
320.0	0.25	0.96122

320.0	0.5	0.95983
320.0	1.0	0.97448
320.0	2.0	0.96775

8 data points entered.

Dose-Effect Curve for Drugs

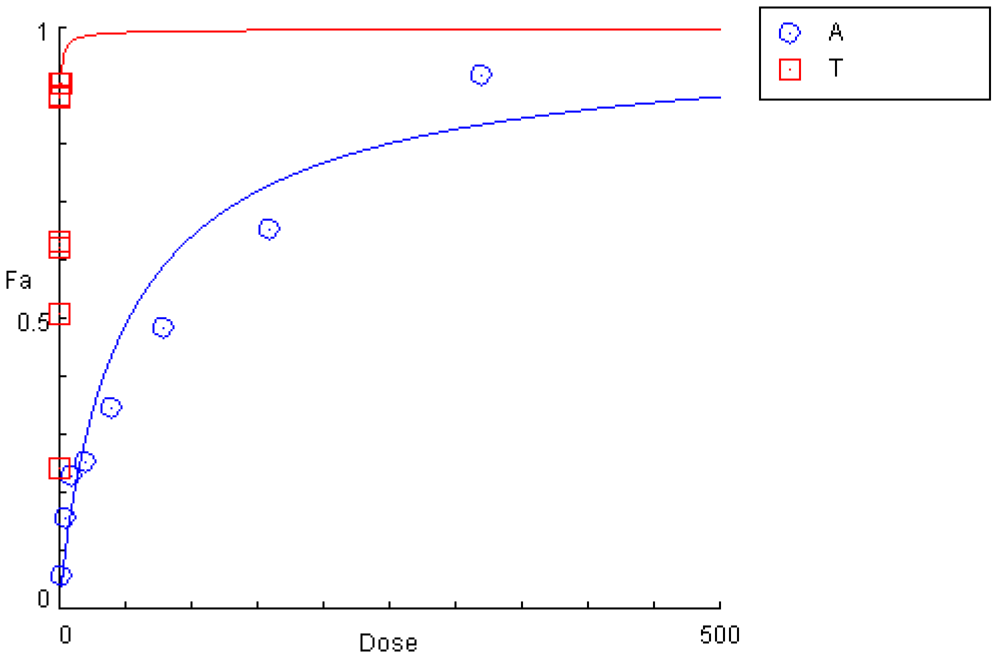

Dose-Effect Curve for Drug Combos

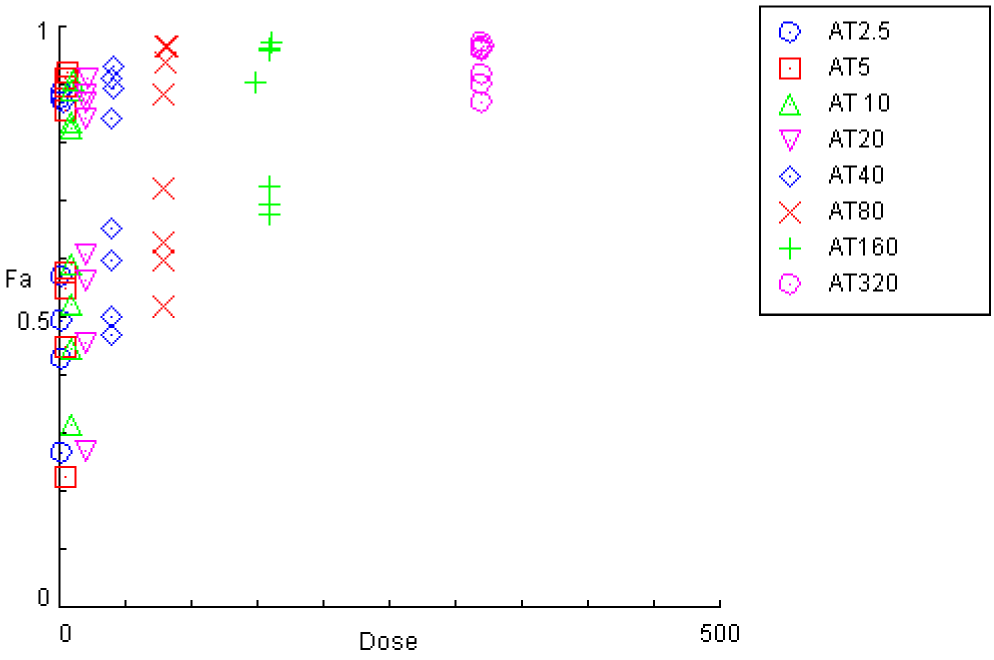

Median-Effect Plot for Drugs

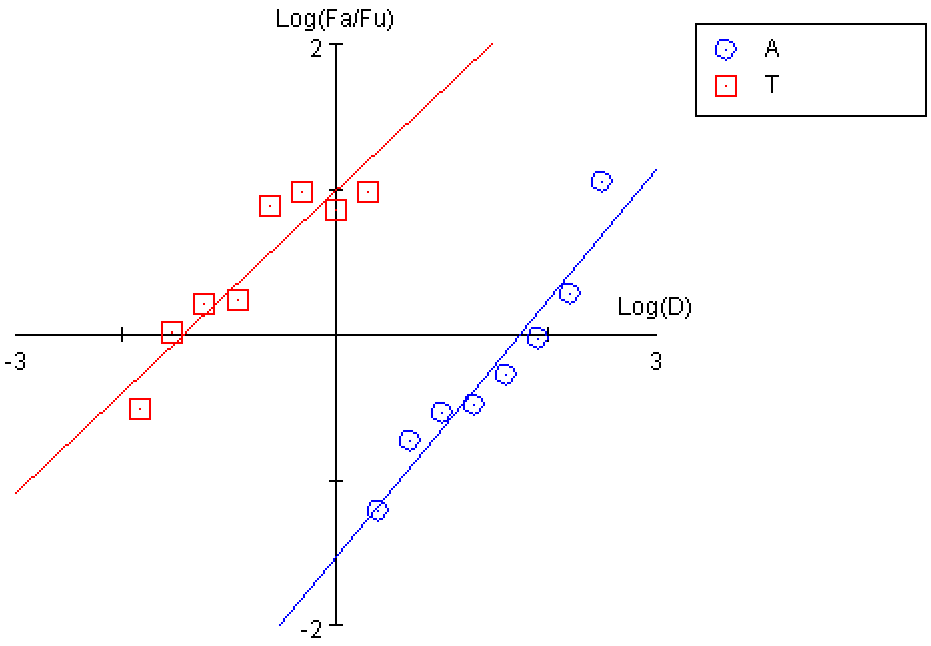

Median-Effect Plot for Drug Combos

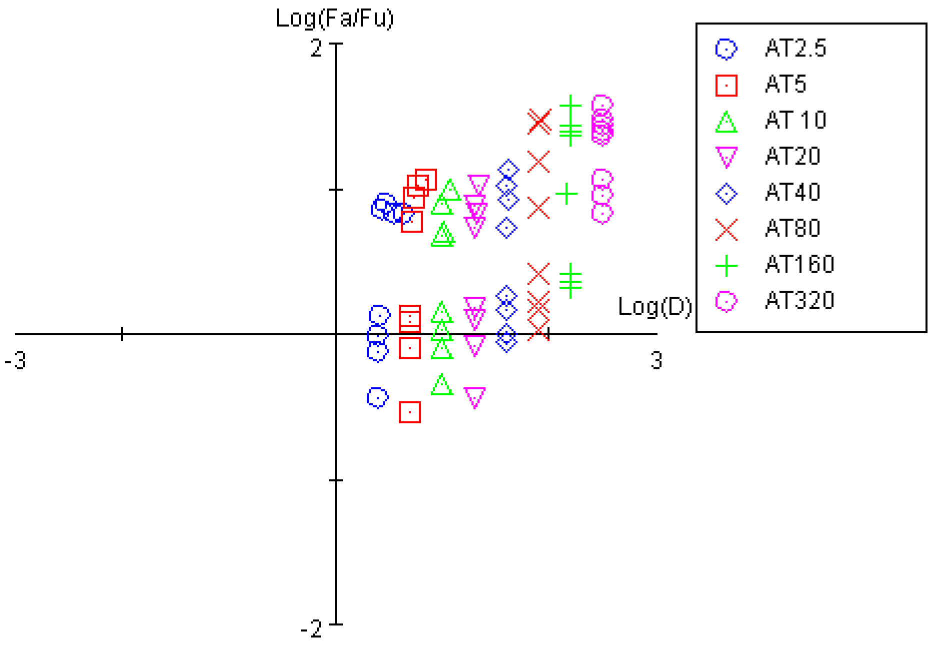

CI Data for Non-Constant Combo: AT2.5 (A+T)

Dose A	Dose T	Effect	CI
2.5	0.015	0.26631	1.91085
2.5	0.03	0.43044	1.27789
2.5	0.06	0.49427	1.71714
2.5	0.125	0.57120	2.24952
2.5	0.25	0.87795	0.38625
2.5	0.5	0.88941	0.65251
2.5	1.0	0.87234	1.64781
2.5	2.0	0.87141	3.33035

CI Data for Non-Constant Combo: AT5 (A+T)

Dose A	Dose T	Effect	CI

5.0	0.015	0.22703	2.77553
5.0	0.03	0.44985	1.20116
5.0	0.06	0.55156	1.26989
5.0	0.125	0.57821	2.19151
5.0	0.25	0.85784	0.50472
5.0	0.5	0.89876	0.56942
5.0	1.0	0.91373	0.87499
5.0	2.0	0.92167	1.49577

CI Data for Non-Constant Combo: AT 10 (A+T)

Dose A	Dose T	Effect	CI
10.0	0.015	0.31449	1.71215
10.0	0.03	0.44755	1.33850
10.0	0.06	0.52150	1.59756
10.0	0.125	0.59243	2.07665
10.0	0.25	0.82739	0.72077
10.0	0.5	0.83684	1.27703
9.0	1.0	0.88973	1.30630
10.0	2.0	0.90821	1.93158

CI Data for Non-Constant Combo: AT20 (A+T)

Dose A	Dose T	Effect	CI
20.0	0.015	0.26956	2.89177
20.0	0.03	0.45636	1.50696
20.0	0.06	0.56298	1.40469
20.0	0.125	0.60727	2.01809
20.0	0.25	0.84351	0.63777
20.0	0.5	0.88543	0.72528
20.0	1.0	0.87059	1.72517
20.0	2.0	0.91359	1.76808

CI Data for Non-Constant Combo: AT40 (A+T)

Dose A	Dose T	Effect	CI
40.0	0.015	0.46919	1.36316
40.0	0.03	0.50099	1.56532
40.0	0.06	0.59841	1.39338
40.0	0.125	0.65341	1.71287
40.0	0.25	0.84449	0.68855
40.0	0.5	0.91297	0.49516
40.0	1.0	0.89530	1.25436
40.0	2.0	0.93219	1.22885

CI Data for Non-Constant Combo: AT80 (A+T)

Dose A	Dose T	Effect	CI
80.0	0.015	0.51977	1.76277
80.0	0.03	0.59708	1.44185
80.0	0.06	0.63022	1.58630

80.0	0.125	0.72344	1.35158
80.0	0.25	0.88293	0.51488
80.0	0.5	0.93992	0.31638
80.0	1.0	0.96620	0.24050
80.0	2.0	0.96841	0.40300

CI Data for Non-Constant Combo: AT160 (A+T)

**Dose A**	**Dose T**	**Effect**	**CI**
160.0	0.015	0.67847	1.46379
160.0	0.03	0.69628	1.45116
160.0	0.06	0.72655	1.41475
160.0	0.125	0.96498	0.10149
160.0	0.25	0.96122	0.14687
160.0	0.5	0.95983	0.22026
148.0	1.0	0.90507	1.23812
160.0	2.0	0.97428	0.32412

CI Data for Non-Constant Combo: AT320 (A+T)

**Dose A**	**Dose T**	**Effect**	**CI**
320.0	0.015	0.90186	0.52619
320.0	0.03	0.87099	0.77098
320.0	0.06	0.92066	0.43840
320.0	0.125	0.96498	0.17596
320.0	0.25	0.96122	0.23073
320.0	0.5	0.95983	0.30763
320.0	1.0	0.97448	0.23777
320.0	2.0	0.96775	0.51708

Combination Index Plot

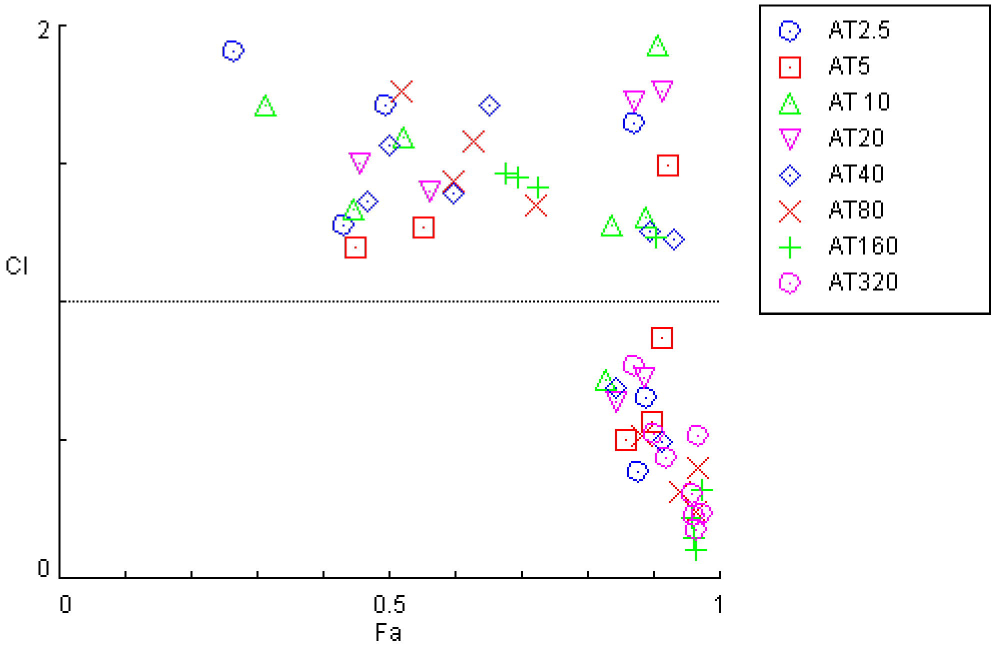

Logarithmic Combination Index Plot

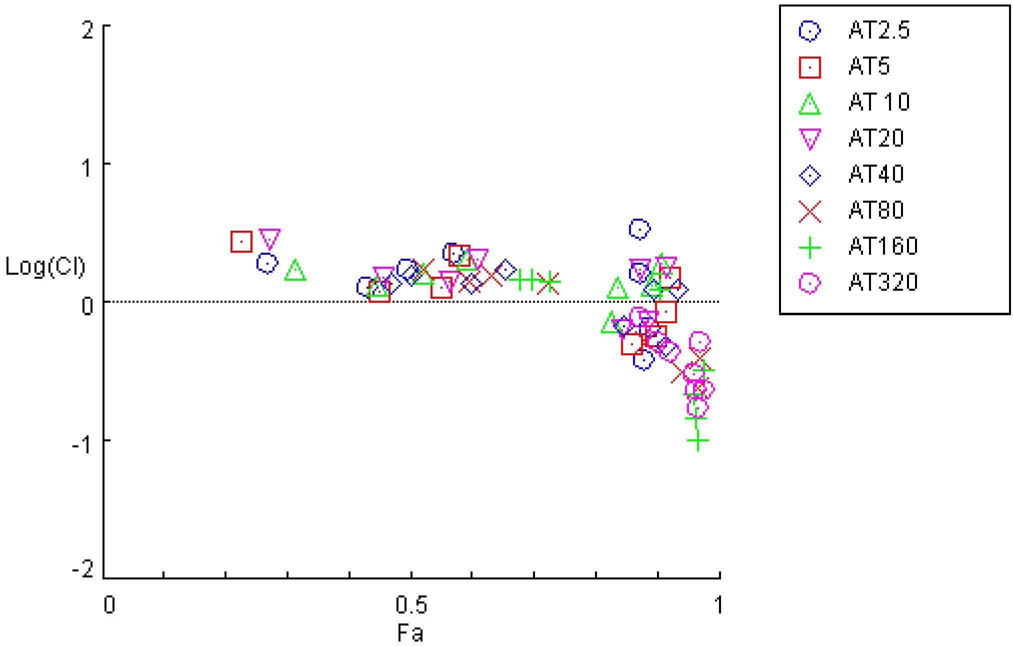

DRI Data for Non-Constant Combo: AT2.5 (A+T)

Fa	Dose A	Dose T	DRI A	DRI T
0.26631	16.7478	0.00852	6.69913	0.56767
0.43044	38.1115	0.02475	15.2446	0.82488
0.49427	50.8463	0.03597	20.3385	0.59953
0.57120	71.9496	0.05644	28.7798	0.45151
0.87795	476.636	0.65616	190.654	2.62466
0.88941	540.123	0.77174	216.049	1.54349
0.87234	449.961	0.60892	179.984	0.60892
0.87141	445.758	0.60155	178.303	0.30078

DRI Data for Non-Constant Combo: AT5 (A+T)

Fa	Dose A	Dose T	DRI A	DRI T
0.22703	13.2088	0.00626	2.64175	0.41719
0.44985	41.6301	0.02775	8.32602	0.92502
0.55156	65.7939	0.05026	13.1588	0.83759
0.57821	74.3022	0.05885	14.8604	0.47076
0.85784	391.422	0.50819	78.2844	2.03276
0.89876	603.410	0.89106	120.682	1.78212
0.91373	735.414	1.15182	147.083	1.15182
0.92167	827.589	1.34253	165.518	0.67126

DRI Data for Non-Constant Combo: AT 10 (A+T)

Fa	Dose A	Dose T	DRI A	DRI T
0.31449	21.7772	0.01197	2.17772	0.79812
0.44755	41.1985	0.02738	4.11985	0.91260
0.52150	57.4525	0.04215	5.74525	0.70249
0.59243	79.3484	0.06408	7.93484	0.51266
0.82739	302.355	0.36353	30.2355	1.45412
0.83684	326.204	0.40116	32.6204	0.80233

0.88973	542.081	0.77538	60.2312	0.77538
0.90821	681.435	1.04335	68.1435	0.52167

DRI Data for Non-Constant Combo: AT20 (A+T)

Fa	Dose A	Dose T	DRI A	DRI T
0.26956	17.0621	0.00872	0.85311	0.58153
0.45636	42.8755	0.02883	2.14377	0.96109
0.56298	69.3015	0.05376	3.46507	0.89598
0.60727	85.0414	0.07011	4.25207	0.56088
0.84351	344.871	0.43120	17.2435	1.72480
0.88543	516.517	0.72827	25.8258	1.45654
0.87059	442.161	0.59526	22.1080	0.59526
0.91359	733.962	1.14887	36.6981	0.57444

DRI Data for Non-Constant Combo: AT40 (A+T)

Fa	Dose A	Dose T	DRI A	DRI T
0.46919	45.4279	0.03108	1.13570	2.07194
0.50099	52.4025	0.03741	1.31006	1.24689
0.59841	81.5867	0.06644	2.03967	1.10729
0.65341	106.208	0.09355	2.65520	0.74836
0.84449	347.764	0.43590	8.69410	1.74360
0.91297	727.559	1.13589	18.1890	2.27177
0.89530	578.549	0.84372	14.4637	0.84372
0.93219	985.142	1.68315	24.6286	0.84157

DRI Data for Non-Constant Combo: AT80 (A+T)

Fa	Dose A	Dose T	DRI A	DRI T
0.51977	57.0087	0.04173	0.71261	2.78184
0.59708	81.0823	0.06590	1.01353	2.19683
0.63022	94.8458	0.08077	1.18557	1.34621
0.72344	153.328	0.15063	1.91660	1.20507
0.88293	502.565	0.70285	6.28207	2.81140
0.93992	1138.82	2.03146	14.2353	4.06292
0.96620	2238.95	4.88350	27.9869	4.88350
0.96841	2421.42	5.40602	30.2677	2.70301

DRI Data for Non-Constant Combo: AT160 (A+T)

Fa	Dose A	Dose T	DRI A	DRI T
0.67847	120.510	0.11021	0.75319	7.34715
0.69628	132.246	0.12433	0.82654	4.14432
0.72655	156.038	0.15410	0.97524	2.56829
0.96498	2148.28	4.62846	13.4267	37.0277
0.96122	1907.86	3.96789	11.9241	15.8716
0.95983	1831.30	3.76253	11.4456	7.52507
0.90507	653.626	0.98844	4.41639	0.98844
0.97428	3069.29	7.35319	19.1830	3.67660

DRI Data for Non-Constant Combo: AT320 (A+T)

Fa	Dose A	Dose T	DRI A	DRI T
0.90186	627.229	0.93696	1.96009	62.4642
0.87099	443.927	0.59835	1.38727	19.9449
0.92066	814.696	1.31546	2.54593	21.9243
0.96498	2148.28	4.62846	6.71337	37.0277
0.96122	1907.86	3.96789	5.96207	15.8716
0.95983	1831.30	3.76253	5.72281	7.52507
0.97448	3096.70	7.43851	9.67719	7.43851
0.96775	2363.78	5.23965	7.38680	2.61982

DRI Plot for Non-Constant Combo: AT2.5 (A+T)

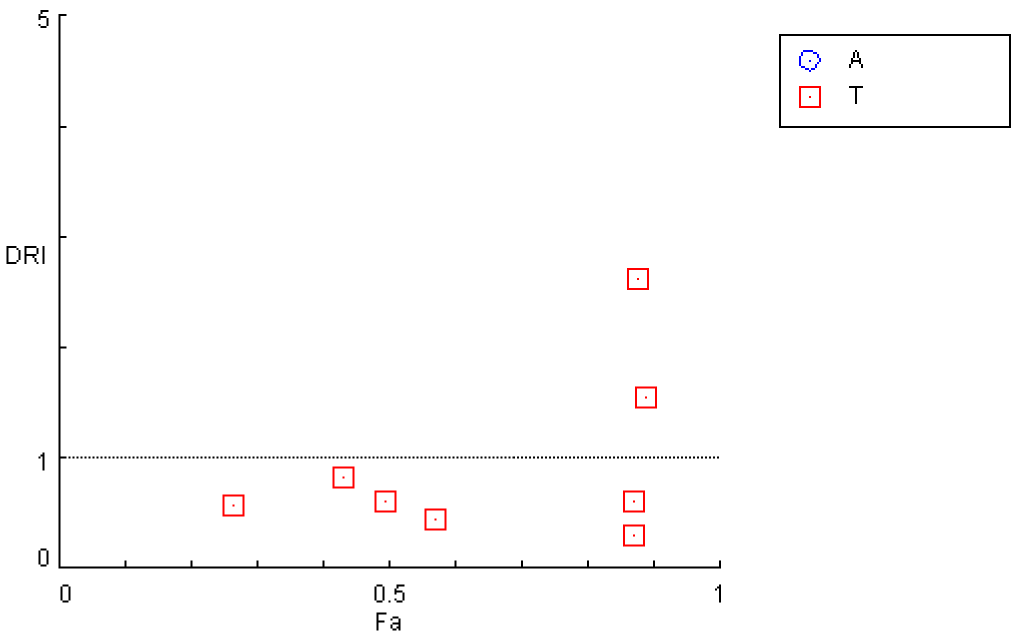

DRI Plot for Non-Constant Combo: AT5 (A+T)

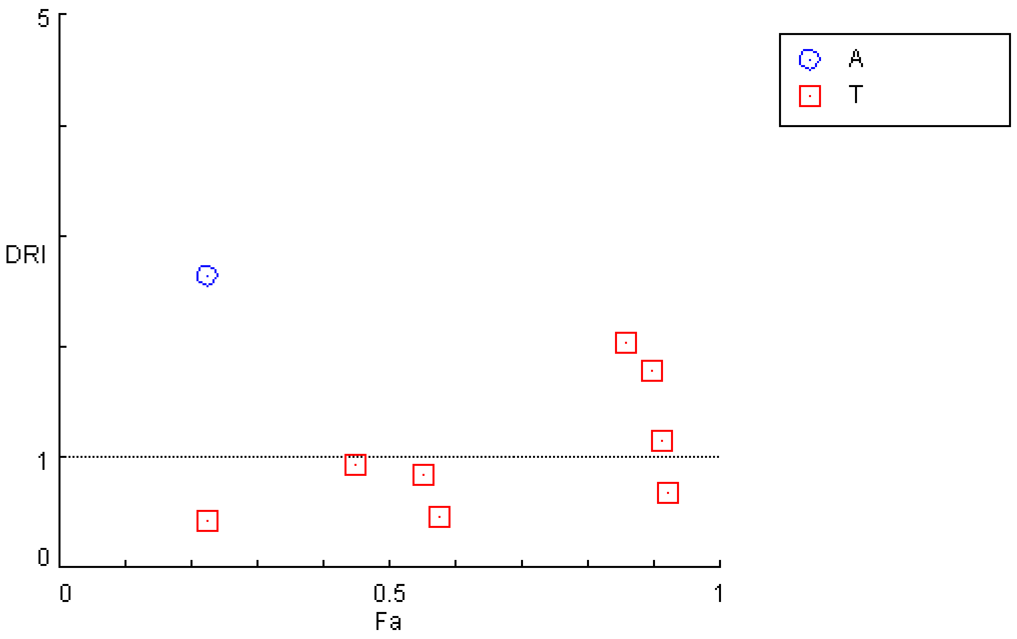

DRI Plot for Non-Constant Combo: AT 10 (A+T)

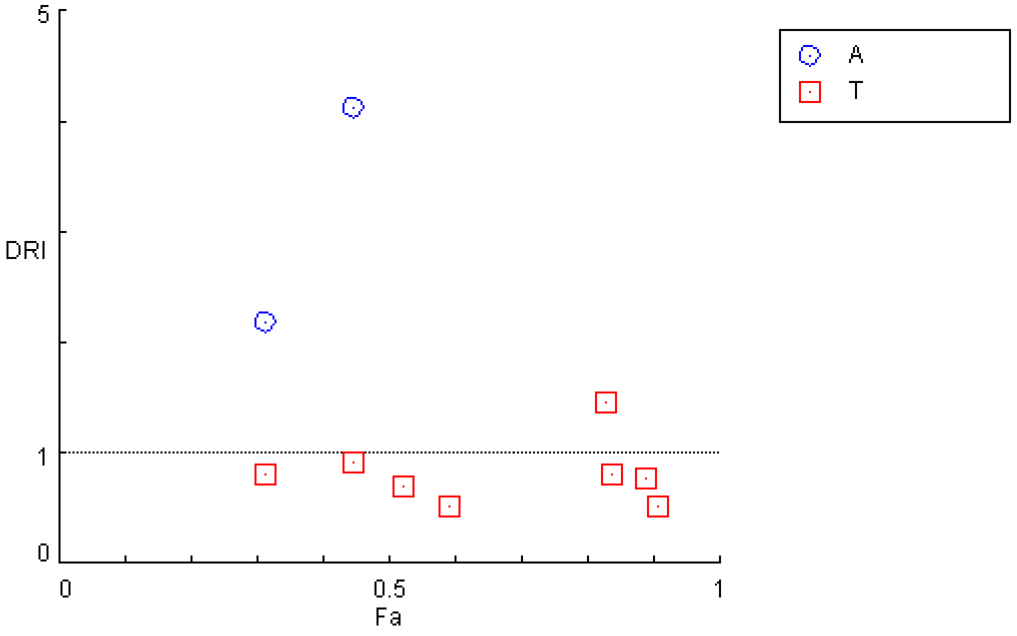

DRI Plot for Non-Constant Combo: AT20 (A+T)

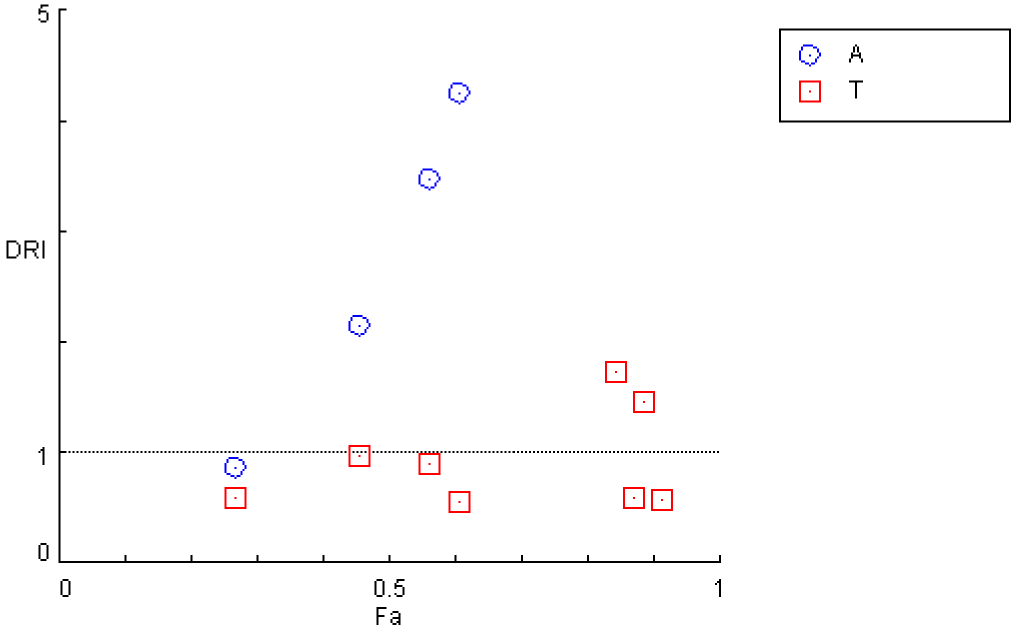

DRI Plot for Non-Constant Combo: AT40 (A+T)

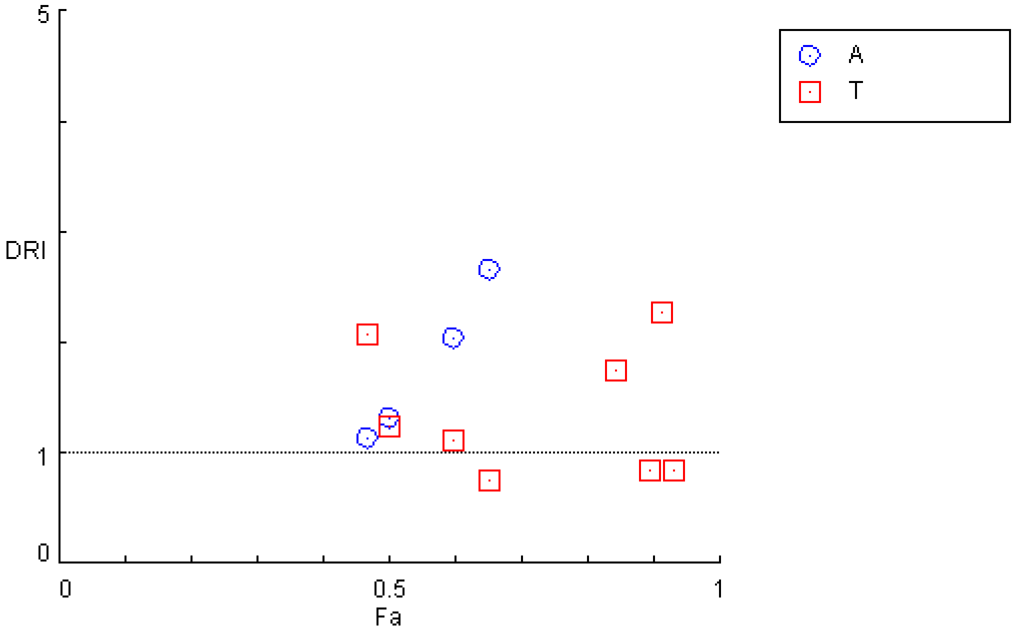

DRI Plot for Non-Constant Combo: AT80 (A+T)

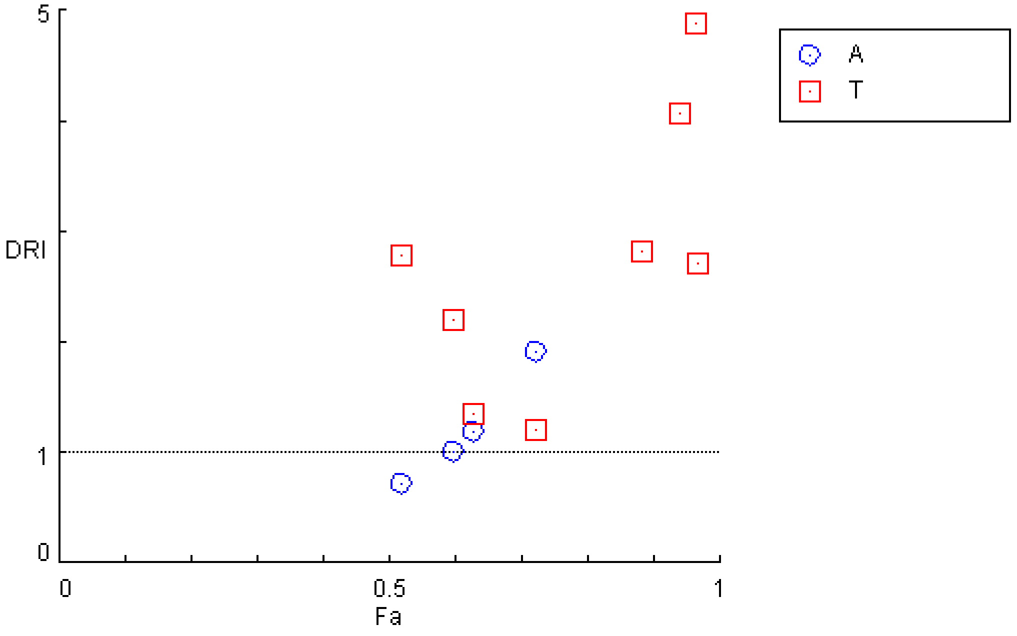

DRI Plot for Non-Constant Combo: AT160 (A+T)

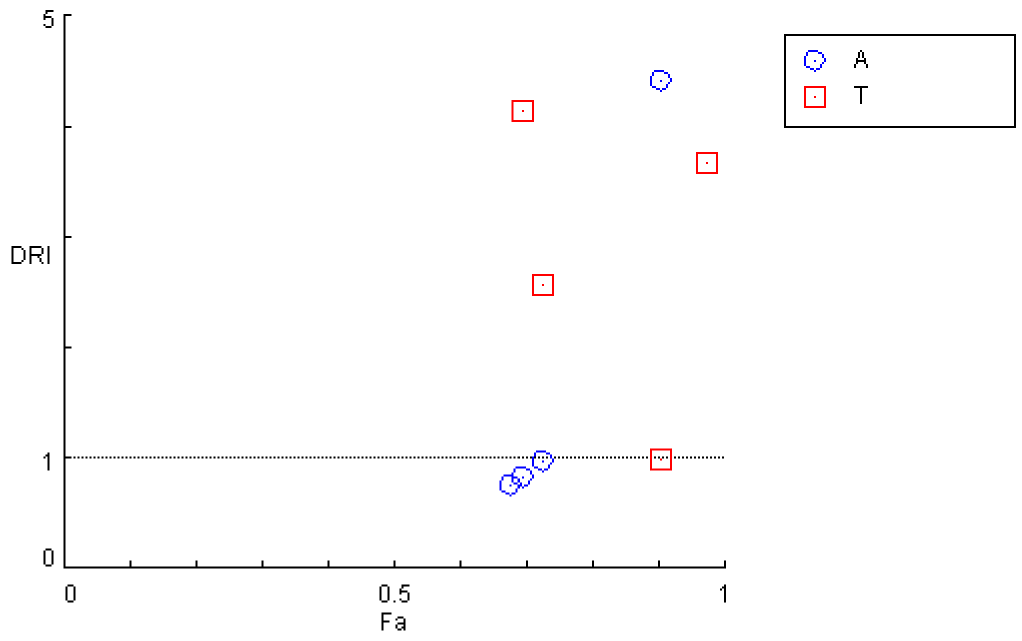

DRI Plot for Non-Constant Combo: AT320 (A+T)

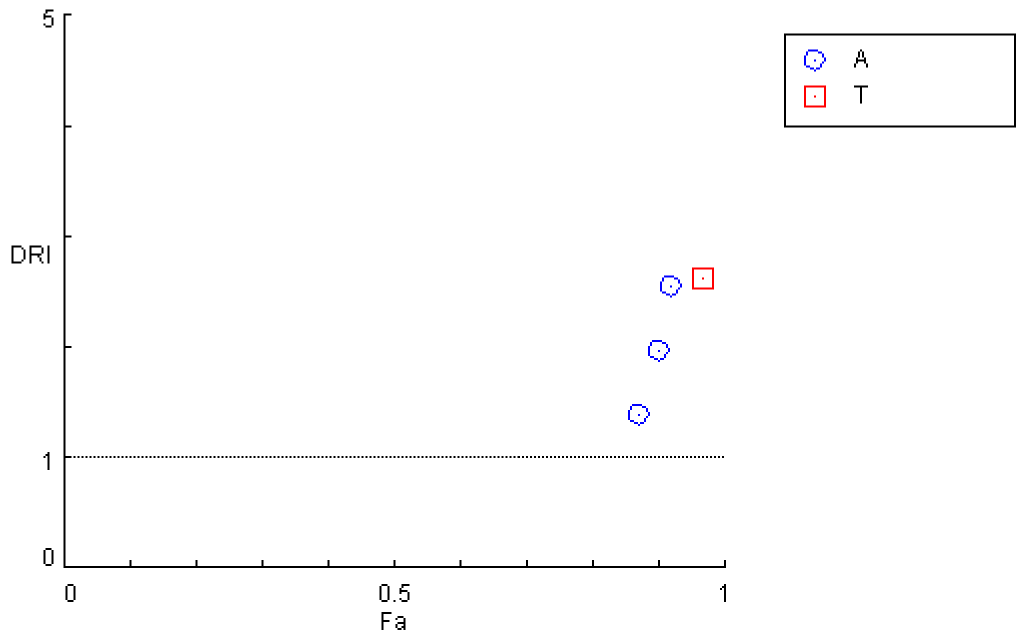

Log(DRI) Plot for Non-Constant Combo: AT2.5 (A+T)

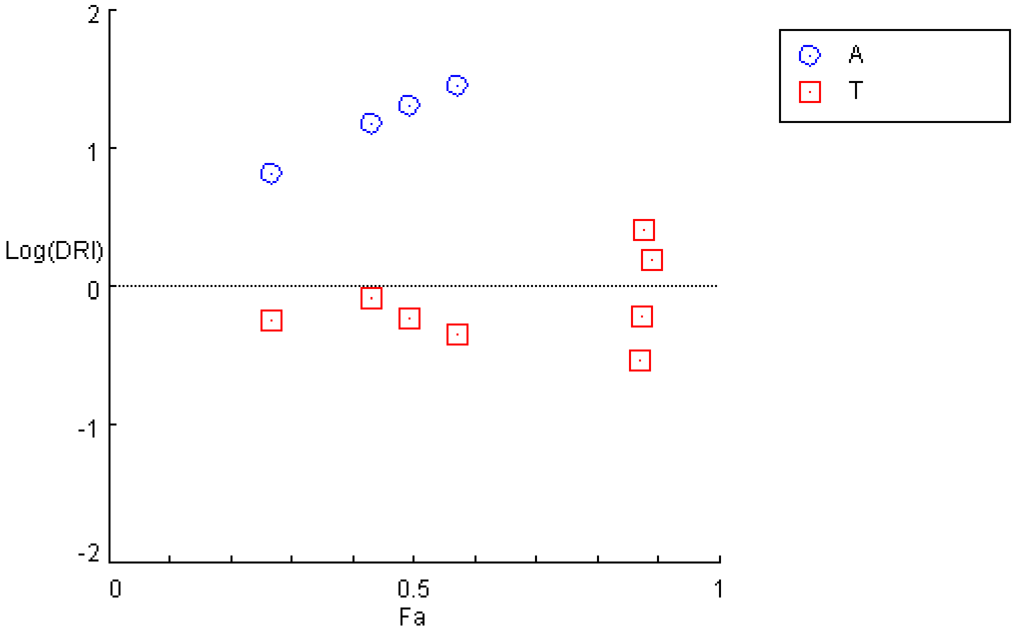

Log(DRI) Plot for Non-Constant Combo: AT5 (A+T)

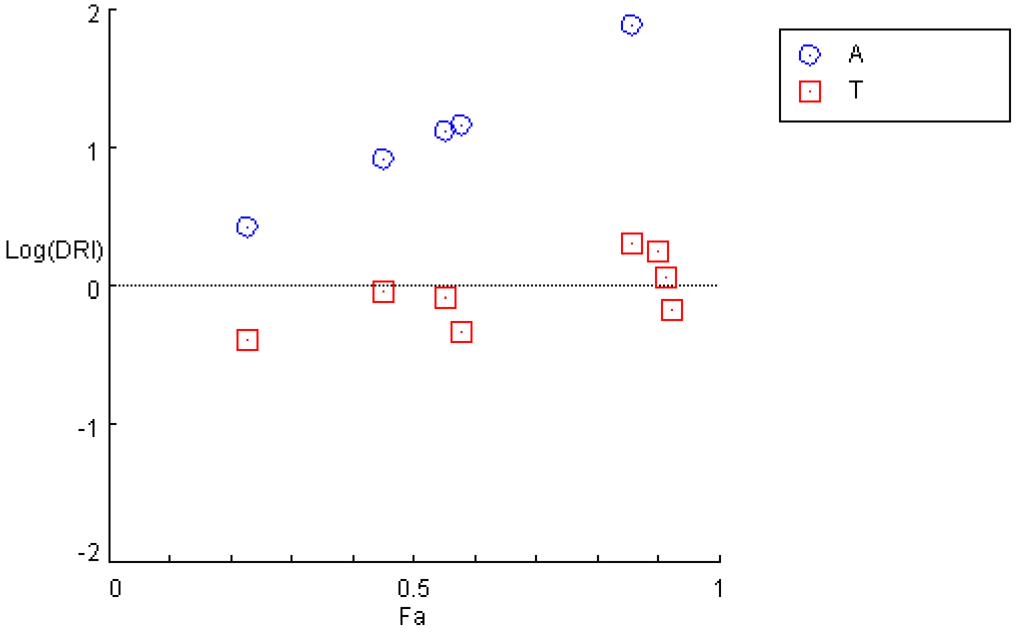

Log(DRI) Plot for Non-Constant Combo: AT 10 (A+T)

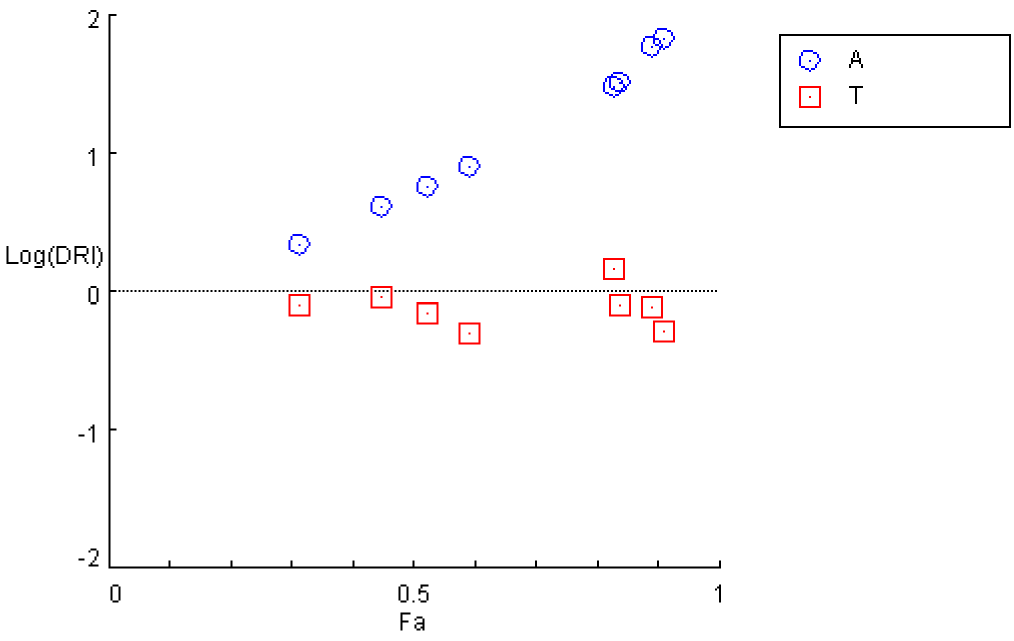

Log(DRI) Plot for Non-Constant Combo: AT20 (A+T)

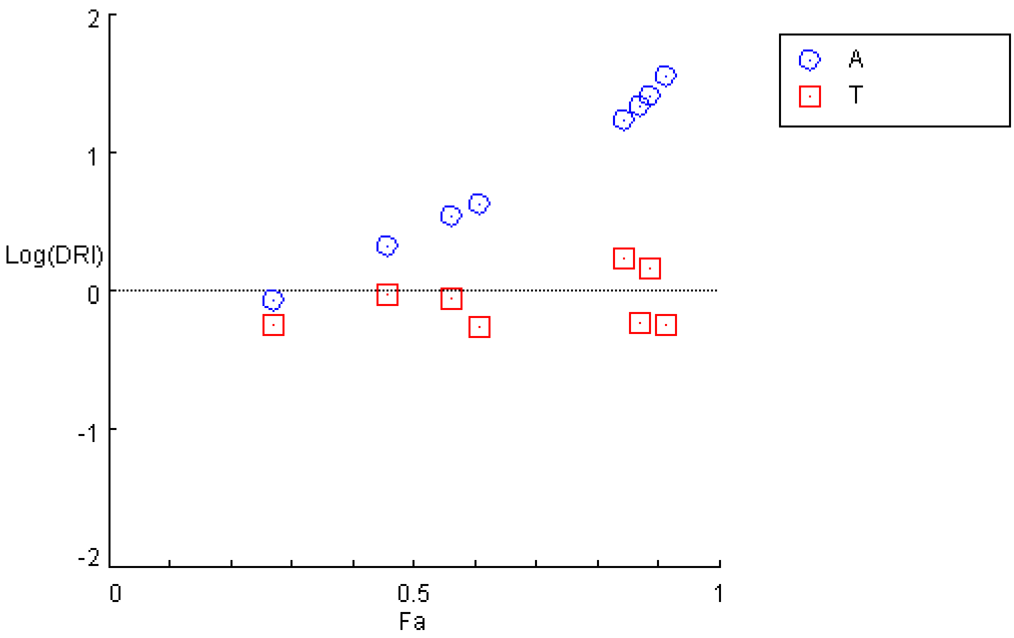

Log(DRI) Plot for Non-Constant Combo: AT40 (A+T)

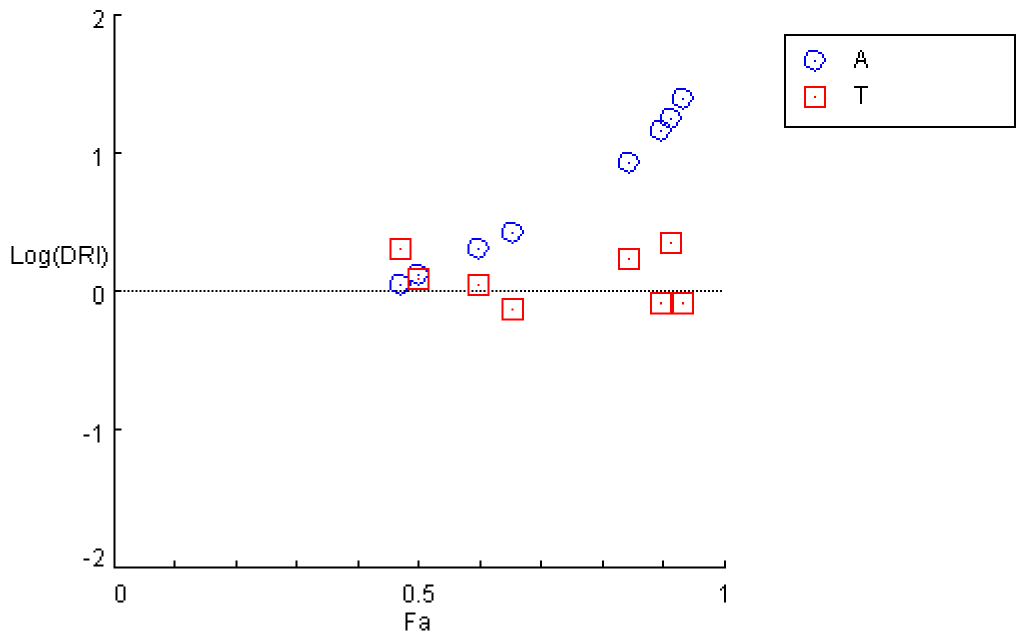

Log(DRI) Plot for Non-Constant Combo: AT80 (A+T)

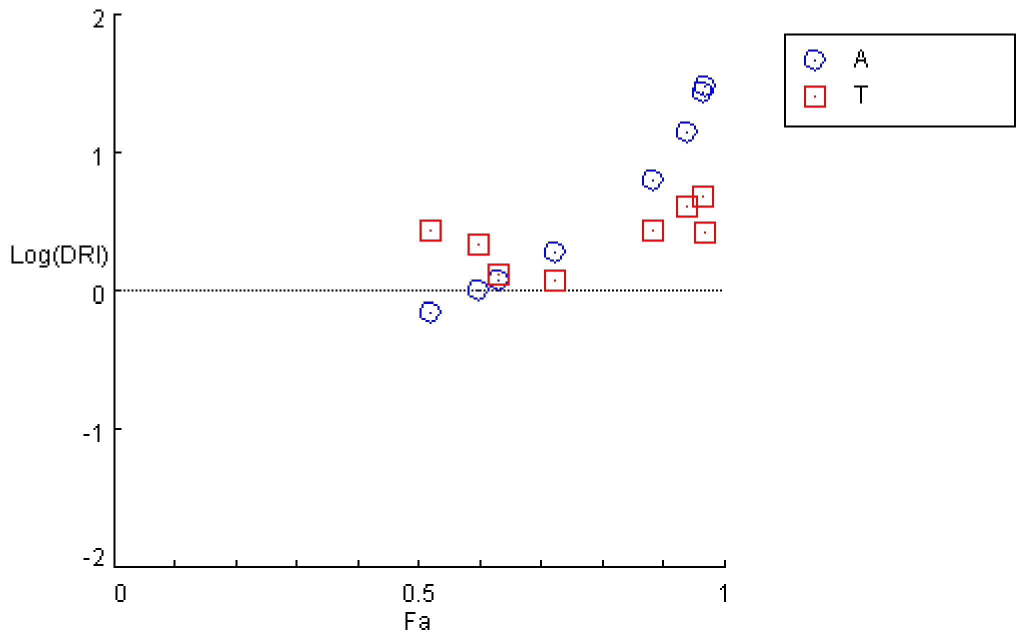

Log(DRI) Plot for Non-Constant Combo: AT160 (A+T)

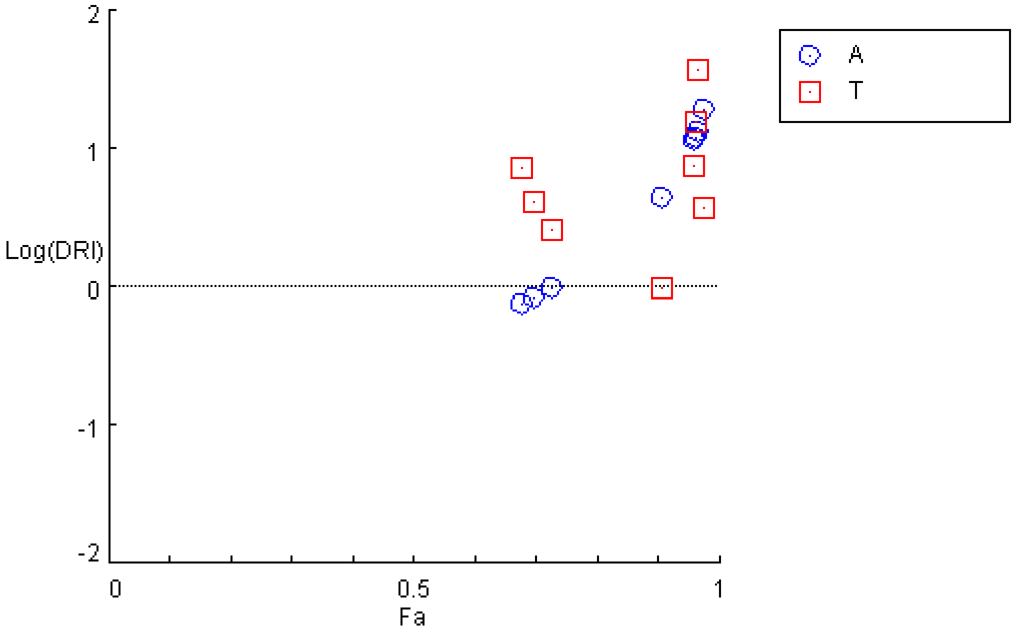

Log(DRI) Plot for Non-Constant Combo: AT320 (A+T)

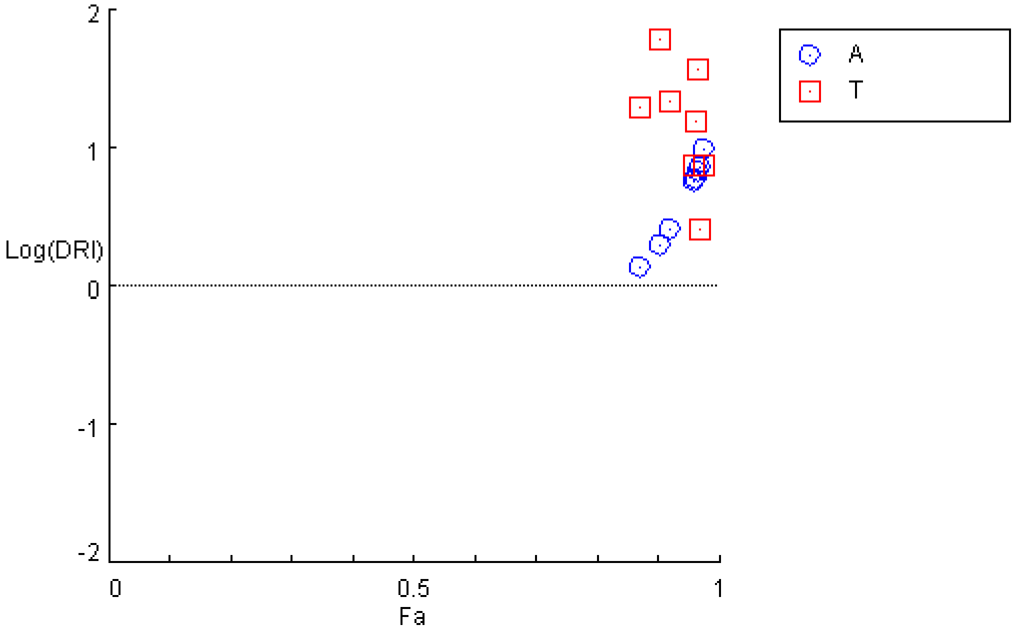

Normalized Isobologram for Combo: AT2.5 (A+T)

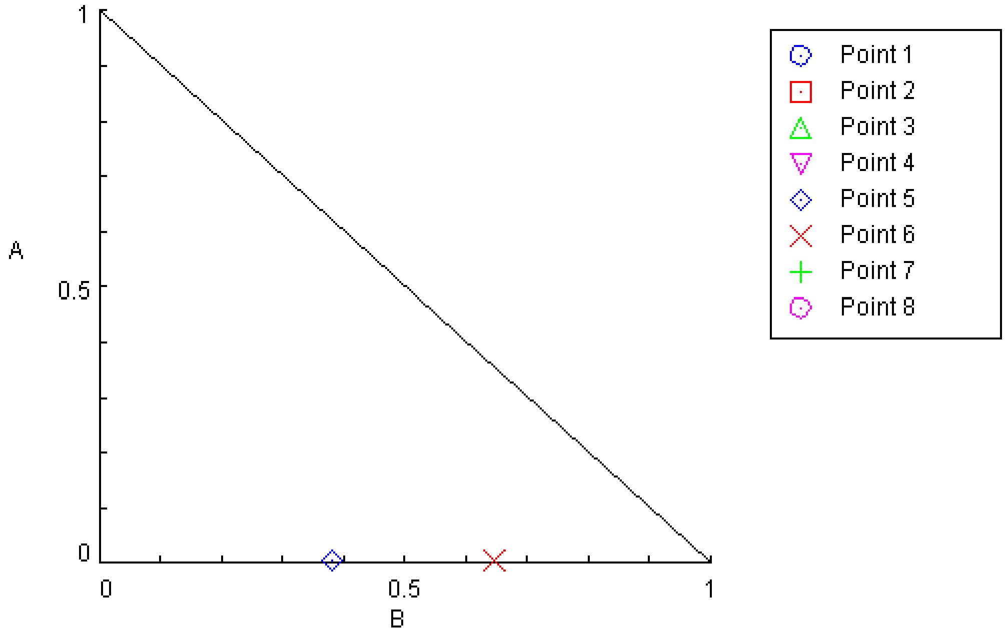

Normalized Isobologram for Combo: AT5 (A+T)

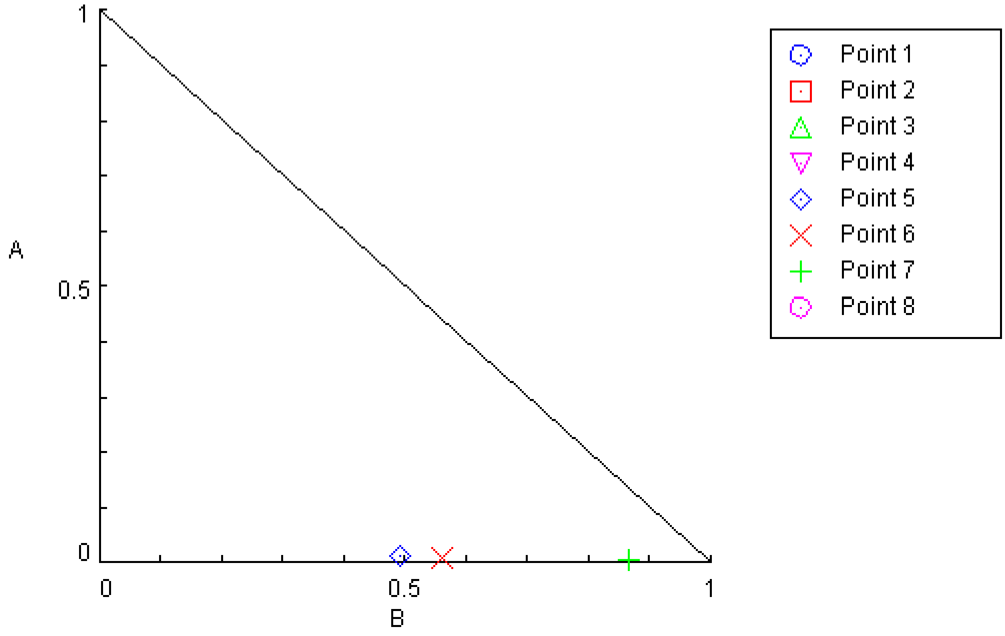

Normalized Isobologram for Combo: AT 10 (A+T)

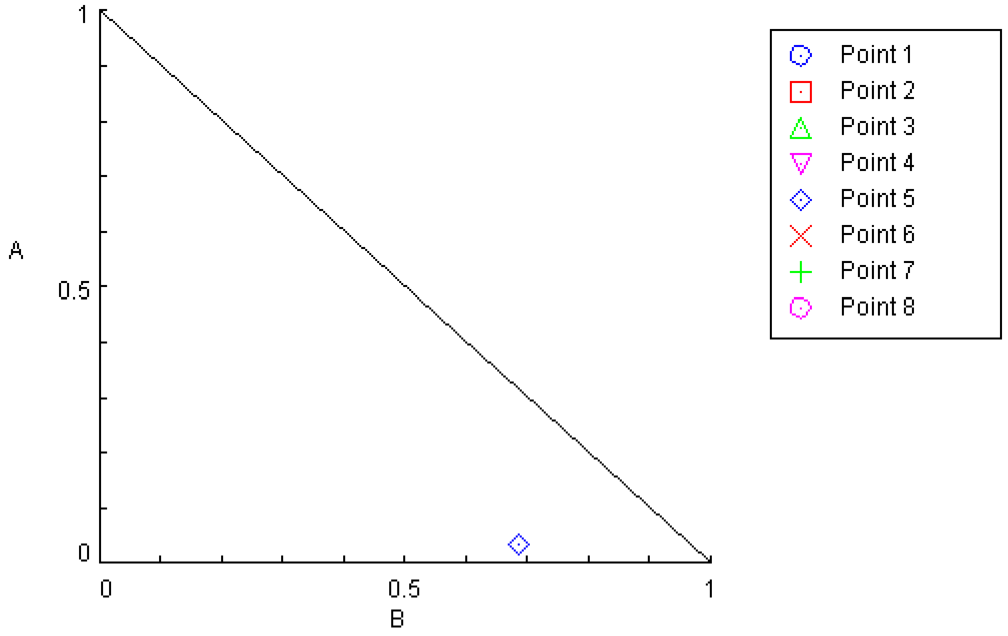

Normalized Isobologram for Combo: AT20 (A+T)

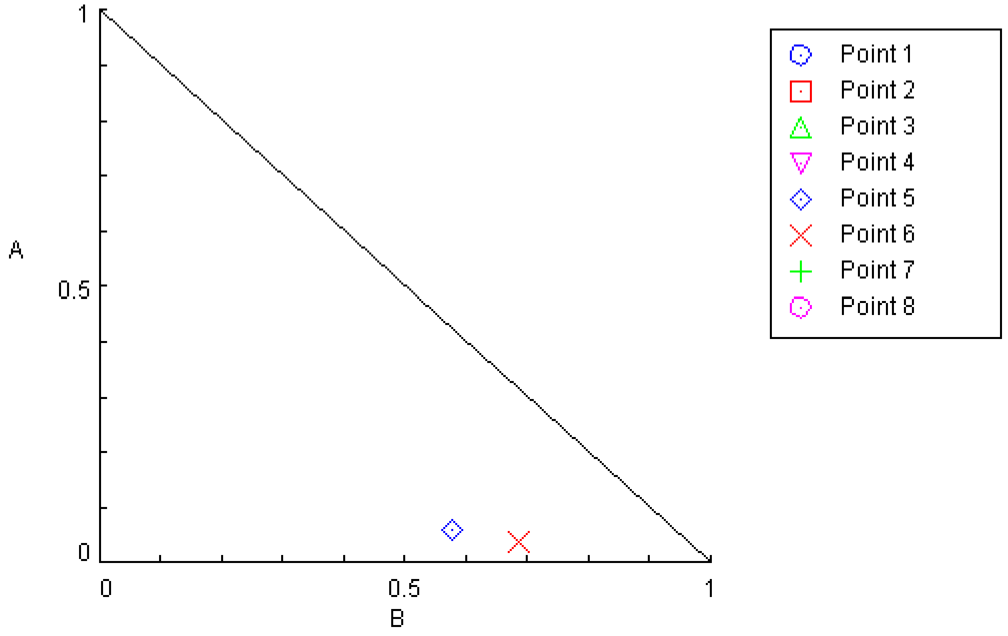

Normalized Isobologram for Combo: AT40 (A+T)

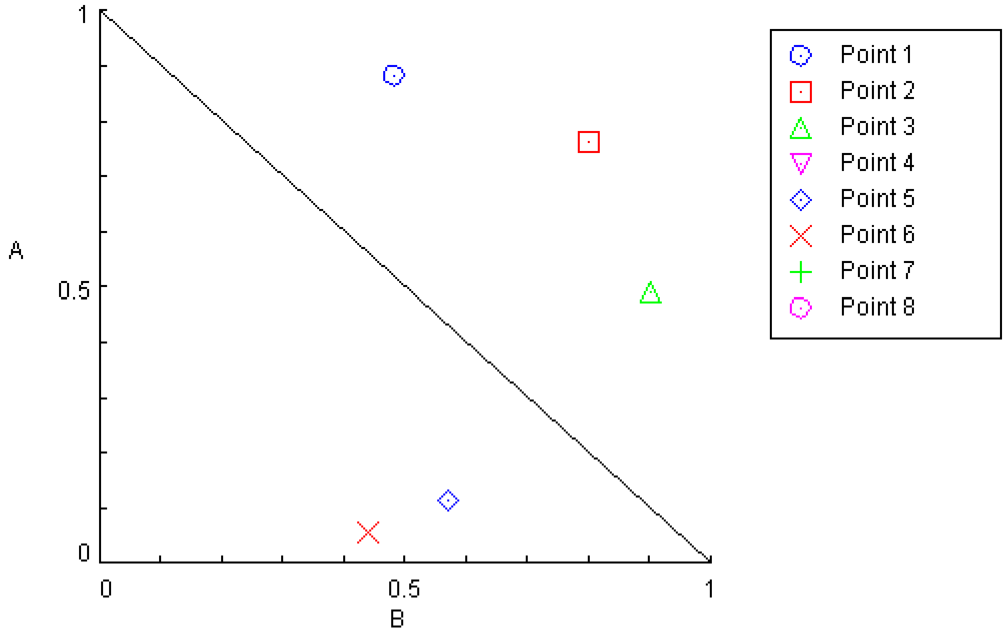

Normalized Isobologram for Combo: AT80 (A+T)

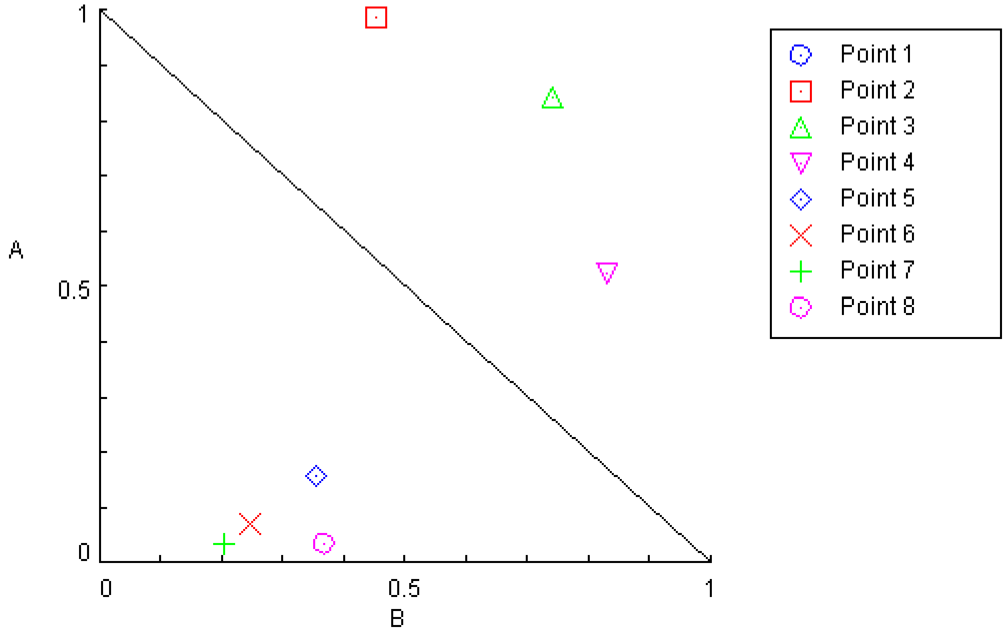

Normalized Isobologram for Combo: AT160 (A+T)

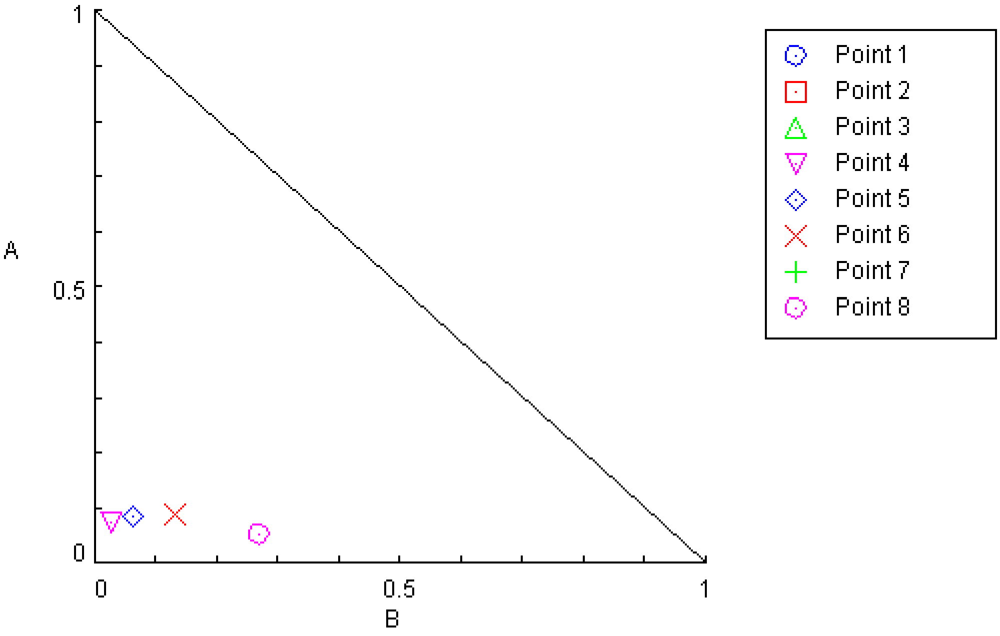

Normalized Isobologram for Combo: AT320 (A+T)

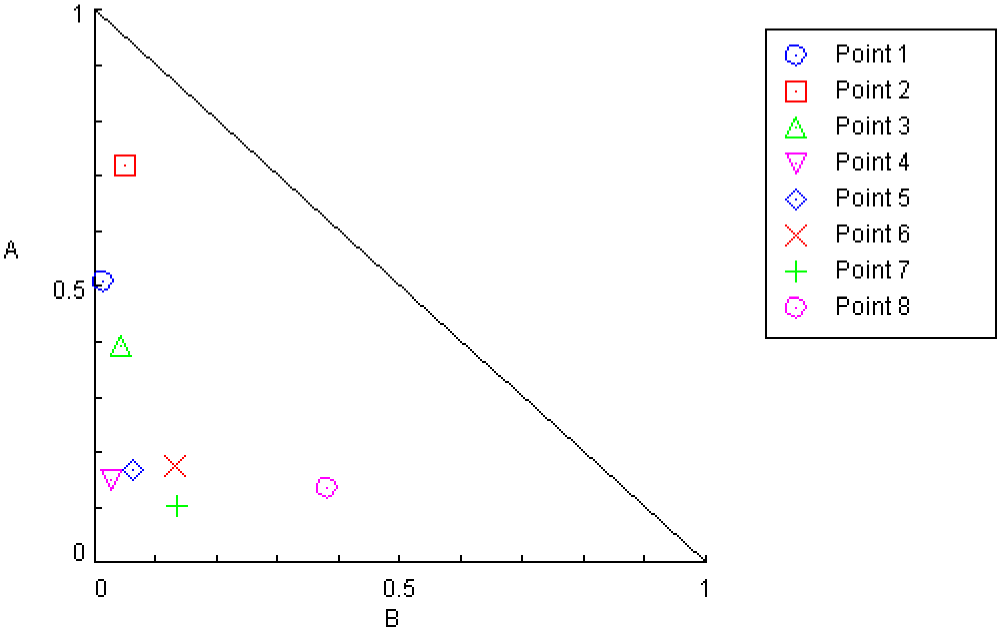

Summary Table

Experiment Name: Artesunate and TP-0903 simultaneous combination
Date:
File Name: C:\Users\MIRKO TERRAGNO\Desktop\MTT\Combin\Low-report\Low.cse
Description Artesunate and TP-0903 simultaneous combination

Drug: Artesunate (A) [uM]
Drug: TP-0903 (T) [uM]
Drug Combo: A 2.5 - T (AT2.5) (A+T)
Drug Combo: A 5 - T (AT5) (A+T)
Drug Combo: A 10 - T (AT 10) (A+T)
Drug Combo: A 20 - T (AT20) (A+T)

Drug Combo: A 40 - T (AT40) (A+T)
Drug Combo: A 80 - T (AT80) (A+T)
Drug Combo: A 160 - T (AT160) (A+T)
Drug Combo: A 320 - T (AT320) (A+T)

Drug/Combo	Dm	m	r
A	52.1694	0.89193	0.95848
T	0.03719	0.68744	0.93217

CI values at:

Combo ED50 ED75 ED90 ED95

Data for Fa = 0.5

Drug/Combo	CI value	Dose A	Dose T
A		52.1694	
T			0.03719

Data for Fa = 0.75

Drug/Combo	CI value	Dose A	Dose T
A		178.792	
T			0.18386

Data for Fa = 0.9

Drug/Combo	CI value	Dose A	Dose T
A		612.744	
T			0.90899

Data for Fa = 0.95

Drug/Combo	CI value	Dose A	Dose T
A		1416.15	
T			2.69536

Data for Fa = 0.97

Drug/Combo	CI value	Dose A	Dose T
A		2570.29	
T			5.84116

CompuSyn Report

Experiment Name: Artesunate and TP0903 sequential combination
Date: 12.11.2021
File Name: C:\Users\MIRKO TERRAGNO\Desktop\MTT\Combin\Low-report\Low.cse
Description Artesunate and TP0903 sequential combination

Drug: Artesunate (A) [uM]
Drug: TP0903 (T) [uM]
Drug Combo: A 2.5 - T (AT2.5) (A+T)
Drug Combo: A 5 - T (AT5) (A+T)
Drug Combo: A 10 - T (AT10) (A+T)
Drug Combo: A 20 - T (AT20) (A+T)
Drug Combo: A 40 - T (AT40) (A+T)
Drug Combo: A 80 - T (AT80) (A+T)
Drug Combo: A 160 - T (AT160) (A+T)
Drug Combo: A 320 - T (AT320) (A+T)

Data for Drug: A [uM]

Dose	Effect
2.5	0.08332
5.0	0.08194
10.0	0.19984
20.0	0.29279
40.0	0.33012
80.0	0.46217
160.0	0.49499
320.0	0.76204

8 data points entered.
X-int: 1.97831
Y-int: -1.3862 +/- 0.10175
m: 0.70067 +/- 0.06331
Dm: 95.1288
r: 0.97637

Data for Drug: T [uM]

Dose	Effect
0.015	0.29633
0.03	0.44193
0.06	0.55374
0.125	0.65789
0.25	0.83297
0.5	0.89959
1.0	0.92297
2.0	0.90312

8 data points entered.
X-int: -1.3868

**Y-int:**	0.99406 +/- 0.07452
**m:**	0.71678 +/- 0.07230
**Dm:**	0.04103
**r:**	0.97081

Data for Non-Constant Combo: AT2.5 (A+T)

Dose A	Dose T	Effect
2.5	0.015	0.36785
2.5	0.03	0.52184
2.5	0.06	0.54260
2.5	0.125	0.58557
2.5	0.25	0.88752
2.5	0.5	0.86290
2.5	1.0	0.87690
2.5	2.0	0.93193

8 data points entered.

Data for Non-Constant Combo: AT5 (A+T)

Dose A	Dose T	Effect
5.0	0.015	0.27130
5.0	0.03	0.49698
5.0	0.06	0.58146
5.0	0.125	0.63698
5.0	0.25	0.84721
5.0	0.5	0.90225
5.0	1.0	0.90297
5.0	2.0	0.92204

8 data points entered.

Data for Non-Constant Combo: AT10 (A+T)

Dose A	Dose T	Effect
10.0	0.015	0.41052
10.0	0.03	0.47389
10.0	0.06	0.60318
10.0	0.125	0.66180
10.0	0.25	0.88367
10.0	0.5	0.90650
10.0	1.0	0.90212
10.0	2.0	0.89299

8 data points entered.

Data for Non-Constant Combo: AT20 (A+T)

Dose A	Dose T	Effect
20.0	0.015	0.37473
20.0	0.03	0.59880
20.0	0.06	0.62838
20.0	0.125	0.64828
20.0	0.25	0.91070

20.0	0.5	0.91764
20.0	1.0	0.92002
20.0	2.0	0.93316

8 data points entered.

Data for Non-Constant Combo: AT40 (A+T)

Dose A	Dose T	Effect
40.0	0.015	0.48098
40.0	0.03	0.53649
40.0	0.06	0.64883
40.0	0.125	0.67205
40.0	0.25	0.89673
40.0	0.5	0.90668
40.0	1.0	0.93963
40.0	2.0	0.91707

8 data points entered.

Data for Non-Constant Combo: AT80 (A+T)

Dose A	Dose T	Effect
80.0	0.015	0.45776
80.0	0.03	0.56745
80.0	0.06	0.65745
80.0	0.125	0.71871
80.0	0.25	0.96042
80.0	0.5	0.97103
80.0	1.0	0.85913
80.0	2.0	0.93521

8 data points entered.

Data for Non-Constant Combo: AT160 (A+T)

Dose A	Dose T	Effect
160.0	0.015	0.59338
160.0	0.03	0.60242
160.0	0.06	0.67187
160.0	0.125	0.72897
160.0	0.25	0.91494
160.0	0.5	0.92659
160.0	1.0	0.92729
160.0	2.0	0.93312

8 data points entered.

Data for Non-Constant Combo: AT320 (A+T)

Dose A	Dose T	Effect
320.0	0.015	0.76835
320.0	0.03	0.79842
320.0	0.06	0.87089
320.0	0.125	0.91191
320.0	0.25	0.97506

320.0	0.5	0.98206
320.0	1.0	0.96271
320.0	2.0	0.97203

8 data points entered.

Dose-Effect Curve for Drugs

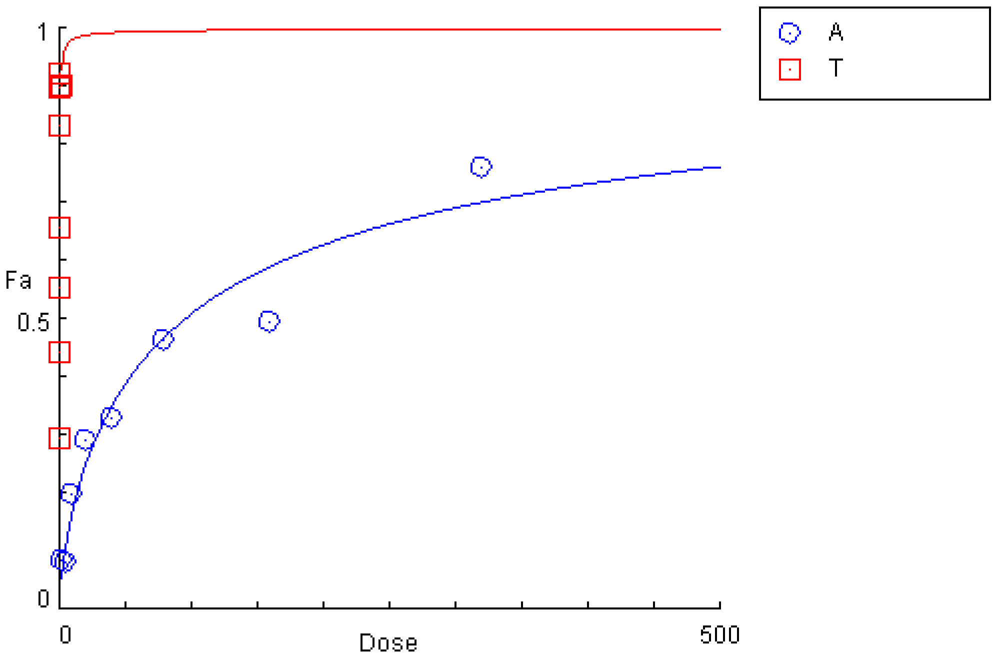

Dose-Effect Curve for Drug Combos

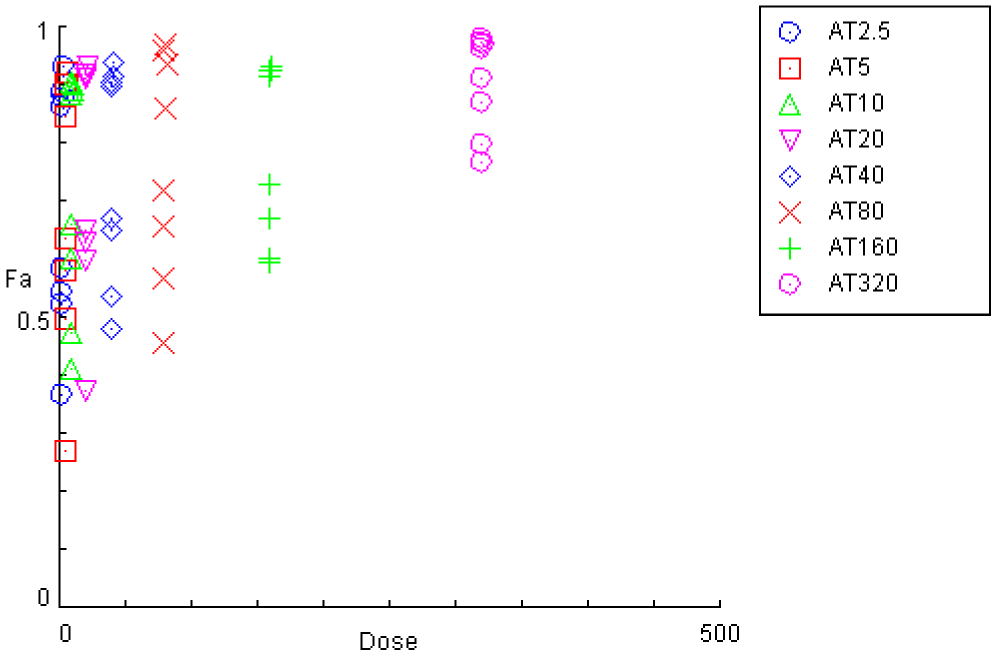

Median-Effect Plot for Drugs

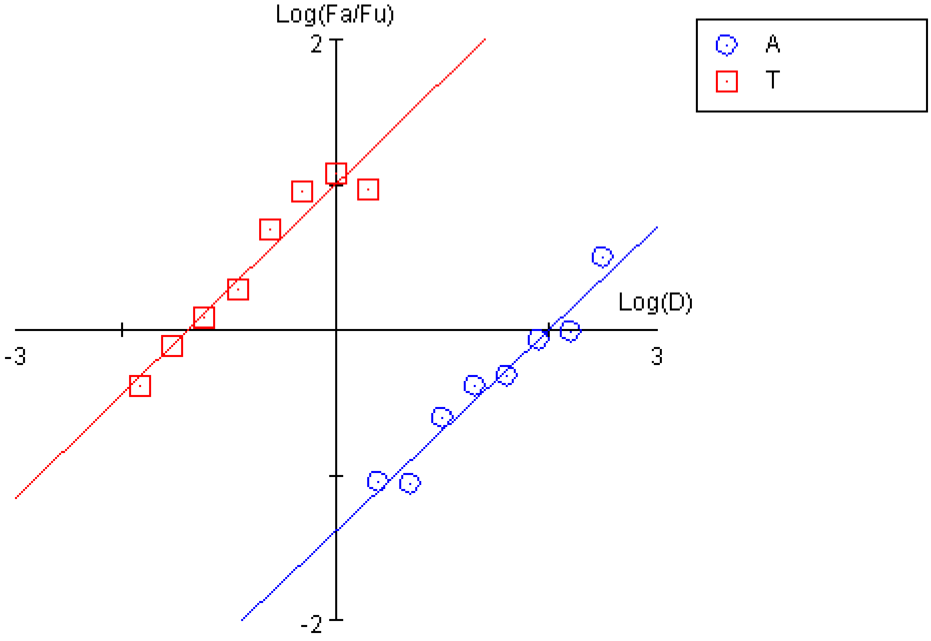

Median-Effect Plot for Drug Combos

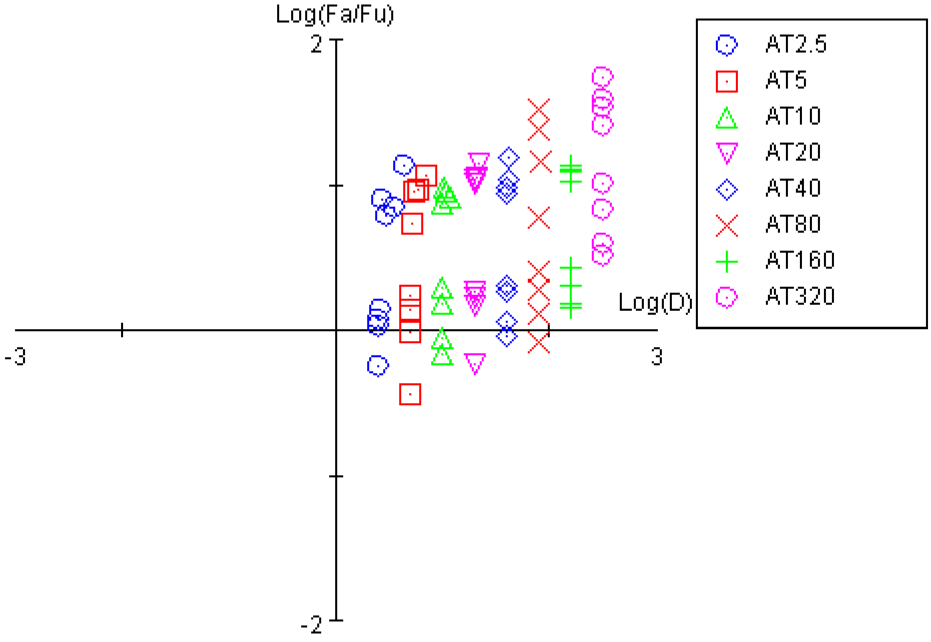

CI Data for Non-Constant Combo: AT2.5 (A+T)

**Dose A**	**Dose T**	**Effect**	**CI**
2.5	0.015	0.36785	0.83497
2.5	0.03	0.52184	0.67033
2.5	0.06	0.54260	1.17272
2.5	0.125	0.58557	1.89675
2.5	0.25	0.88752	0.34273
2.5	0.5	0.86290	0.93776
2.5	1.0	0.87690	1.57639
2.5	2.0	0.93193	1.26647

CI Data for Non-Constant Combo: AT5 (A+T)

**Dose A**	**Dose T**	**Effect**	**CI**

5.0	0.015	0.27130	1.66605
5.0	0.03	0.49698	0.79698
5.0	0.06	0.58146	0.95713
5.0	0.125	0.63698	1.41377
5.0	0.25	0.84721	0.56296
5.0	0.5	0.90225	0.55080
5.0	1.0	0.90297	1.08684
5.0	2.0	0.92204	1.55427

CI Data for Non-Constant Combo: AT10 (A+T)

**Dose A**	**Dose T**	**Effect**	**CI**
10.0	0.015	0.41052	0.78175
10.0	0.03	0.47389	0.96794
10.0	0.06	0.60318	0.87309
10.0	0.125	0.66180	1.23435
10.0	0.25	0.88367	0.36574
10.0	0.5	0.90650	0.51630
10.0	1.0	0.90212	1.10382
10.0	2.0	0.89299	2.53080

CI Data for Non-Constant Combo: AT20 (A+T)

**Dose A**	**Dose T**	**Effect**	**CI**
20.0	0.015	0.37473	1.18330
20.0	0.03	0.59880	0.53687
20.0	0.06	0.62838	0.80200
20.0	0.125	0.64828	1.38577
20.0	0.25	0.91070	0.24629
20.0	0.5	0.91764	0.42860
20.0	1.0	0.92002	0.81350
20.0	2.0	0.93316	1.23666

CI Data for Non-Constant Combo: AT40 (A+T)

**Dose A**	**Dose T**	**Effect**	**CI**
40.0	0.015	0.48098	0.87522
40.0	0.03	0.53649	0.93747
40.0	0.06	0.64883	0.79601
40.0	0.125	0.67205	1.27059
40.0	0.25	0.89673	0.31792
40.0	0.5	0.90668	0.52709
40.0	1.0	0.93963	0.53764
40.0	2.0	0.91707	1.71881

CI Data for Non-Constant Combo: AT80 (A+T)

**Dose A**	**Dose T**	**Effect**	**CI**
80.0	0.015	0.45776	1.53387
80.0	0.03	0.56745	1.07147
80.0	0.06	0.65745	0.92046

80.0	0.125	0.71871	1.04344
80.0	0.25	0.96042	0.08010
80.0	0.5	0.97103	0.09634
80.0	1.0	0.85913	2.01946
80.0	2.0	0.93521	1.19460

CI Data for Non-Constant Combo: AT160 (A+T)

Dose A	Dose T	Effect	CI
160.0	0.015	0.59338	1.19645
160.0	0.03	0.60242	1.33887
160.0	0.06	0.67187	1.14278
160.0	0.125	0.72897	1.17589
160.0	0.25	0.91494	0.27824
160.0	0.5	0.92659	0.39958
160.0	1.0	0.92729	0.74326
160.0	2.0	0.93312	1.27218

CI Data for Non-Constant Combo: AT320 (A+T)

Dose A	Dose T	Effect	CI
320.0	0.015	0.76835	0.67627
320.0	0.03	0.79842	0.57889
320.0	0.06	0.87089	0.32259
320.0	0.125	0.91191	0.23659
320.0	0.25	0.97506	0.05456
320.0	0.5	0.98206	0.05691
320.0	1.0	0.96271	0.29371
320.0	2.0	0.97203	0.36632

Combination Index Plot

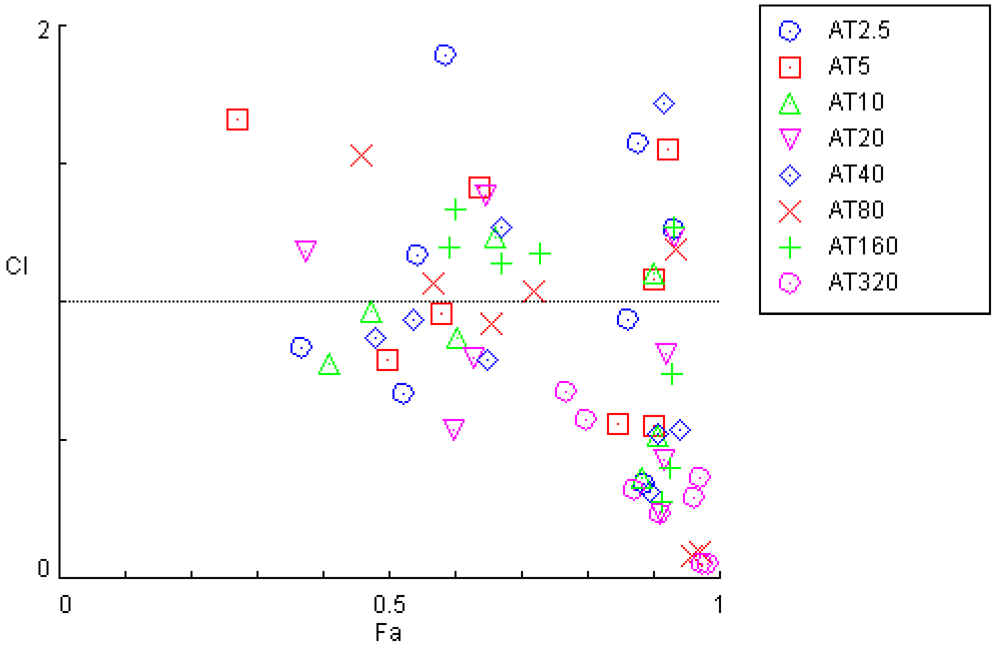

Logarithmic Combination Index Plot

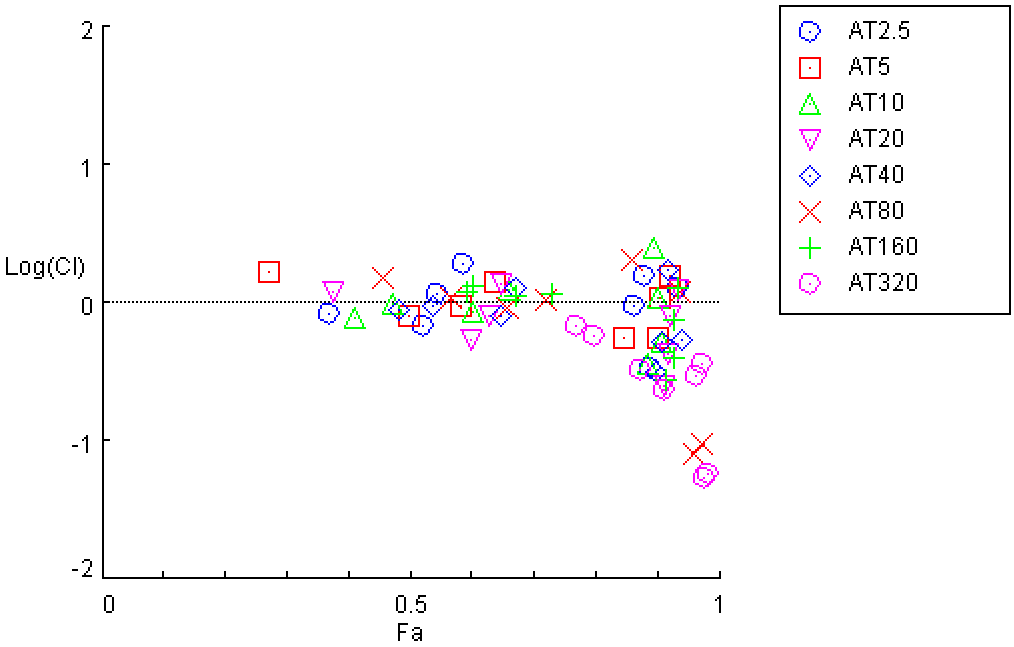

DRI Data for Non-Constant Combo: AT2.5 (A+T)

Fa	Dose A	Dose T	DRI A	DRI T
0.36785	43.9243	0.01928	17.5697	1.28526
0.52184	107.772	0.04636	43.1086	1.54527
0.54260	121.393	0.05208	48.5570	0.86796
0.58557	155.799	0.06646	62.3195	0.53171
0.88752	1814.10	0.73238	725.641	2.92952
0.86290	1313.83	0.53427	525.532	1.06854
0.87690	1567.76	0.63500	627.103	0.63500
0.93193	3983.36	1.57997	1593.34	0.78999

DRI Data for Non-Constant Combo: AT5 (A+T)

Fa	Dose A	Dose T	DRI A	DRI T
0.27130	23.2223	0.01034	4.64447	0.68930
0.49698	93.5044	0.04035	18.7009	1.34498
0.58146	152.090	0.06492	30.4179	1.08195
0.63698	212.237	0.08991	42.4475	0.71932
0.84721	1096.50	0.44771	219.300	1.79084
0.90225	2268.94	0.91141	453.788	1.82283
0.90297	2295.77	0.92195	459.154	0.92195
0.92204	3232.18	1.28806	646.437	0.64403

DRI Data for Non-Constant Combo: AT10 (A+T)

Fa	Dose A	Dose T	DRI A	DRI T
0.41052	56.7606	0.02477	5.67606	1.65132
0.47389	81.9424	0.03547	8.19424	1.18217
0.60318	172.921	0.07360	17.2921	1.22660
0.66180	247.975	0.10469	24.7975	0.83751
0.88367	1718.40	0.69459	171.840	2.77836
0.90650	2434.06	0.97620	243.406	1.95239

0.90212	2264.29	0.90958	226.429	0.90958
0.89299	1964.95	0.79186	196.495	0.39593

DRI Data for Non-Constant Combo: AT20 (A+T)

Fa	Dose A	Dose T	DRI A	DRI T
0.37473	45.8105	0.02009	2.29053	1.33919
0.59880	168.469	0.07174	8.42343	2.39144
0.62838	201.319	0.08539	10.0659	1.42317
0.64828	227.685	0.09631	11.3843	0.77046
0.91070	2616.28	1.04758	130.814	4.19030
0.91764	2968.48	1.18523	148.424	2.37047
0.92002	3106.45	1.23906	155.323	1.23906
0.93316	4096.09	1.62367	204.804	0.81184

DRI Data for Non-Constant Combo: AT40 (A+T)

Fa	Dose A	Dose T	DRI A	DRI T
0.48098	85.3371	0.03690	2.13343	2.46005
0.53649	117.203	0.05032	2.93007	1.67733
0.64883	228.464	0.09663	5.71159	1.61048
0.67205	264.858	0.11165	6.62145	0.89320
0.89673	2079.63	0.83700	51.9907	3.34801
0.90668	2441.33	0.97905	61.0332	1.95809
0.93963	4783.04	1.88938	119.576	1.88938
0.91707	2936.85	1.17289	73.4213	0.58644

DRI Data for Non-Constant Combo: AT80 (A+T)

Fa	Dose A	Dose T	DRI A	DRI T
0.45776	74.7036	0.03240	0.93379	2.15996
0.56745	140.140	0.05993	1.75175	1.99755
0.65745	241.220	0.10190	3.01525	1.69833
0.71871	362.868	0.15189	4.53585	1.21511
0.96042	9013.55	3.51018	112.669	14.0407
0.97103	14296.1	5.50999	178.701	11.0200
0.85913	1256.09	0.51131	15.7011	0.51131
0.93521	4295.03	1.70072	53.6879	0.85036

DRI Data for Non-Constant Combo: AT160 (A+T)

Fa	Dose A	Dose T	DRI A	DRI T
0.59338	163.148	0.06953	1.01967	4.63516
0.60242	172.146	0.07327	1.07591	2.44245
0.67187	264.555	0.11153	1.65347	1.85876
0.72897	390.447	0.16316	2.44029	1.30531
0.91494	2822.86	1.12837	17.6429	4.51347
0.92659	3546.95	1.41055	22.1685	2.82110
0.92729	3599.59	1.43101	22.4974	1.43101
0.93312	4091.69	1.62196	25.5731	0.81098

DRI Data for Non-Constant Combo: AT320 (A+T)

Fa	Dose A	Dose T	DRI A	DRI T
0.76835	526.623	0.21860	1.64570	14.5730
0.79842	678.343	0.27998	2.11982	9.33254
0.87089	1450.38	0.58849	4.53243	9.80816
0.91191	2672.68	1.06965	8.35213	8.55719
0.97506	17811.7	6.83116	55.6617	27.3246
0.98206	28777.8	10.9185	89.9306	21.8371
0.96271	9849.85	3.82823	30.7808	3.82823
0.97203	15055.6	5.79597	47.0487	2.89798

DRI Plot for Non-Constant Combo: AT2.5 (A+T)

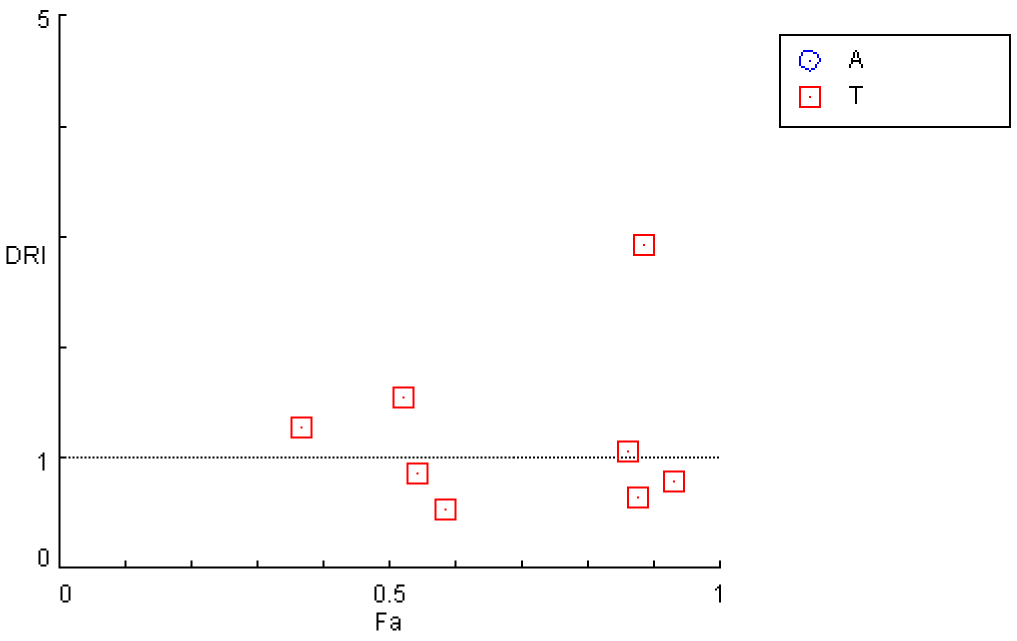

DRI Plot for Non-Constant Combo: AT5 (A+T)

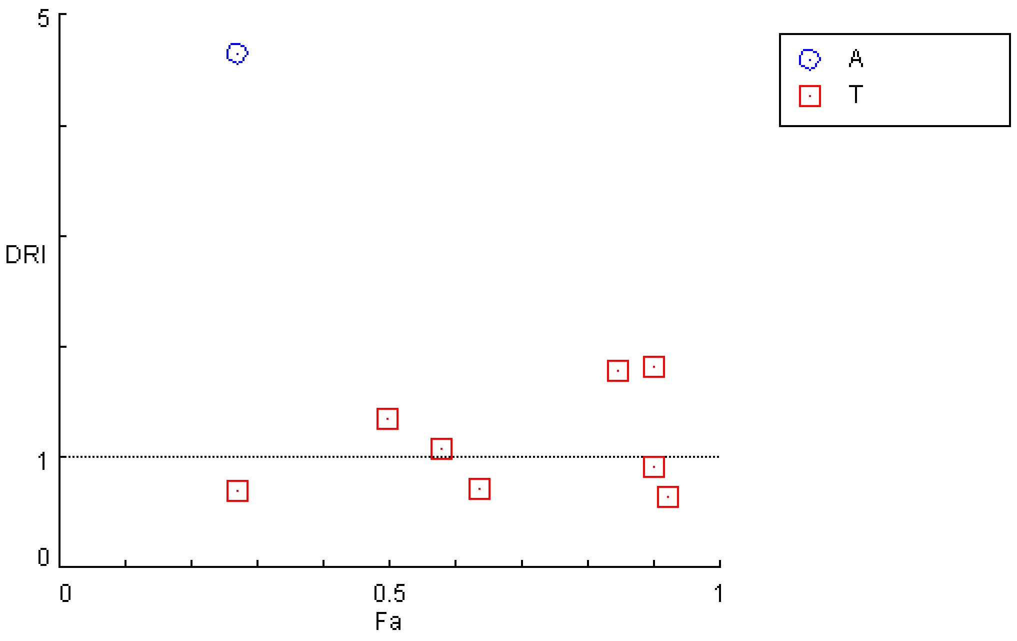

DRI Plot for Non-Constant Combo: AT10 (A+T)

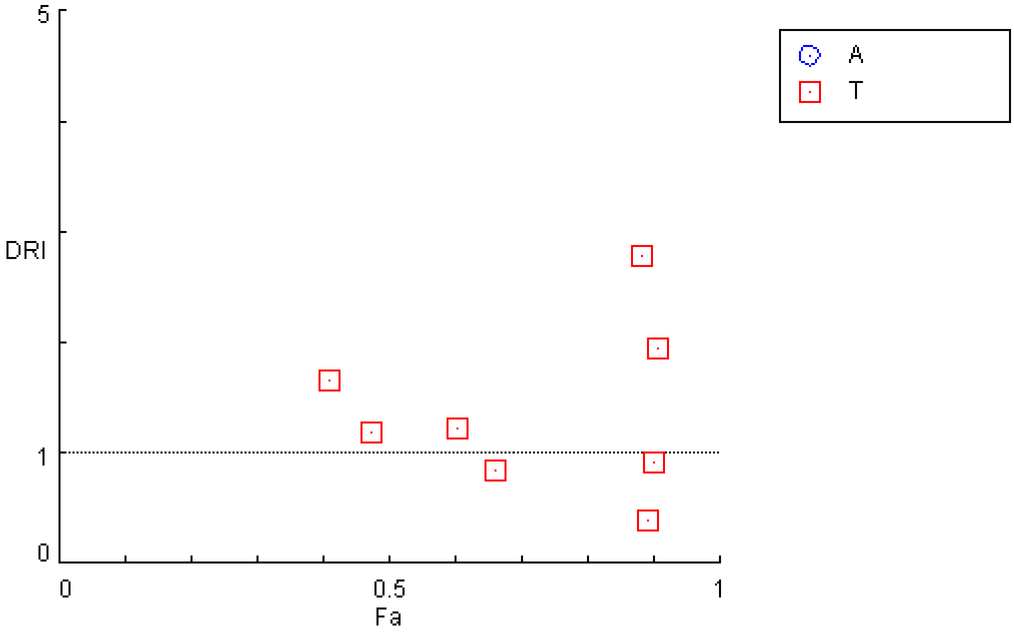

DRI Plot for Non-Constant Combo: AT20 (A+T)

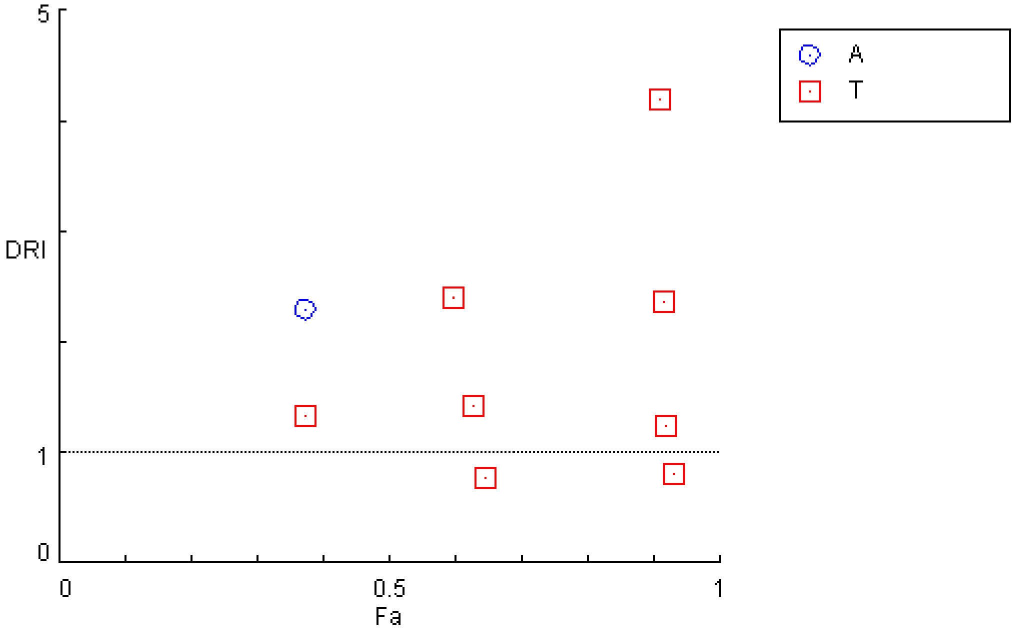

DRI Plot for Non-Constant Combo: AT40 (A+T)

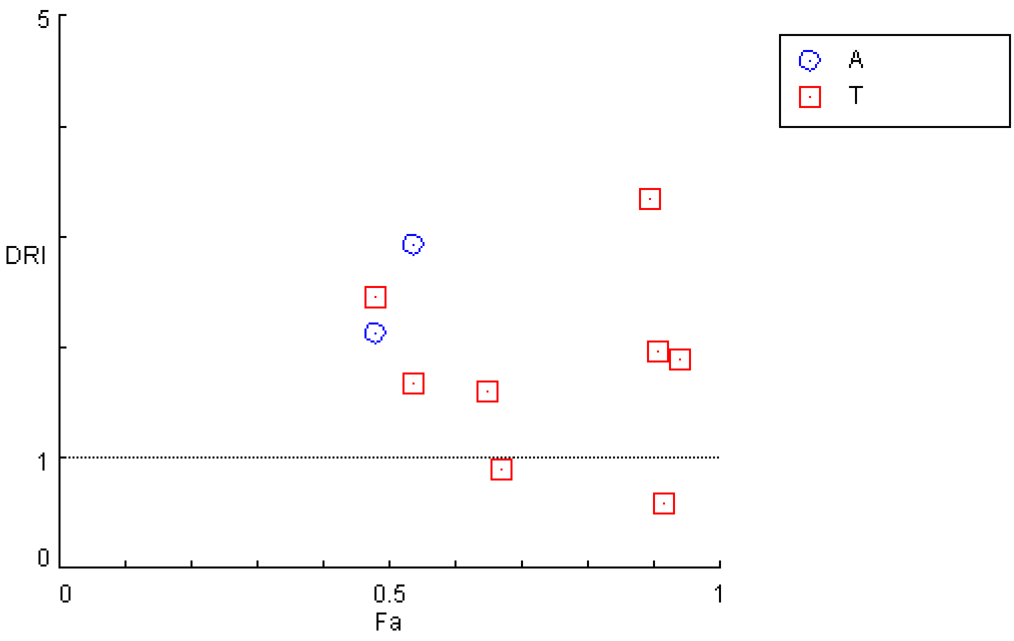

DRI Plot for Non-Constant Combo: AT80 (A+T)

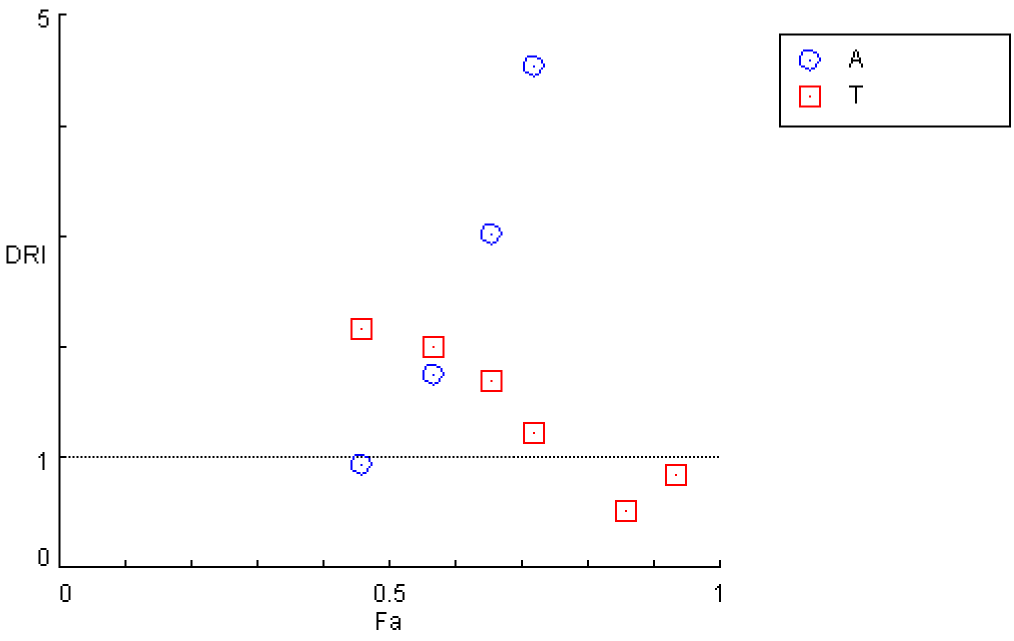

DRI Plot for Non-Constant Combo: AT160 (A+T)

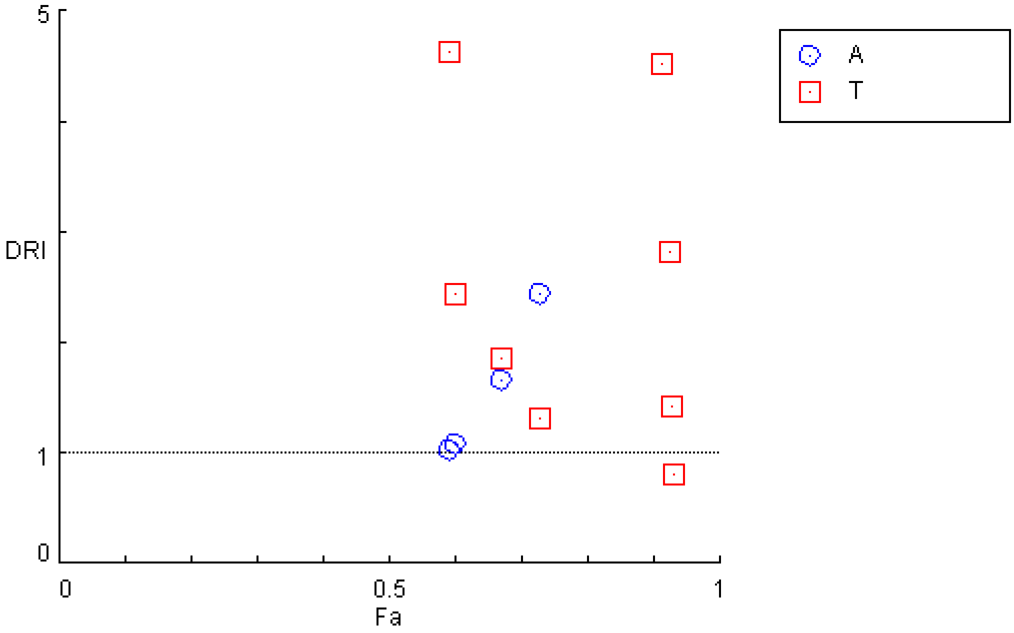

DRI Plot for Non-Constant Combo: AT320 (A+T)

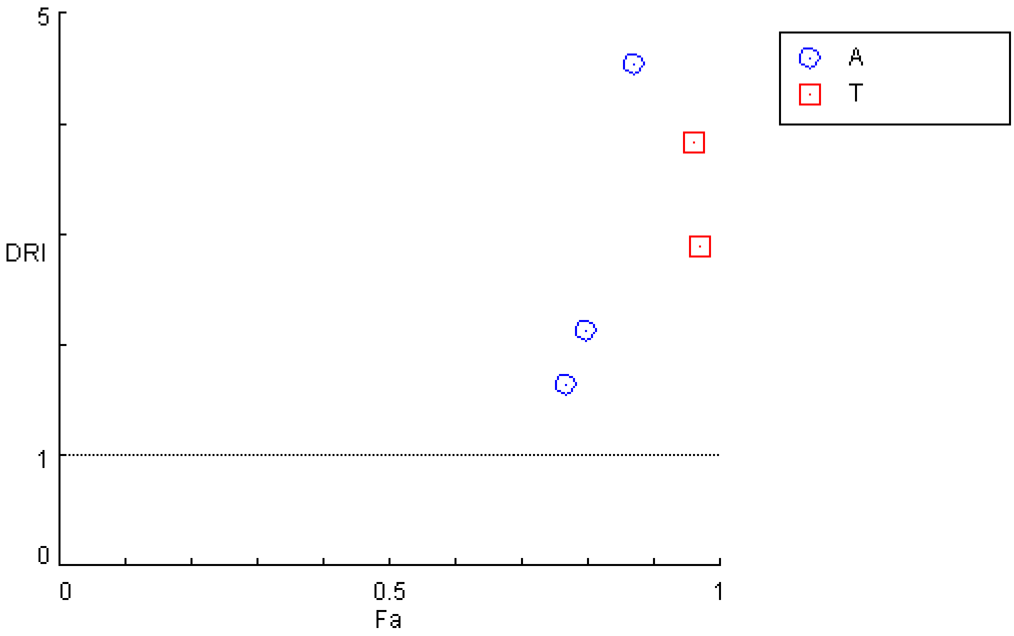

Log(DRI) Plot for Non-Constant Combo: AT2.5 (A+T)

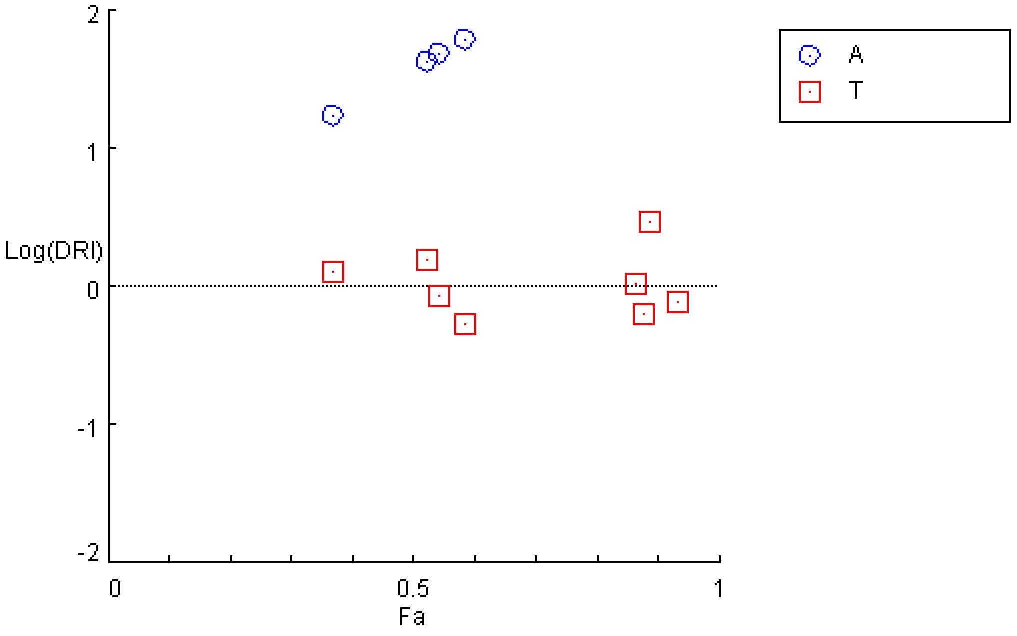

Log(DRI) Plot for Non-Constant Combo: AT5 (A+T)

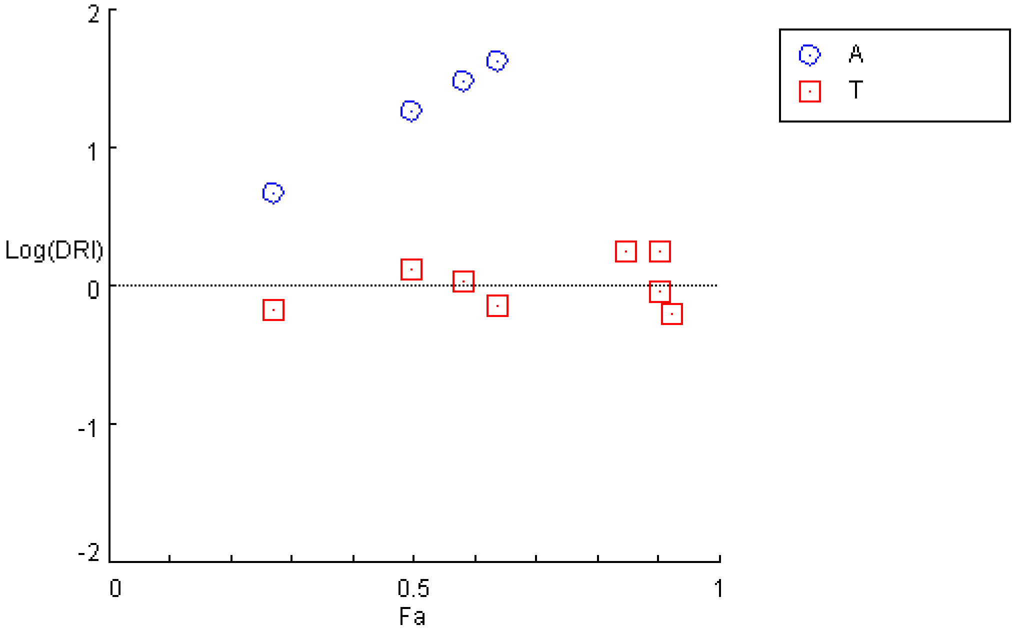

Log(DRI) Plot for Non-Constant Combo: AT10 (A+T)

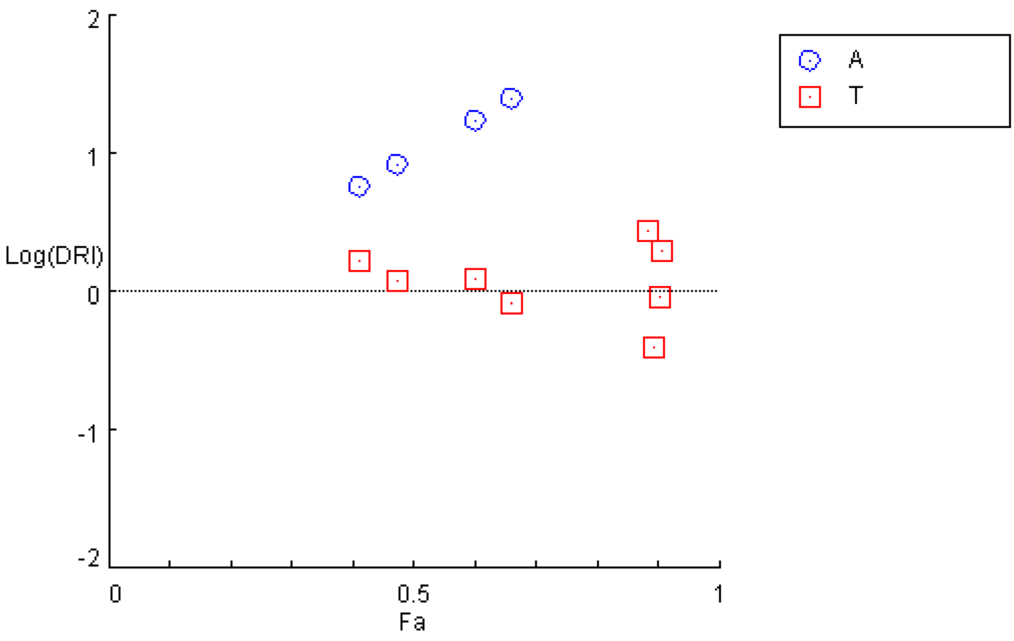

Log(DRI) Plot for Non-Constant Combo: AT20 (A+T)

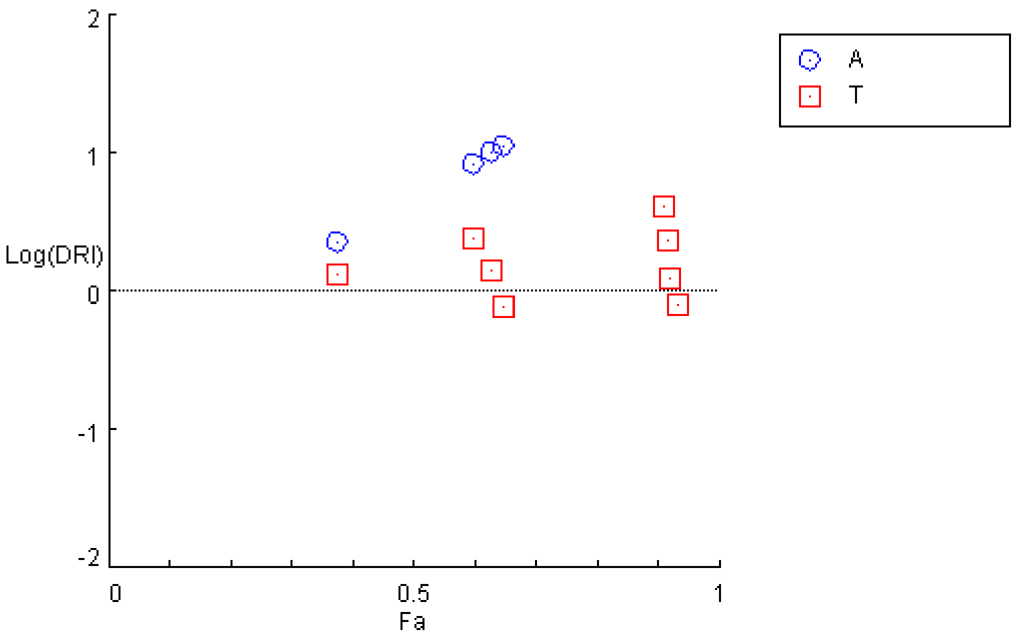

Log(DRI) Plot for Non-Constant Combo: AT40 (A+T)

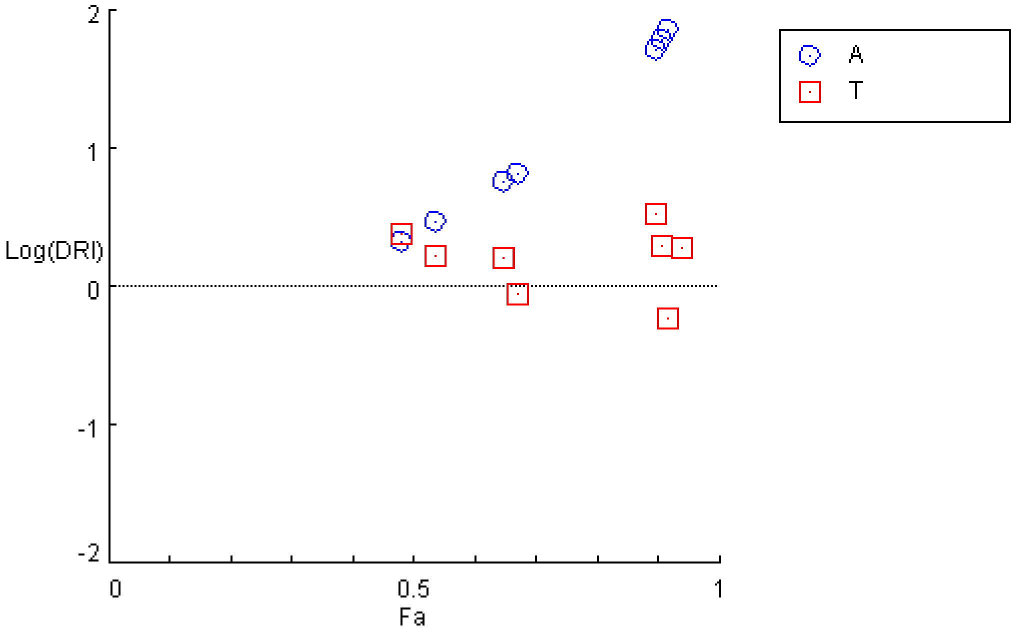

Log(DRI) Plot for Non-Constant Combo: AT80 (A+T)

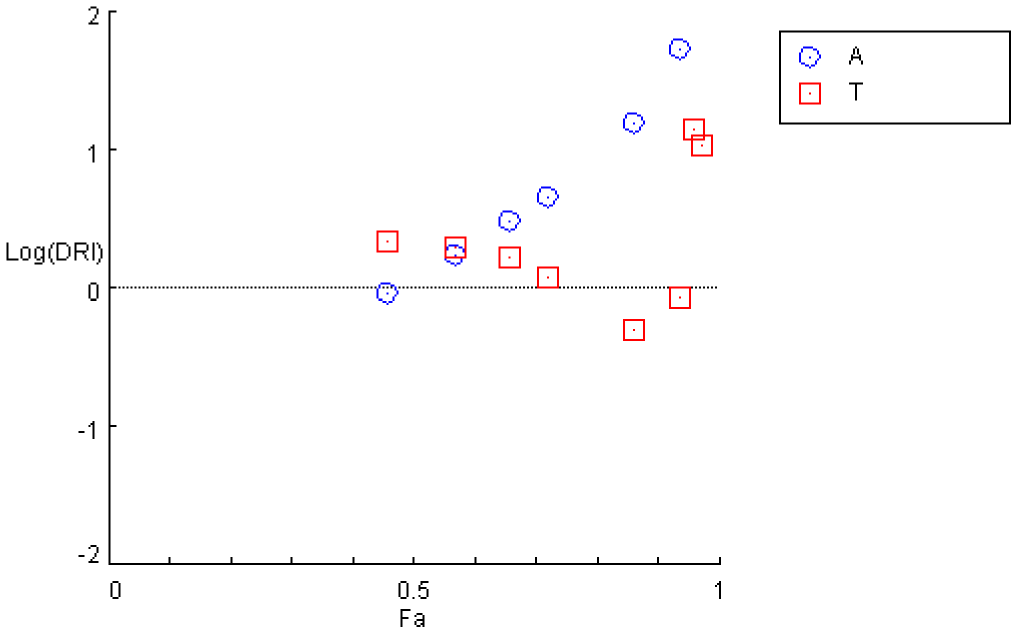

Log(DRI) Plot for Non-Constant Combo: AT160 (A+T)

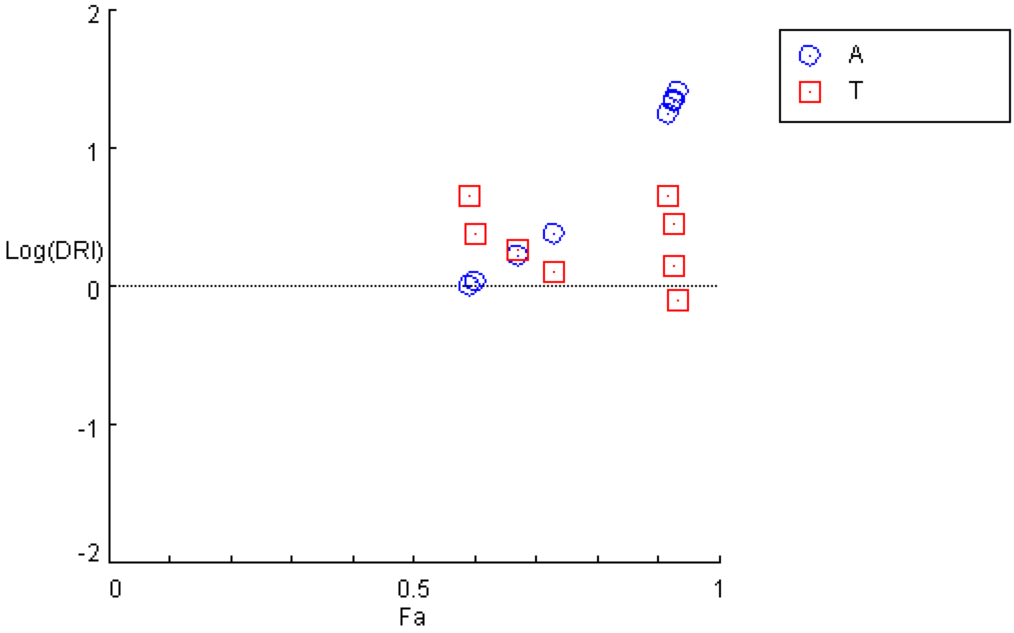

Log(DRI) Plot for Non-Constant Combo: AT320 (A+T)

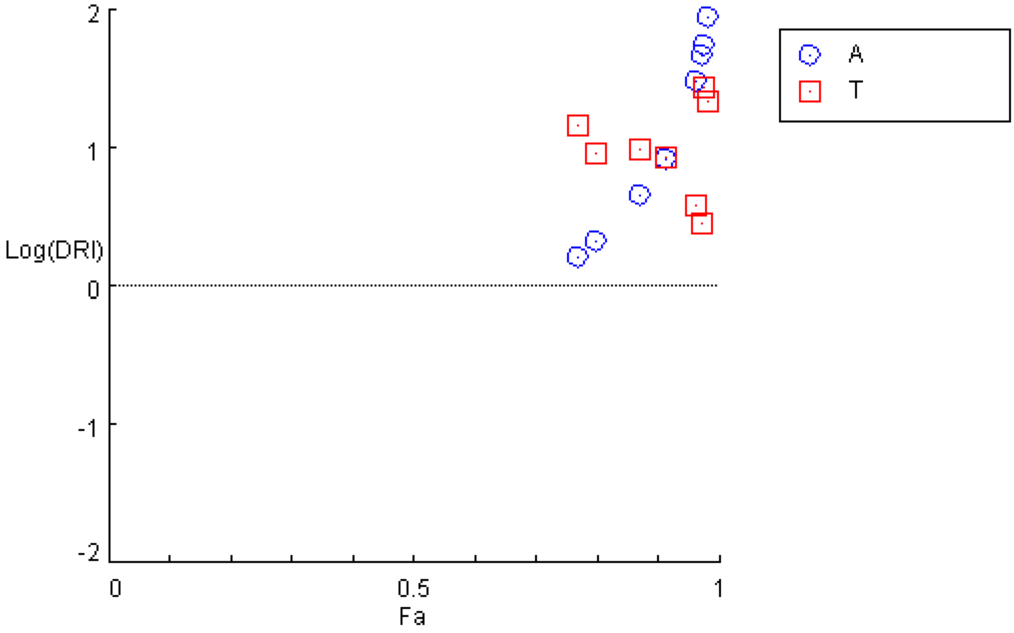

Normalized Isobologram for Combo: AT2.5 (A+T)

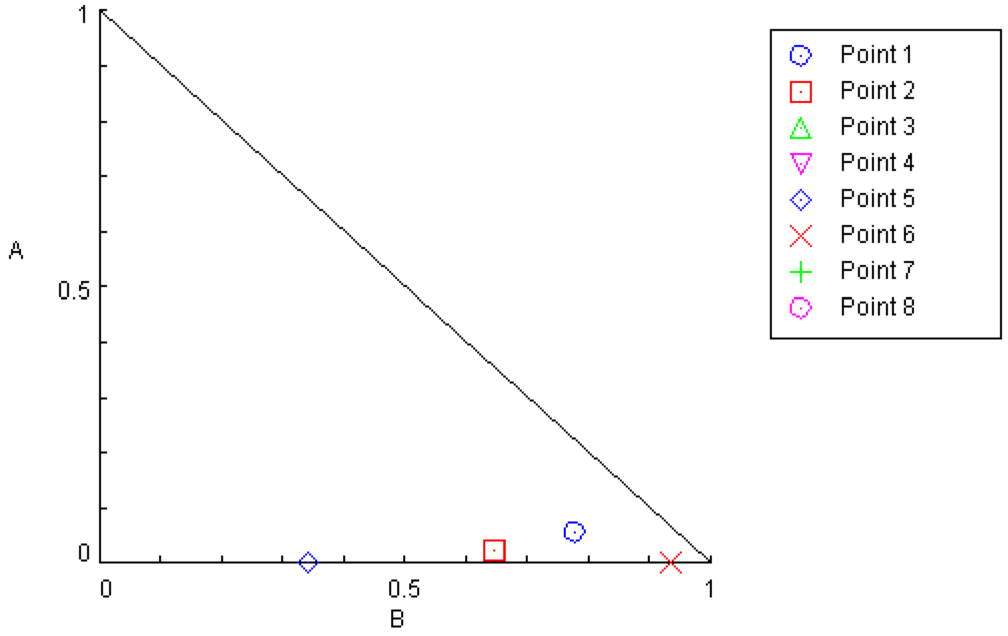

Normalized Isobologram for Combo: AT5 (A+T)

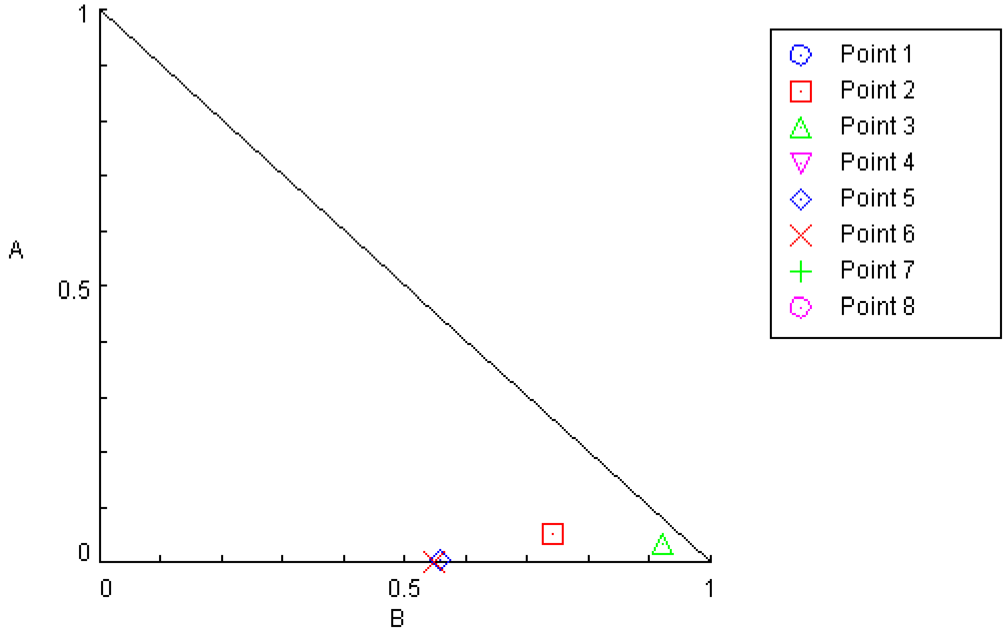

Normalized Isobologram for Combo: AT10 (A+T)

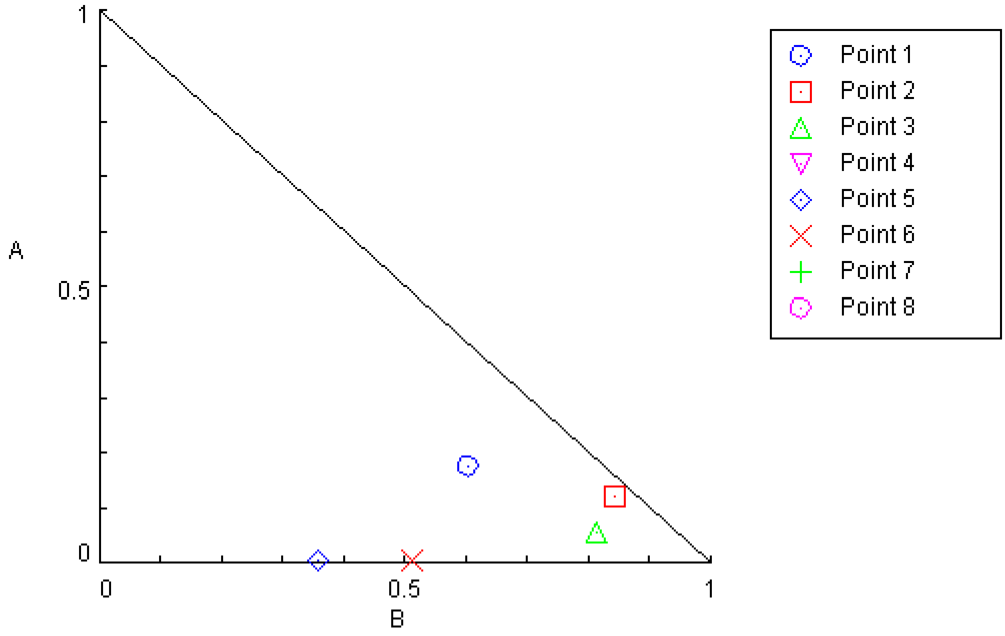

Normalized Isobologram for Combo: AT20 (A+T)

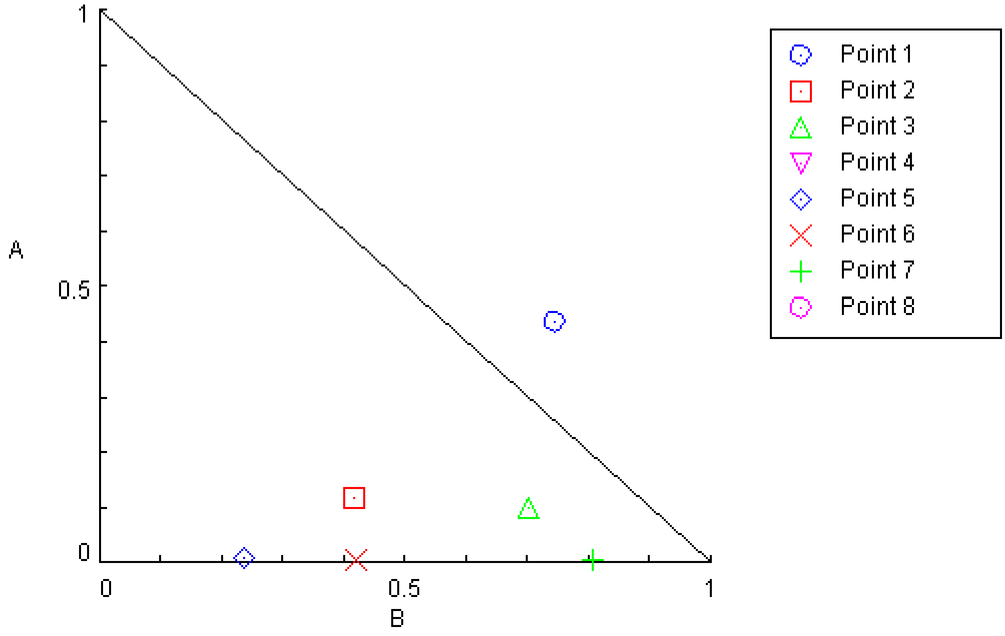

Normalized Isobologram for Combo: AT40 (A+T)

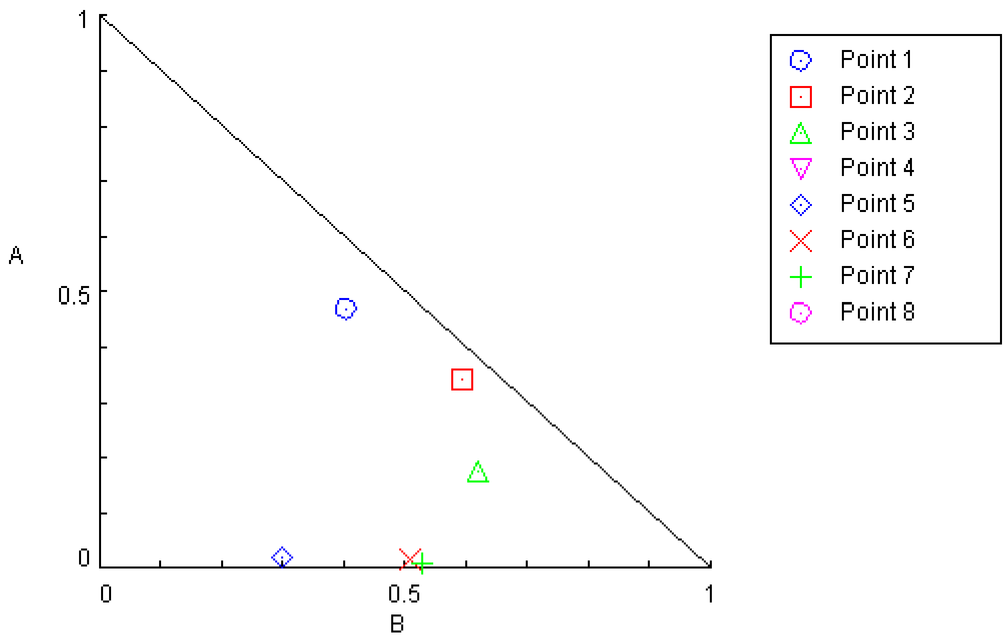

Normalized Isobologram for Combo: AT80 (A+T)

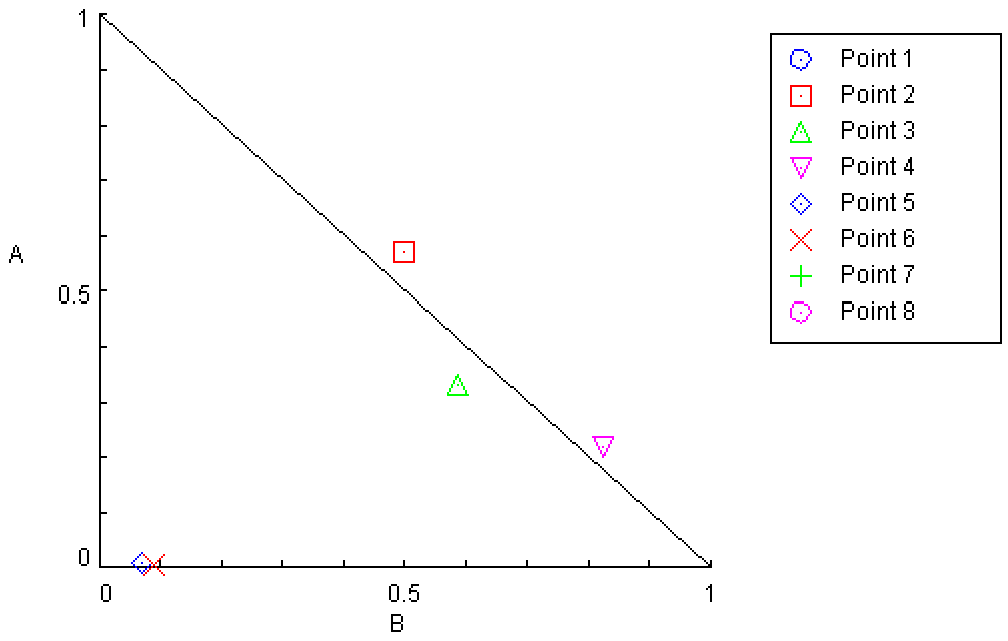

Normalized Isobologram for Combo: AT160 (A+T)

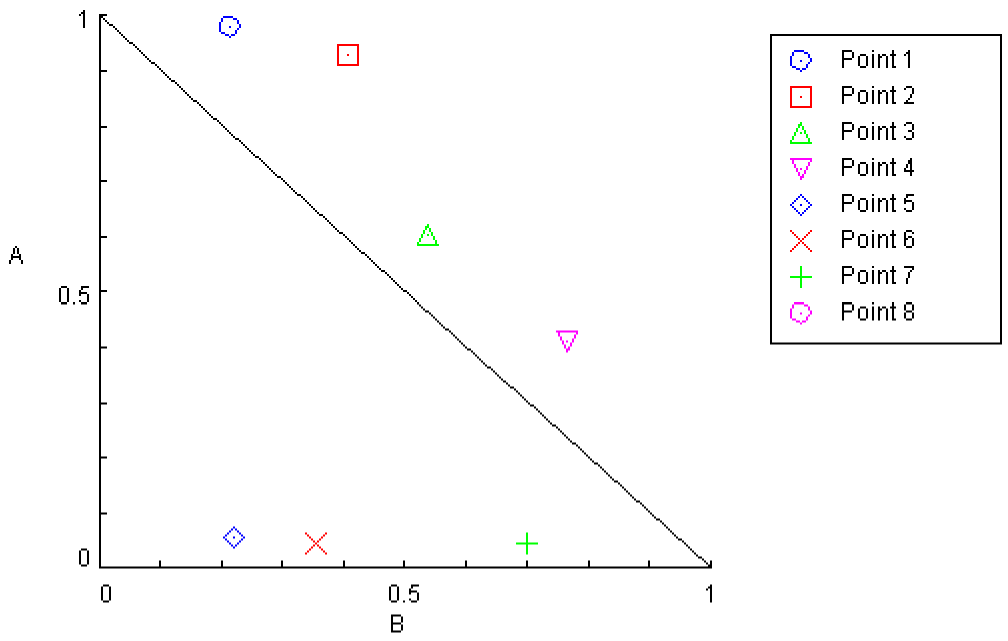

Normalized Isobologram for Combo: AT320 (A+T)

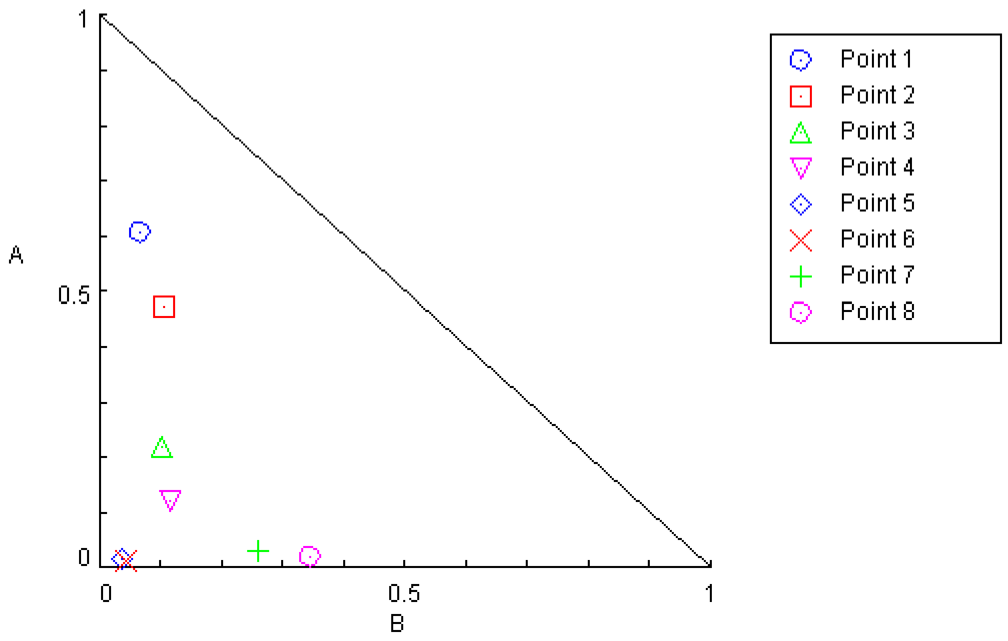

Summary Table

Experiment Name: Artesunate and TP0903 sequential combination
Date: 12.11.2021
File Name: C:\Users\MIRKO TERRAGNO\Desktop\MTT\Combin\Low-report\Low.cse
Description Artesunate and TP0903 sequential combination

Drug: Artesunate (A) [uM]
Drug: TP0903 (T) [uM]
Drug Combo: A 2.5 - T (AT2.5) (A+T)
Drug Combo: A 5 - T (AT5) (A+T)
Drug Combo: A 10 - T (AT10) (A+T)
Drug Combo: A 20 - T (AT20) (A+T)

Drug Combo: A 40 - T (AT40) (A+T)
Drug Combo: A 80 - T (AT80) (A+T)
Drug Combo: A 160 - T (AT160) (A+T)
Drug Combo: A 320 - T (AT320) (A+T)

Drug/Combo	Dm	m	r
A	95.1288	0.70067	0.97637
T	0.04103	0.71678	0.97081

CI values at:

Combo ED50 ED75 ED90 ED95

Data for Fa = 0.5

Drug/Combo	CI value	Dose A	Dose T
A		95.1288	
T			0.04103

Data for Fa = 0.75

Drug/Combo	CI value	Dose A	Dose T
A		456.309	
T			0.19002

Data for Fa = 0.9

Drug/Combo	CI value	Dose A	Dose T
A		2188.80	
T			0.87993

Data for Fa = 0.95

Drug/Combo	CI value	Dose A	Dose T
A		6358.39	
T			2.49566

Data for Fa = 0.97

Drug/Combo	CI value	Dose A	Dose T
A		13579.5	
T			5.23984
